# Dispersal ability determines the scaling properties of species abundance distributions: a case study using arthropods from the Azores

**DOI:** 10.1038/s41598-017-04126-5

**Published:** 2017-06-20

**Authors:** Luís Borda-de-Água, Robert J. Whittaker, Pedro Cardoso, François Rigal, Ana M. C. Santos, Isabel R. Amorim, Aristeidis Parmakelis, Kostas A. Triantis, Henrique M. Pereira, Paulo A. V. Borges

**Affiliations:** 1 Theoretical Ecology and Biodiversity, and Infraestruturas de Portugal Biodiversity Chair, CIBIO/InBio, Centro de Investigação em Biodiversidade e Recursos Genéticos, Laboratório Associado, Universidade do Porto. Campus Agrário de Vairão, 4485-661 Vairão, Portugal; 2 CEABN/InBio, Centro de Ecologia Aplicada “Professor Baeta Neves”, Instituto Superior de Agronomia, Universidade de Lisboa, Tapada da Ajuda, 1349-017 Lisboa Portugal; 3 School of Geography and the Environment, University of Oxford, South Parks Rd, Oxford, OX1 3QY UK; 4 Center for Macroecology, Evolution and Climate, Natural History Museum of Denmark, University of Copenhagen, Copenhagen, Denmark; 5 Finnish Museum of Natural History, University of Helsinki, Helsinki, Finland; 6 cE3c – Centre for Ecology, Evolution and Environmental Changes/Azorean Biodiversity Group and Universidade dos Açores - Departamento de Ciências Agrárias, Rua Capitão João d’Ávila, São Pedro, 9700-042 Angra do Heroísmo, Terceira, Azores Portugal; 7 Environment and Microbiology Team, Université de Pau et des Pays de l’Adour, IPREM UMR CNRS, 5254 Pau Cedex, France; 8 Departament of Biogeography and Global Change, Museo Nacional de Ciencias Naturales (MNCN-CSIC), 28006 Madrid, Spain; 9 Forest Ecology & Restoration Group, Department of Life Sciences, Universidad de Alcalá, 28805 Alcalá de Henares Madrid, Spain; 10 Department of Ecology and Taxonomy, Faculty of Biology, National and Kapodistrian, University of Athens, Athens, GR-15784 Greece; 11 German Centre for Integrative Biodiversity Research (iDiv) Halle-Jena-Leipzig Deutscher Platz 5e, 04103 Leipzig, Germany; 12 Institute of Biology, Martin Luther University Halle-Wittenberg, Am Kirchtor 1, 06108 Halle (Saale), Germany

## Abstract

Species abundance distributions (SAD) are central to the description of diversity and have played a major role in the development of theories of biodiversity and biogeography. However, most work on species abundance distributions has focused on one single spatial scale. Here we used data on arthropods to test predictions obtained with computer simulations on whether dispersal ability influences the rate of change of SADs as a function of sample size. To characterize the change of the shape of the SADs we use the moments of the distributions: the skewness and the raw moments. In agreement with computer simulations, low dispersal ability species generate a hump for intermediate abundance classes earlier than the distributions of high dispersal ability species. Importantly, when plotted as function of sample size, the raw moments of the SADs of arthropods have a power law pattern similar to that observed for the SAD of tropical tree species, thus we conjecture that this might be a general pattern in ecology. The existence of this pattern allows us to extrapolate the moments and thus reconstruct the SAD for larger sample sizes using a procedure borrowed from the field of image analysis based on scaled discrete Tchebichef moments and polynomials.

## Introduction

The number of species and their relative abundance are important components of species diversity and community structure^1–3^. A key ecological question concerning species richness patterns is how the number of species scales as a function of area, i.e. the form taken by the species–area relationship (SAR). This is a well studied pattern^4^. However, and in clear contrast, there has been much less attention to how the abundance of species scales with area^5^, although this is a fundamental question in understanding how patterns of community assembly emerge and their consequences for ecosystem functioning. Here we examine the role of dispersal in determining how the relative abundance of species changes with increasing spatial scale, with a view to enabling the projection of abundance distributions to larger scales.

**Figure 1:**
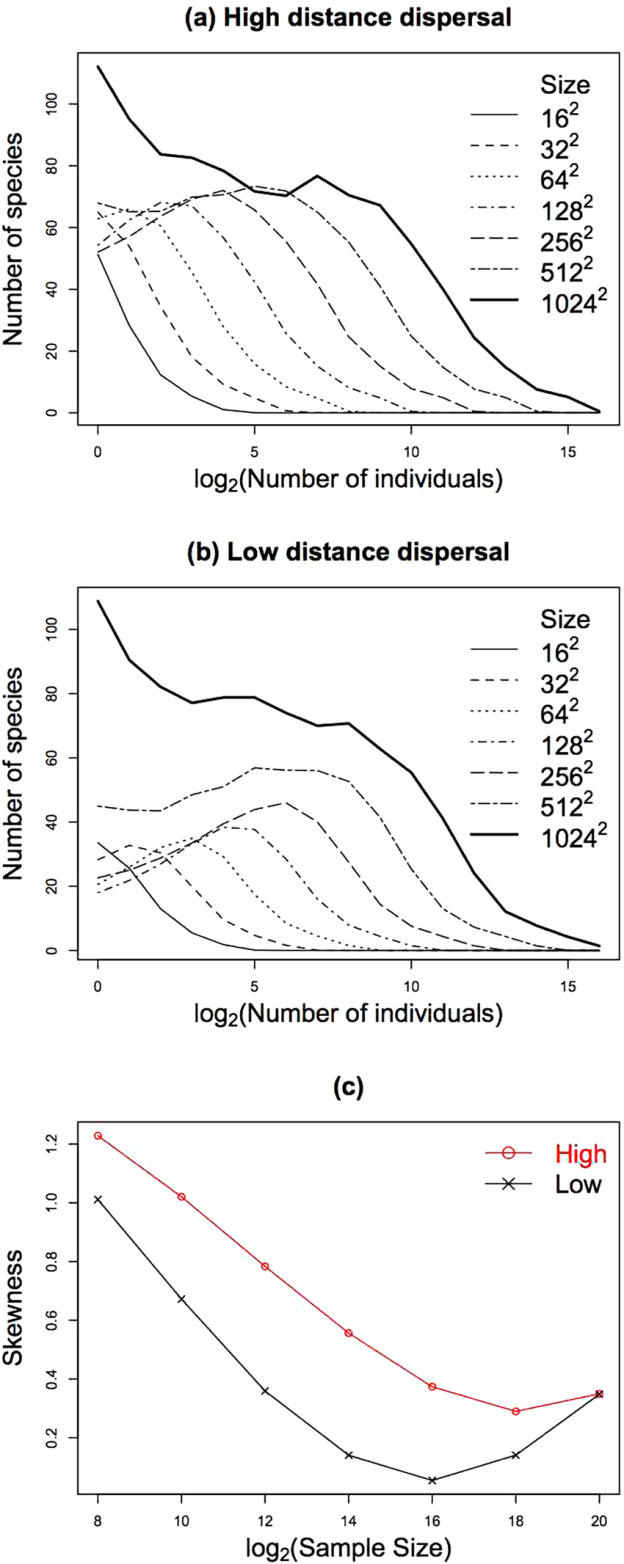
The SADs as a function of sample size obtained with computer simulations: (**a**) high dispersal ability and (**b**) low dispersal ability. In the computer simulations each individual occupied a cell in a landscape modelled as a matrix of 1024 × 1024 elements. Thus sample size (the numbers in the right side of the figure) can be interpreted as the number of individuals or area size (e.g., “16^2^” means a sample of 16 × 16 elements (or individuals, or size) of the landscape. Except for the largest size (1024^2^) each curve is the average obtained from all samples with the same size. Each sample is a set of adjacent points in a matrix forming a square. The x-axis corresponds to classes of the logarithm of the number of individuals as follows: 1 individual, 2 to 3 individuals, 4 to 7 individuals, *et seq*. Plot (**c**) shows the skewness of the high (red) and low (black) dispersal ability distributions as a function of sample size.

The relative abundance of species has played a major role in describing and understanding ecological communities^1, 3^. There are several graphical ways to depict the relative abundance of species^3, 6, 7^. One such approach is the species abundance distribution (SAD), which consists of plotting the size class of the number of individuals (log-transformed) on the *x*-axis and the number of species in each class on the *y*-axis (for instance, Fig. 1a,b). Some of the seminal studies on SADs concerned the scaling properties of these distributions. For example, Preston^1^ emphasized that the shape of the observed distributions was a function of sample size and introduced the concept of the “veil line”, by which he was referring to a line shifting to the left of the distribution revealing additional rarer species as sample size increased. However, more recently, studies on SADs have focused on the statistical distribution that best fits the data at one specific scale^8–11^. Nevertheless, in addition to studying the SAD at one specific scale, there is also merit in describing how the SAD changes as a function of a scaling variable, such as area^5, 12, 13^. Identifying the patterns associated with the scaling properties of the SADs is important because, first, it provides a mathematical framework where experiments can be devised in order to understand the processes generating the observed diversity patterns, and, second, it suggests ways to forecast the distributions for larger areas, with obvious implications for conservation studies. Other authors have recently addressed how to forecast the SAD at large spatial scales^14–18^, but here we use a method^5^ which combines the patterns observed for the moments of the SADs with methods developed in the field of image analysis^19^.

*A priori* knowledge of the importance of natural processes for some species groups may justify the deconstruction of a SAD into the distributions of those groups^20, 21^. Indeed, computer simulations developed in some of our previous work^22^ showed that the distributions of high and low dispersal ability species develop at different paces when sample size increases (Fig. 1, adapted from ref. 22). These results motivated us to explore the deconstruction of the SAD into the distributions of these two groups^6, 23^. These computer simulations were spatially explicit and implemented the basic tenets of neutral theory^24^ (namely, that in a community, all individuals, independently of the species, have the same probability of dying, reproducing, speciating and dispersing), but we assumed two communities: one with high (Fig. 1a) and one with low (Fig. 1b) dispersal ability species (for more details on the simulations, see ref. 22). The important feature revealed by Fig. 1 is that the SADs of both communities are monotonically decreasing functions for small sample sizes, but when sample size increases the maximum of the SADs for low dispersal ability species moves towards intermediate abundance classes faster than that of the SADs of high dispersal ability species, which retain a larger proportion of singletons even for relatively large sample sizes.

The shape of the SADs of low dispersal ability species changes more rapidly with increasing sample size because these species are more aggregated in space. Clustering implies that when sample size increases we tend to find more individuals of the same species, but relatively fewer new species, hence species quickly shift towards intermediate abundance classes as sample size increases. On the other hand, species with high dispersal ability tend to be more spatially mixed, thus when sample size increases we find a large number of new species, but with many more species remaining at low abundances. For very large sample sizes the two distributions are again similar, because for both communities of high and low dispersal ability species, new species are mainly rare ones; intermediate or large abundance species have already been identified (for the role of spatial aggregation in the context of SADs, see also ref. 25). However, as we will see, our empirical data only describe the transitions in the distributions for small sample sizes obtained with the simulations, that is, the change from monotonically decreasing functions to functions with a maximum developing for intermediate abundance classes.

Our main purpose is to quantify how SADs change with scale and for that we use the moments of the empirical data. This means that we do not attempt to fit SADs at a particular scale with a given probability distribution, such as the logseries or the lognormal. Instead, we describe how the moments of the data behave at different scales. Because the terminology associated with moments may not be familiar, we give more detailed background information in Methods, but here we point out that moments are often used as common estimators in statistics. For instance, the mean is the first (raw) moment, the variance is the second central moment, and the skewness is the third standard moment^26^. In this paper we pay special attention to the change of the skewness of the distributions when sample size changes. We focus on the skewness of the distributions because it provides a quantitative description of the changes in the symmetry of the distributions, which is the most obvious difference among SADs at different scales, such as, when we move from a logseries-like to a lognormal-like distribution. For instance, the change in symmetry is evident in the shapes of the distributions in Fig. 1a,b as sample size increases and, as we will see, the skewness reveals the different pace of change of the shape of the distributions for low and high dispersal ability species (Fig. 1c).

It is worth exploring the parallels between the methodology we use to describe the scaling properties of a SAD and the one that is typically adopted when studying the species richness for different areas, the SAR. When dealing with the SAR we are concerned with the number of species, thus, for a given area, we have a single value. When we plot the change in the number of species as a function of area we obtain one curve. On the other hand, when dealing with abundances, for a given area we have a distribution, the SAD. In our method we substitute the information contained in the distribution by its moments (e.g. mean, variance, skewness), thus, for a given area, we have several values (the moments). If we are interested in how the SAD changes as a function of area we plot the moments. Therefore, while for the SAR we have only one curve, for the SAD–area relationship we have several curves, one for each moment. Although the interpretation of the curves for the moments may not be as straightforward as that for the SAR, the statistical methods of analysis of the moments–area relationship can proceed in ways similar to those of the SAR^4^.

The aims of this paper are threefold: (i) to test whether a pattern concerning the different scaling of the SADs as a function of area (or sample size) of high- and low-dispersal ability species predicted by computer simulations holds for an island arthropod assemblage; (ii) to extrapolate the SAD to large spatial scales, hitherto not obtained, and thus to identify how the relative abundance of arthropods scales as a function of area or sample size; and (iii) to test whether the pattern for the raw moments previously identified for hyper-diverse tropical tree species communities^5^ also applies to less diverse island arthropod assemblages and therefore gathering evidence on whether this may be a general pattern and motivate researchers to assess whether other datasets on other taxa yield similar results. Underlying these objectives is the overarching idea of exploring patterns that may emerge from examining ecological phenomena at different scales.

## Methods

### Data

**Figure 2:**
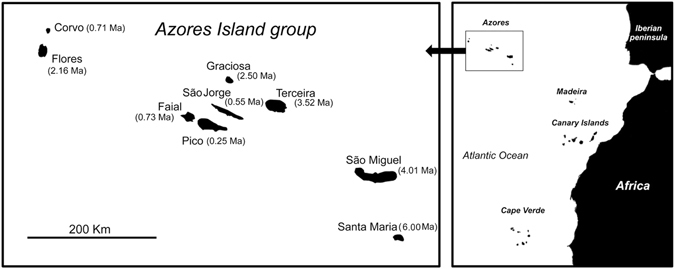
The Azorean archipelago. Maximum sub-areal dates for each island are in million of years (Ma)^29^. Maps were generated with Map data: Google, DigitalGlobe and modified using Adobe Illustrator CS3 13.0 and Adobe Photoshop CS 8.0 (Adobe Systems Incorporated).

We used a unique dataset on the well-studied arthropod community of the Azorean archipelago^6, 11, 27, 28^. The Azores (37° to 40° N; 25° to 31° W) is one of the world’s most isolated archipelagos, consisting of nine volcanic islands aligned on a WNW–ESE axis in the Atlantic Ocean (Fig. 2). The islands are geologically recent, with ages varying between 0.25 Ma for Pico and 6.0 Ma for Santa Maria^29^.

The diversity of the native Azorean arthropod fauna has been studied since 1998 with standardized methods and covering several spatial scales^20, 27^, thus providing one of the best studied oceanic island systems globally (BALA project, 1999–2012; **B**iodiversity of **A**rthropods from the Laurisilva of the **A**zores^27, 28^). The dataset consists of information on the location and species identity of all individuals collected and of the relative abundance of each species. We gathered the data with intensive standardized sampling techniques in the soil and canopy habitats of 99 transects (each 150 m long by 5 m wide) covering seven islands and 18 fragments of native forest. The sampling effort consisted of 30 pitfall traps, spaced 5 m apart, for epigean arthropods, and a maximum of 30 samples for canopy arthropods, consisting of 10 samples for each of three dominant plants in the transect^27, 28^. In a few transects only one or two plant species were available for canopy beating. We emphasize that all transects covered the same area (150 m by 5 m) and a similar sampling effort was applied to each transect. All individuals sampled (soil and canopy) for each site were pooled together. Therefore, we treated the increase in the number of transects as a surrogate for an increase in both area and sampling effort.

For the current study we restricted our analyses to herbivores and predatory arthropods (excluding Diptera and Hymenoptera; Araneae, Hemiptera, Coleoptera and Lepidoptera were the dominant groups in the dataset) since these are the most common ecological groups in the dataset (see Supplementary Material Table S1). All sampled species were classified as possessing either high or low dispersal ability by a trained taxonomist (PAVB)^30^. We categorized the dispersal ability of each species based on knowledge accumulated since 1994 on the biology of the species, from studies on pasture^31^, native forest^20, 27, 28^ and other major Azorean habitats^20^. Such information was included in a trait database that has already been used in previous publications^21, 32, 33^. For the 337 species we have analyzed we do not have quantitative data on their dispersal ability. Therefore, we categorized species into high and low dispersal classes based on their known ecological attributes and morphological characteristics, such as the presence of active wings for Coleopterans and Hemipterans, ballooning for spiders and descriptions of flying ability for endemics and general guides for the other species. To be considered as a good disperser, a species has to be able to disperse between fragments of native forest and surpass the current matrix of manmade pastures^20^. Traits related with dispersal ability were collected from an extensive literature search of ecological information, including manuscripts with the first descriptions of the species, first species records for the Azores, brief notes, and ecological studies among others. Information was also obtained from experts who have identified the specimens or from experts of a given taxonomic group when information for a particular species was not available. Most of the literature was retrieved from the taxonomical catalogue of the entomological bibliography for the Azores^34, 35^, with the addition of some recent documentation^36^. We list the species in Supplementary Material Table S1 and summarize in Table 1 the information for each island on the number of species and individuals for low and high dispersal ability. See, also, the Supplementary Material for a sensitivity analysis of the effects of possible misidentifications of a species’ dispersal ability on the evolution of the SADs as a function of scale.

### Adding transects

The explanation for the different pace of change of the SADs for high and low dispersal ability species (Fig. 1

**Table 1:** Number of species and individuals of arthropods in the categories of high and low dispersal ability, for the samples collected in each of the Azorean islands.

Island	N° Transects	High dispersal ability		Low dispersal ability	
		N° Individuals	N° Species	N° Individuals	N° Species
Faial	8	5450	59	1415	39
Flores	12	9845	83	2161	39
Pico	16	17127	93	4970	52
Santa Maria	4	4303	63	786	33
São Jorge	8	9043	84	2081	37
São Miguel	12	6923	101	2601	48
Terceira	39	23973	124	11850	57
Total	99	76664	235	25864	102

The data were collected from 1999 to 2012 along transects (each 150 m long by 5 m wide) in 18 fragments of native forest. The sampling effort was the same for all transects and consisted of 30 pitfall traps, spaced 5 m apart, for epigean arthropods, and a maximum of 30 samples for canopy arthropods, consisting of 10 samples for each of three dominant plants in the transect. Note that the results presented in the main text refer only to Pico Island and to Terceira Island. Results for the other islands can be found in Supplementary Figs 4–7.

) hinged on the spatial arrangement of the individuals, in particular, their proximity^22^. Thus, sample size increased by gathering information on adjacent points in the simulation matrix. Our data on arthropods were collected in transects and for each individual we assigned the spatial location of the centre of the transect in which it was found. In order to interpret the results based on the spatial distribution of the individuals we added transects based on their proximity^27, 30^. We devised two methods to add transects, one we called concentric, the other sequential. For the concentric method, we chose a transect and then identified the nearest one, then the second nearest one, and so on. For the sequential method, we chose a transect and identified the nearest one, then the nearest to the latter one, and so on. These two procedures were repeated starting from each transect, hence the number of possible transect sequences is equal to the number of transects. In practice the results obtained with these two methods were virtually identical, and we present only those for the concentric method.

### Binning method

To display SADs we considered the following binning scheme^24^. If *S*_*n*_ is the number of species with *n* individuals then bins are for counts *S*_1_, *S*_2_ to *S*_3_, *S*_4_ to *S*_7_, *et seq*. Other schemes^1, 37^ gave identical results.

### Moments

As moments measure different properties of a distribution, we used them to describe the scaling characteristics of the SADs. We used three different types of moments: raw, central or standardized. The easiest to calculate are the raw moments (or just moments). If we use the logarithmic transformed values of the number of individuals, *x*, then the raw moments, *M*_*n*_, are calculated using the formula

1 $${M}_{n}=\frac{1}{S}\sum _{j=1}^{S}{x}_{j}^{n},$$

where *n* is the order of the moment, and *S* is the total number of species; notice that the first moment is the mean. An alternative formula for the raw moments is

2 $${M}_{n}=\sum _{i=0}^{NB-1}{p}_{i}{x}_{i}^{n},$$

where *NB* is the total number of classes (number of bins in a histogram) and *p*_*i*_ the proportion of species in class *i*. The central moment of order *n*, *C*_*n*_, is defined as

3 $${C}_{n}=\frac{1}{S}\sum _{j=1}^{S}{({x}_{j}-\bar{x})}^{n},$$

where $\bar{x}$ is the mean (the first raw moment); notice that the second central moment is the variance. Finally, the standardized moment of order *n*, *T*_*n*_, is defined as

4 $${T}_{n}=\frac{1}{S}\sum _{j=1}^{S}{(\frac{{x}_{j}-\bar{x}}{{\sigma }_{x}})}^{n},$$

where *σ*_*x*_ is the standard deviation. The third standard moment is often used to describe the skewness of the distribution^26^

5 $$Skewness=\frac{1}{S}\sum _{j=1}^{s}{(\frac{{x}_{j}-\bar{x}}{{\sigma }_{x}})}^{3}.$$

Positive skewness indicates a distribution leaning to the left with a pronounced right tail, negative skewness reveals the opposite, and skewness equal to zero reveals a symmetric distribution^26^. For instance, according to the results obtained with simulations shown in Fig. 1a,b, we expect the skewness of the SAD of high dispersal species to be larger than for the low dispersal ability species when examining identically sized samples. This is in fact what we observe when we calculated the skewness from these distributions, except for very large samples when the distributions are again similar (Fig. 1c).

### Calculating the skewness and forecasting the species abundance distributions

In all calculations we used logarithms of base 2 of the number of individuals^8^. For each island and for all possible sequences of transects we estimated the skewness, using equation (5), as a function of the number of transects. Finally, we summarized this information by calculating the mean and the standard deviation of the skewness for all transects and their sequences.

To forecast the SADs for larger sample sizes we used a method based on the raw moments^5^. The basic idea consists of extrapolating the moments calculated from equation (1) to larger areas and then using the discrete scaled Tchebichef polynomials and moments^19^ to estimate the SAD. The application of the Tchebichef moments leads to the perfect reconstruction of a distribution when we use equation (2) to estimate the moments (see Supplementary Information). However, this is only an approximation because once the number of individuals is log-transformed we stop dealing with the real transformed values of the number of individuals and instead consider the discrete number of values attributed to each bin class. We could calculate the moments based on equation (2), but the problem is that they would stop showing the clear trend as a function of the number of transects exhibited by the moments calculated using equation (1). The extrapolation of the values of the moments estimated from equation (1) to larger areas leads to numerical instabilities of the Tchebichef moments of higher orders (see ref. 5), thus we cannot use all the available moments and need to consider only the lower ordered moments (see also ref. 5). For estimating the number of moments for reconstructing a distribution, we tested the number of moments calculated from equation (1) that gave the best reconstruction of the distribution to the largest area available, with the best reconstruction being determined by the number of moments that minimizes the sum of the square of the difference of the histogram of the real data and that obtained with the Tchebichef method.

The procedure is:

    (i) for each sample size, estimate the moments up to several orders (for instance, 10) using equation (1);
   (ii) plot each moment as a function of the sample size in a double logarithmic plot;
  (iii) identify a (scaling) region where the moments fall into an approximate straight line;
 (iv) obtain the parameters of the regression line going through the points in the scaling region;
  (v) using these parameters, estimate the moments for the largest number of transects, and with the known total number of species and individuals, determine which number of moments gives the best fit reconstruction of the empirical distribution using the scaled Tchebichef polynomials and moments;
 (vi) use these parameters to estimate the value of the required moments for larger sample sizes;
 (vii) simultaneously, estimate the scaling properties of the number of species and of the number of individuals of the most abundant species (the number of species usually scales as a power law^4^), as does the number of individuals of the most abundant species according to our experience;
(viii) estimate the number of species and the number of individuals of the most abundant species to the desired larger sample size (the latter will be used to estimate the number of abundance classes of the forecasted distribution); and
  (ix) use the extrapolated moments to calculate the scaled Tchebichef polynomials and moments to obtain the shape of the probability density function and, finally, multiply these values by the forecasted number of species to obtain the SAD (see also the Supplementary Information).

A few caveats to the above procedure are in order. In step (iii) we recommended a straight line to fit the moments, i.e., a power law function. This is not strictly necessary and we can easily assume other functions. We used a power law here for simplicity because: i) we are dealing with a small number of transects, ii) previous work^5^ showed it to be a reasonable approximation, and iii) for the range of number of transects available it provides a good fit. To further test the power law assumption, we first estimated its parameters from only half the data, then extrapolated the moments and obtained from these the distributions using the scaled Tchebichef polynomials and moments, and, finally, we compared the forecasted distributions to the empirical one. Although we are typically dealing with a small scaling region, the extrapolated distributions follow the empirical distributions (see Supplementary Fig. S3). We reiterate that step (v) is important because higher moments are very sensitive to the approximations made, which may lead to numerical instabilities^5^ that affect the projected SADs. In fact, although it may be useful to use a large number of moments (for example, 10) to determine the scaling region, in most cases the total number of moments used to forecast the distribution will be smaller. Notice that when numerical instabilities are not present high moments add only minor features to the distribution and do not affect significantly its general shape^5^ (and see Supplementary Fig. S2). Finally, only steps (v) and (ix) require more elaborate calculations, and for these we give further explanations in the Supplementary Information, including an example of how to calculate the scaled Tchebichef moments and polynomials and the R^38^ function used to perform these analyses.

Notice that due to the way we add transects, moment values were calculated by accumulating abundance data, thereby violating the assumption of independence in the regression analysis. Because of this, we re-ran our analysis with generalized least squares models (function gls in R^38^ package “nlme”) by (1) including a the first-order autoregressive structure with respect to the number of transect using the option “corAR1”) and (2) specifying a variance structure using the option “varPower”. These methods allow for model errors to be autocorrelated, accounting therefore for the lack of independence between the moment values, and they permit us to model non-constant variance when the variance increases or decreases with the mean of the response. However, such models requires a substantial number of data points to reach convergence^39^, while for most islands we have a very small number of transects (Table 1). Therefore, we were only able to apply the above procedure to the data for Terceira and Pico Islands, and for the data of Pico island without taking into account heteroscedasticity. Nevertheless, the results were unchanged in comparison to simple linear least squares regression. Due to space limitations, we present here only the results for the two islands with the largest number of transects, Pico and Terceira; for the other islands see Supplementary Figs 4–7).

All statistical analyses were implemented within the R programming environment^38^. Because we aim to compare the results of the extrapolation with those of real data, and because we do not foresee that the present data set will increase dramatically in the immediate future, we extrapolate the distributions only up to two and (optimistically) to four times the number of transects.

## Results

**Figure 3:**
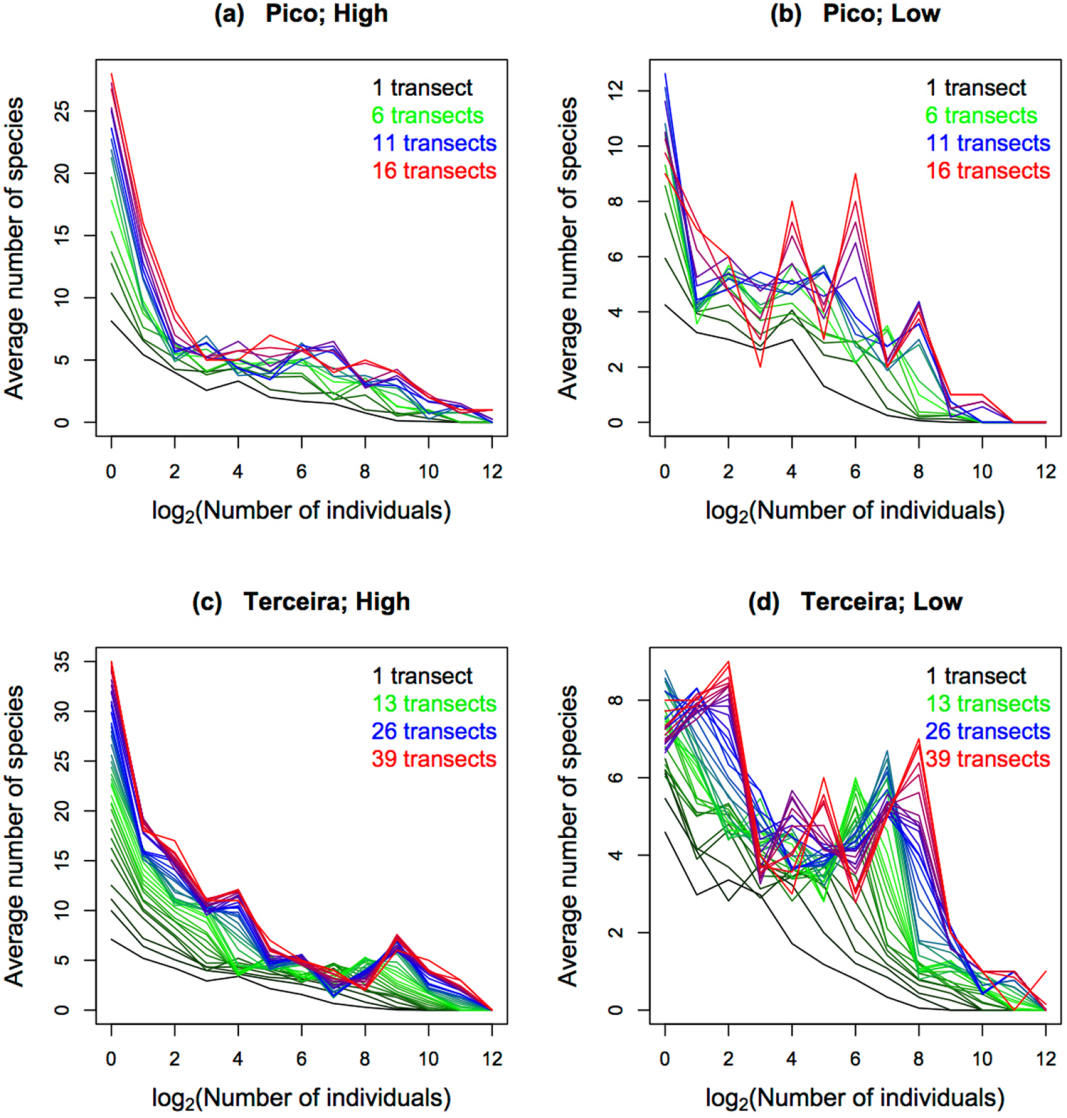
SADs of Pico and Terceira islands for arthropod species groups with high and low dispersal abilities. Each curve corresponds to the average of all possible SAD curves obtained by using the concentric procedure as explained in the Methods. The x-axis corresponds to classes of the logarithm of base 2 of the number of individuals as follows: 1 individual, 2 to 3 individuals, 4 to 7 individuals, *et seq*. In order to better illustrate the evolution of the shapes of the distributions, the curves have a gradient of colours going from black (the smallest number of transects), through green and blue, to red (the largest number of transects). In all cases we can observe the development of humps when the number of transects increases. However, while for species with high dispersal ability the number of singletons keeps increasing with the number of transects, for species with low dispersal the number of singletons decreases for the largest number of transects; in fact, for Terceira, when a large number of transects is added, the absolute maximum no longer occurs for the singleton abundance class, but for some intermediate classes.

In agreement with the simulations (Fig. 1), for small sample sizes the SADs of Pico and Terceira islands are almost monotonically decreasing functions, but when sample size increases, the shapes of the distributions differ considerably between the high and low dispersal ability groups (Fig. 3 and see Supplementary Fig. S4 for the SADs of all islands). For high dispersal ability species (Fig. 3a,c), when the number of transects increases the number of singletons keeps increasing, although some peaks start appearing for intermediate abundance classes. For species of low dispersal ability and in the case of Pico Island (Fig. 3b), an increase in sample size leads to an increasing number of singletons and to the presence of several maxima for intermediate classes. For Terceira Island (Fig. 3d), above a certain number of transects the absolute maximum shifts to the second abundance class. We ascribe this more obvious transition for Terceira because it has a much larger number of transects than does Pico. In general, for the low dispersal ability curves we observe a larger proportion of species among the intermediate abundance classes and a proportionally smaller number of singleton species.

**Figure 4:**
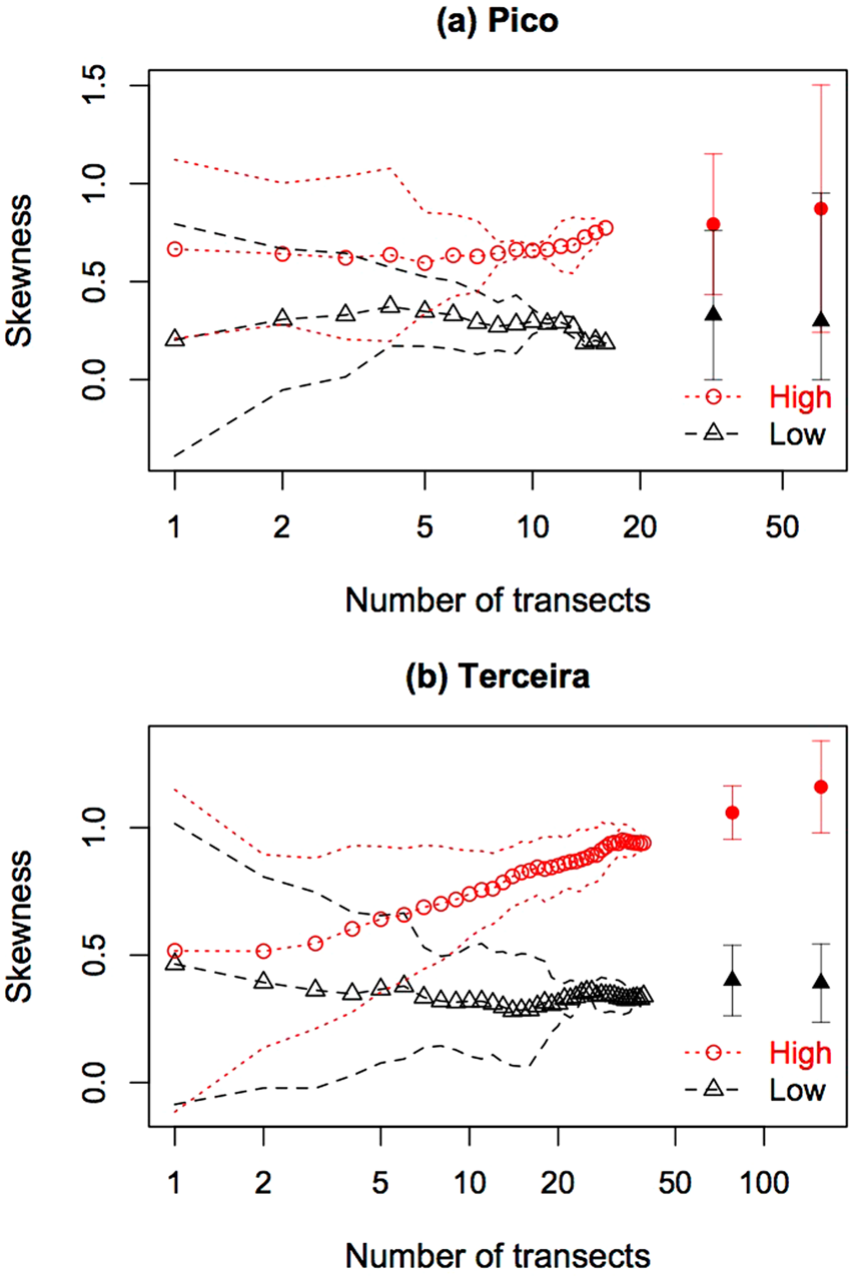
Trend of the skewness (third standardized moment) of the SAD as a function of the number of transects. The symbols in red are for high- and in black for low- dispersal ability arthropod species groups of Terceira and Pico islands. The circles and triangles correspond to the mean values of the skewness calculated from the SAD obtained from all possible addition of transects using the concentric procedure as explained in the Methods. The dot and dashed lines correspond to 2 standard deviation confidence intervals. The two rightmost bold dots are the skewness estimated from extrapolated SADs and the arrow bars correspond to two standard deviations.

Skewness is always larger for the high than for the low dispersal ability curves (Fig. 4 and Supplementary Fig. S5). In particular, notice that the skewness of low dispersal ability species decreases with increasing number of transects, while that of high dispersal ability species keeps increasing. However, a closer look at the Terceira results suggest that the skewness of the distributions reduces its pace of change with the number of transects (Fig. 4b).

**Figure 5:**
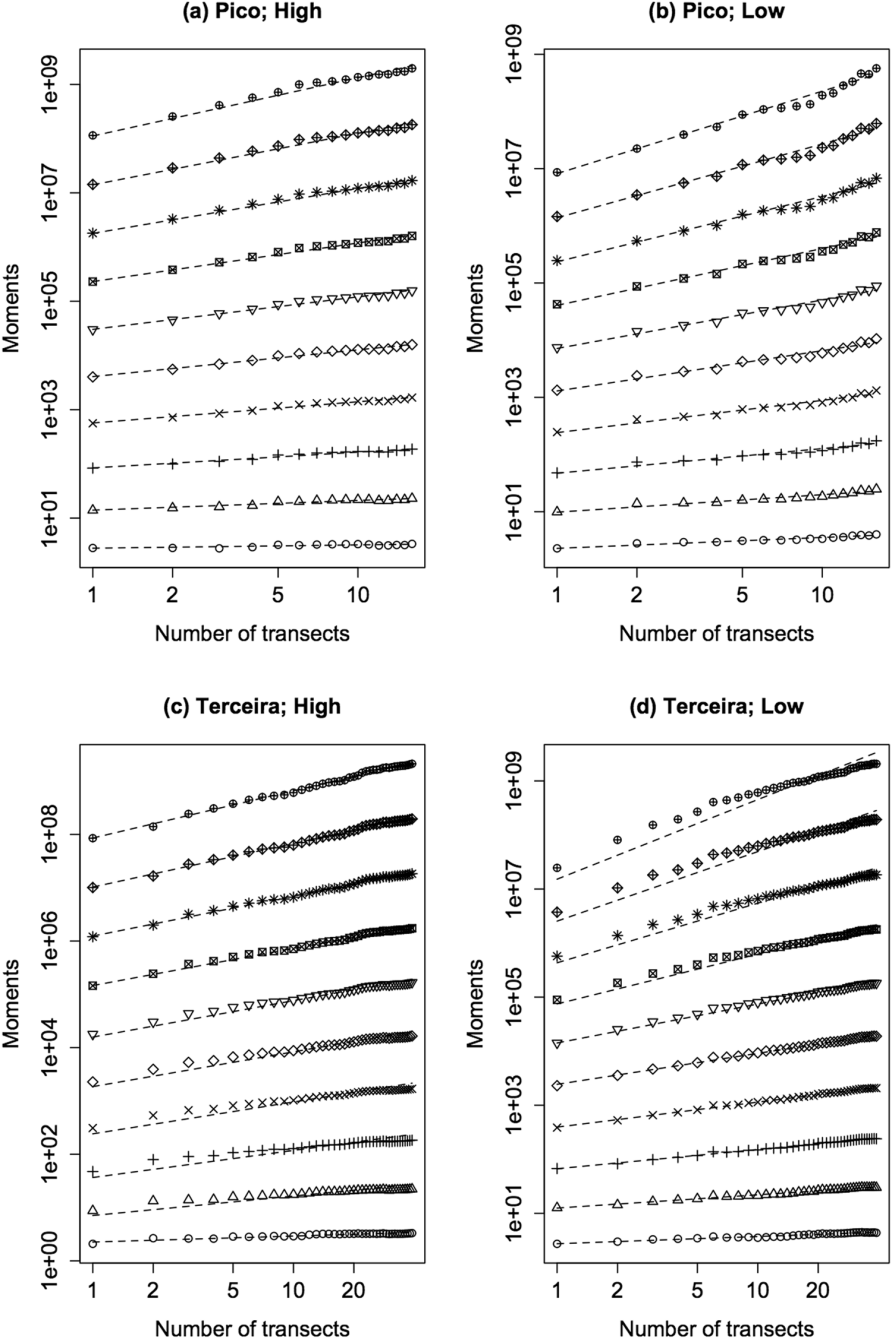
Double logarithmic plots of the first 10 integer moments as a function of the number of transects. Plots a and c correspond to high dispersal ability and panels b and d to low dispersal ability arthropod species of Pico and Terceira islands. In both cases the sequence of added transects was that obtained by using the concentric procedure, as explained in Methods. The order of the moments increases from the bottom to the top lines. Notice that these plots correspond to one sequence of added transects. The dashed lines were obtained from linear regression of the logarithmic transformed value of the moments.

**Figure 6:**
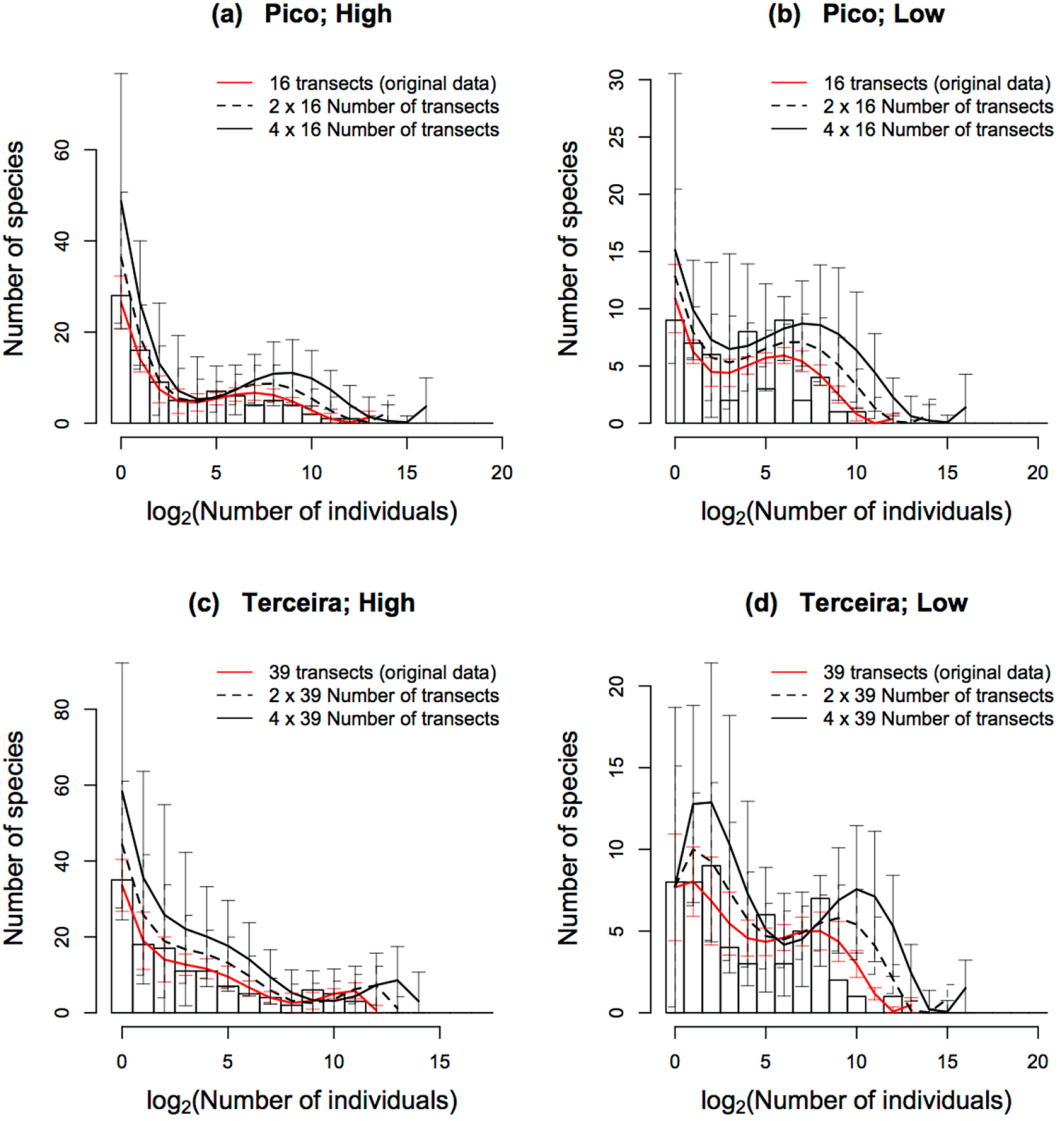
Histograms and extrapolated distributions for arthropod SADs of all transects combined for the Pico and Terceira islands. The red curve is the distribution estimated from all transects using the scaled Tchebichef moments and polynomials. The black and full dashed lines are the forecasted distributions for two and four times the original number of transects and they correspond to the mean value of the distributions obtained for all possible additions of transect sequences; these distributions were used to obtain ±2 standard deviations shown by the error bars. We used up to the 4^th^ order moment for both the high and low dispersal ability species of Pico Island and to 3^rd^ and 5^th^ order moments for the high and low dispersal ability species, respectively, of Terceira Island. The x-axis corresponds to classes of the logarithm of the number of individuals as follows: 1 individual, 2 to 3 individuals, 4 to 7 individuals, *et seq*.

We now use the raw moments, equation (1), to forecast the SADs for larger samples. These moments follow almost straight lines when plotted in double logarithmic axes (Fig. 5), a result we also obtained in our previous analyses of tropical tree species relative abundance in Barro Colorado Island and to which we will return in the Discussion. We take advantage of the linearity observed in the log-log plots to fit the logarithm of the moments using linear regressions (see dashed lines in Fig. 5). The residuals show an oscillatory behaviour (Supplementary Figs S6 and S7), therefore revealing autocorrelation, although the amplitude of the oscillations, that is, of the absolute value of the residuals, is very small. By applying the procedure outlined in the Methods to all possible sequences of transects, and then averaging the values of the distributions of each sequence, we arrived at the extrapolated distributions (Fig. 6, and see Supplementary Fig. S8 for all islands). For the SADs of high dispersal ability species (Fig. 6a,c), the forecasted distributions have a larger number of singletons, but in the case of Pico the development of a second maximum for intermediate classes is clearly discernible, with species shifting to higher abundance classes as the sample size increases (Fig. 6a). For low dispersal ability species the developing trend is more interesting. For Pico (Fig. 6b) we forecast simultaneously an increase in the number of singletons and a hump for intermediate classes. For Terceira (Fig. 6d) we forecast two maxima, but none occurring for the singletons class. Indeed, the average distributions show a slightly smaller number of singletons when sample size increases. Again, the difference between Pico and Terceira islands, with the shape of the latter being more different from a monotonically decreasing function, is likely to be a consequence of the larger sample size for Terceira.

In summary (see also Supplementary Fig. S8), and within the allowed ranges of increase of the number of transects, for high dispersal ability species we forecast a higher number of singletons and the development of a more pronounced hump for intermediate abundance classes. For the distributions of low dispersal ability species the trend is similar, but the increase in the hump for intermediate abundant species is more pronounced; in some cases (Terceira and São Miguel, see Fig. 3 and Supplementary Fig. S4 respectively) the maximum among the rarest species occurs not for the singletons’ class but for the adjacent abundance class.

## Discussion

Some macroecological phenomena only reveal their patterns when analysed at different scales, for example, the species–area relationship^13, 40–42^. We posit that the scaling of relative abundance of species is important and should also be studied as a function of sample size, or area, depending on the circumstances. Accordingly, we developed quantitative tools to describe the patterns associated with the scaling properties of the SADs based on their moments. We emphasize that our approach can be seen as an extension of studies on species richness across spatial scales. However, unlike species richness, the characterization of species abundance across scales requires the description of a distribution, achieved herein by using their moments to generate a family of curves (i.e. those of the raw moments across scales), instead of one single curve, the SAR. Here we used data on low-diversity island arthropod communities and, remarkably, the double logarithmic plots of the moments as a function of sample size were almost straight lines as previously observed (above some scales) for the hyper-diverse tropical tree species communities sampled on Barro Colorado Island, Panama^5^.

Based on predictions from simulations^22^, we combined our studies on the scaling properties of SADs with the role of dispersal. Dispersal plays a major role in species assembly and, in particular, how species are spatially arranged (e.g. more or less clustered), determining the spatial turnover of species, hence how new species are found as a function of increasing sample size or area^43^. Moreover, simulations showed that dispersal ability affects the way SADs change as a function of sample size: SADs of low dispersal ability species develop a maximum for intermediate abundance classes earlier, i.e., for smaller sample sizes, than the SADs of high dispersal ability species, with the latter retaining a larger fraction of singleton species when sample size increases^22^. Here, we showed that these trends are also observed in the arthropod communities of the Azorean archipelago. However, because the more general point of our analyses is comparing how distributions change as a function of sample size, we shifted our attention from attempting a detailed description of the distributions at one particular scale and, instead, concentrated on characterizing how the distributions change across scales, for which we use their moments. Of course, we should expect that all the moments, such as the mean or the variance, change when the underlying distributions change. ﻿Nevertheless﻿, one moment, the skewness, is particularly useful for describing the changes in the symmetry of the distributions when sample size increases. As we discussed previously, the change in the symmetry of the SADs can be interpreted in terms of the spatial distributions of the species and these spatial arrangements can be explained by their dispersal ability. Thus, we suggest that comparing the evolution of the skewness as a function of sample size for different communities (or groups of species) can be a first approximation to ascertaining the dispersal ability of the respective species.

Our work depended heavily on the ability to deconstruct the SAD into that of high and low dispersal ability species. Such a separation is easily done in computer simulations, where one can specify the dispersal attributes of every species. In reality, however, dispersal ability forms a continuum, not a dichotomy. As our study included many contrasting groups of arthropods, it was impossible to obtain a continuous metric for all. Yet, remarkably, having used expert knowledge to classify species based on dispersal ability, we found that their SADs changed in very different ways as a function of sample size, following the trends predicted by previous simulations^22^.

Although our results indicate that dispersal ability seems to be a determining factor of the scaling properties of the species abundance distribution of the﻿ ﻿arthropod community of the Azores, it would be incorrect to attribute the differences in the SADs solely to dispersal ability^21, 44^. Dispersal ability measures one of the attributes of the species that affects their spatial distributions. Other aspects, such as the mosaic of habitats and species affinities to particular micro-habitats, competition and biotic interactions, also determine the spatial distribution of species and, ultimately, the scaling properties of the SADs. In fact, those processes may help explain the presence of more than two modes in our data^11, 45^. As we noted in the methods section, transect proximity is also likely to play an important role. Indeed, the distance among transects can be a surrogate for the homogeneity of the regions where transects are located, or even whether they belong, or not, to the same contiguous patch of native forest. For instance, shorter distances may lead to a more similar fauna, hence a smaller number of singleton species. In this respect, it is interesting to note that the transects in São Miguel Island are among those with the shortest mean and maximum distances (mean = 3350 m; max = 5500 m), which may explain why we observe a maximum for intermediate classes earlier than in other islands (Supplementary Fig. S4).

An important feature of the simulations (Fig. 1) is that the SADs of high and low dispersal ability species are again similar for large sample sizes. We do not observe such a transition in our data for the sample sizes collected so far, or in the projections of the SADs for larger sample sizes. Obviously the area covered by transects corresponds to a very small fraction of the total area of an island, and the same is true for the number of individuals. Therefore, if the trend obtained with the simulations for large sample sizes corresponds to a real phenomenon, our results indicate that more data are needed in order to observe the full range of the scaling behaviour of the SAD. Collecting such data, however, is a time consuming and expensive endeavour and may, in some situations, have a measurable negative effect on population sizes or species richness. This is an example where the development of methods attempting to forecast SADs for large areas may ameliorate the problems associated with the empirical collection of data. Here we used a method based on scaled Tchebichef moments and polynomials^5^, but being a recent method, only tested in a few datasets, care is needed when applying it. Foremost, we do not know enough about the behaviour of the moments to have enough confidence to project the distributions for very large sample sizes. For instance, in the same way we observe a transition in species accumulation curves from very small areas, where they are not well approximated by power laws, to intermediate areas, where power laws provide a good approximation^4, 24, 46^, it is very likely that the moments of SADs exhibit several transitions as a function of area. In general, the caveats that apply when dealing with species accumulation curves, such as the choice of a relatively homogenous area, also apply here^4^. To address these issues we require more work on the analytic techniques to extrapolate the SAD and a better understanding from empirical and theoretical perspectives of how the transitions in the scaling properties of the moments occur. We plan to address these concerns in future work.

Finally, we emphasize that our aims are not only theoretical but also practical. Indeed, we endorse Matthews & Whittaker’s^7^ assessment that despite the large volume of work on the theoretical aspects of the SAD, its practical uses have not been fully explored, in particular in the field of biodiversity management and conservation. We suggest two further research avenues concerning SADs. First, there is a need to describe theoretically how the raw moments change as a function of sample size, assuming theoretical distributions that provide good fits at different spatial scales; for instance, the logseries often provides a good fit for small spatial scales while the lognormal distribution, or other distributions with similar shapes, provide a good fit when sample size increases^3^. Second, we suggest that it is important to explore methods developed in other areas to better extrapolate the raw moments and reconstruct the SADs. For instance, in our work, besides Tchebichef moments, we have tried Legendre moments (with poorer results), but other methods exist that are constantly being improved or developed^47, 48^. We hope that this study, by further exploring a pattern and a methodological approach, will stimulate practical applications of SADs.

## Electronic supplementary material

Supplementary Information
