# Supplementary material for: Dispersal ability determines the scaling properties of species abundance distributions: a case study using arthropods from the Azores
Source: Sci Rep. 2017 Jun 20;7:3899. doi: 10.1038/s41598-017-04126-5 (PMC5478659; doi:10.1038/s41598-017-04126-5)
Supplement: Supplementary file 1 — Supplementary Information [file 41598_2017_4126_MOESM1_ESM.pdf]

Dispersal ability determines the scaling properties of species abundance distributions: a case study using arthropods from the Azores

Luís Borda-de-Água, Robert J. Whittaker, Pedro Cardoso, François Rigal, Ana M. C. Santos, Isabel R. Amorim, Aris Parmakelis, Kostas A. Triantis, Henrique M. Pereira & Paulo A. V. Borges

SUPPORTING INFORMATION

R function to perform the calculation of the scaled Tchebichef moments and polynomials

Here we explain how to reconstruct a frequency distribution, $f(x)$, from knowledge of its raw moments using scaled Tchebichef moments and polynomials[19]. In the context of this paper it means reconstructing the species abundance distribution from knowledge of its raw moments. In addition, we provide a function in *R* to calculate the scaled Tchebichef moments and polynomials and exemplifies the application of the method.

The idea of reconstructing a distribution, $f(x)$, from its moments using scaled Tchebichef moments and polynomials can be explained as follows. If you know the moments then you can calculate the scaled Tchebichef moments, T_n, which are then the weights of the scaled Tchebichef polynomials, $\tilde{t}_n(x)$, through the formula

$$f(x) = \sum_{n=0}^{N} T_n \tilde{t}_n(x), \quad \text{(S1)}$$

where the maximum value of N coincides with the number of bins. By giving different weights to each polynomial we can obtain through the above sum the shape of the distribution from which the original moments were calculated.

The basic ingredients to reconstruct the frequency distribution are: knowledge of the raw moments, M_n, the number of required bins, N (in practice, we need to estimate the abundance of the most abundant species) and the total number of data points, S (that is, the number of species). Assuming that these are known, we can calculate the scaled Tchebichef polynomials, $\tilde{t}_n(x)$, using the following recurrence formulas

$$\tilde{t}_0(x) = 1$$

$$\tilde{t}_1(x) = (2x + 1 - N) / N$$

$$\tilde{t}_n(x) = \frac{(2n-1)\tilde{t}_1(x)\tilde{t}_{n-1}(x) - (n-1)\left(1 - \frac{(n-1)^2}{N^2}\right)\tilde{t}_{n-2}(x)}{n} \quad n=2,3,\ldots,N\text{-}1. \quad \text{(S2)}$$

We show in Fig. S1 the scaled Tchebichef polynomials from order n = 0 to order n = 5. These are the polynomials that are going to be added weighed by the respective scaled Tchebichef moments, T_n, according to Eq. S1.

Finally, the scaled Tchebichef moment of order n, T_n, with $0 \le n < N$, is given by

$$T_n = \frac{1}{N^n \tilde{\rho}(n,N)} \sum_{k=0}^{n} C_k(n,N) \sum_{i=0}^{k} s_k^{(i)} M_i \, , \quad \text{(S3)}$$

where M_i is the raw moment of order i, which we assumed to be known, $s_k^{(i)}$ are the Stirling numbers of the first kind, $C_k(n,N)$ is

$$C_k(n,N) = (-1)^{n-k} \frac{n!}{k!} \binom{N-1-k}{n-k} \binom{n+k}{n},$$

and $\tilde{\rho}(n,N)$ is

$$\tilde{\rho}(n,N) = \frac{N\left(1-\frac{1}{N^2}\right)\left(1-\frac{2^2}{N^2}\right)\ldots\left(1-\frac{n^2}{N^2}\right)}{2n-1};\ n=0,1,\ldots,N\text{-}1.$$

Notice that the information on the distribution that we want to reconstruct is provided through the raw moments, M_i, in eq. S3.

We exemplify the reconstruction of a frequency distribution using the *R* function called *figure.S2*. This is a demonstration function of the function *tcheb.mom.pol* which is the one that implements the above expressions to calculate the scaled Tchebichef moments and polynomials. We hope that by providing an example of how to use the *tcheb.mom.pol* function, this function can be used in a wide variety of situations with some adaptations to the problem at hand.

The function *figure.S2* assumes that the package "untb" has been installed. If it has not, then you should run the following instruction before using the function *figure.S2*

install.packages("untb")

(assuming that you have access to the internet).

We suggest you copy and paste function *figure.S2* into the *R* console. If you then type the instruction

figure.S2()

you should obtain a plot such as Fig. S2.

This is a brief explanation of the function *figure.S2*. It starts by defining the distribution through the instructions

```
mids <- c(0,1,2,3,4,5,6,7,8, 9,10,11)
counts <- c(27,11,20,30,29,38,30,21,20,5,4,1)
```

(Notice that this is rather artificial since we know beforehand the distribution, the point here is solely to see how well the method performs and, simultaneously, provide an example on how to call the function *tcheb.mom.pol,* which is the one that really matters.)

Function *figure.S2* calculates now the raw moments, through the instructions

```
mom <- rep(0,no.m)
mom[1] <- 1
for(i in 1:(no.m-1))
  mom[i+1] <- sum(mids^i*counts)/S
```

In a practical example the moments would probably be the input of the function, which are assumed to have been calculated somewhere else. For instance, they could be values forecasted from regressions, as we did in the main text.

The calculation of the scaled Tchebichef moments and polynomials is then performed by calling the function *tcheb.mom.pol*

```
fx1 <- tcheb.mom.pol(no.m,no.m,mom,S)
fx2 <- tcheb.mom.pol(   6,no.m,mom,S)
```

Here we called the function twice because we want to exemplify the reconstruction of the distribution using the maximum allowed number of moments (twelve) and a

smaller number of moments (six) to illustrate how an increase in the number of polynomials increases the fitting of the curve to the original histogram. In a typical situation it would be probably called only once.

The function *tcheb.mom.pol* has the following structure

tcheb.mom.pol <- function(no.m,no.bins,mom,S)

and the meaning of the input variables is:

- *no.m* is the number of raw moments and, therefore the number of scaled Tchebichef moments and polynomials,
- *no.bins* is the number of bins,
- *mom* is a vector containing the raw moments, and
- *S* is the total number of points to obtain the frequency distribution.

The number of moments, *no.m*, should be smaller or equal to the number of bins, *no.bins* (see eq. S1). The function "tcheb.mom.poly" calls the functions "Ck.f", "ro.f" and "stirling.1st.ord" in order to implement equations S1 to S3.

The remainder of function *figure.S2* serves only to plot the histogram of the original distribution and the curves obtained with *tcheb.mom.pol*.

FIGURES

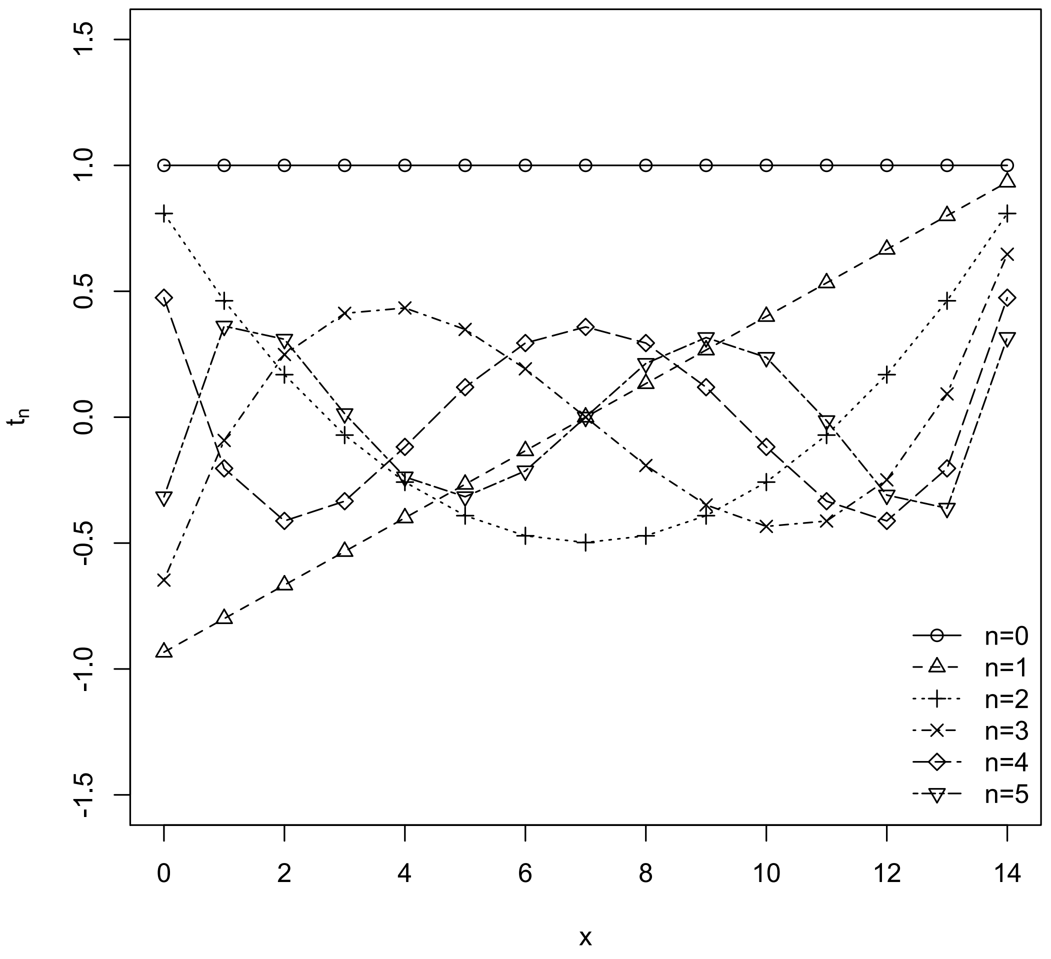

Figure S1. The shapes of the scaled Tchebichef polynomials from order 0 (a horizontal line equal to 1) to order 5.

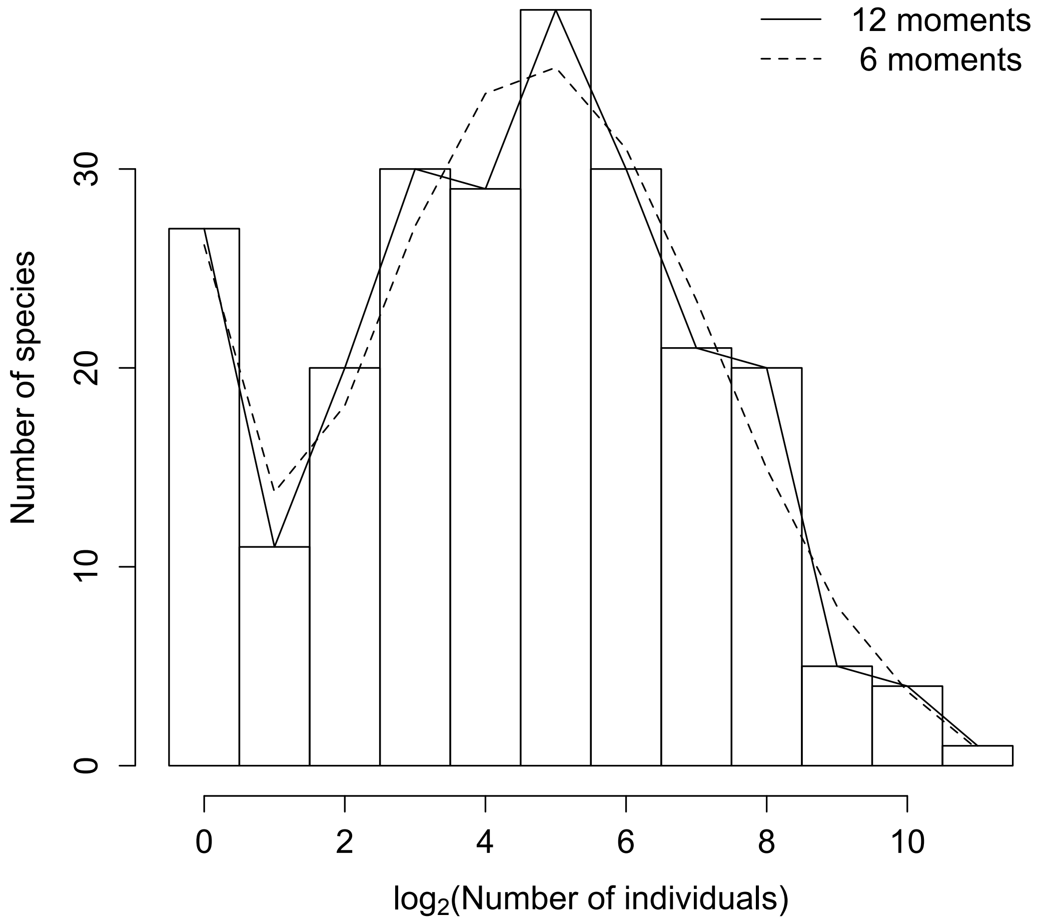

Figure S2. Figure obtained with the *R* function *figure.S2*. The original distribution was assumed known, and from these we calculated the raw moments. We then reconstruct the original distribution using the information contained in the raw moments by using scaled Tchebichef moments and polynomials up to order 5 and 11, to which corresponds 6 and 12 moments, respectively. Notice that 6 moments (from order 0 to 5) already provide a reasonable fit to the empirical distribution; the use of 12 moments leads to a perfect fit.

###

```
figure.S2 <- function(){

# no.m = n means that the order of the moments are 0,1,2...(n-1)
# number of bins = no.m

  library("untb") # to calculate Stirling numbers of 1st order

# if the library "untb" has not been installed, then before using this
# instruction type (assuming that you have access to the internet):
#    install.packages("untb")

   mids <- c(0,1,2,3,4,5,6,7,8, 9,10,11)
   counts <- c(27,11,20,30,29,38,30,21,20,5,4,1)

   no.m <-length(mids)
   S <- sum(counts)

   mom <- rep(0,no.m)
   mom[1] <- 1
   for(i in 1:(no.m-1))
     mom[i+1] <- sum(mids^i*counts)/S

   fx1 <- tcheb.mom.pol(no.m,no.m,mom,S)
   fx2 <- tcheb.mom.pol(   6,no.m,mom,S)
```

```
  br <- seq(-0.5, no.m - .5, 1)
  ymax <- max(fx1, counts)
  dat <- rep(mids[1],counts[1])
  for(i in 2:no.m) dat <- c(dat,rep(mids[i],counts[i]))

  hist(dat, breaks=br, main="", ylim=c(0,ymax), ylab="Number of species",
      xlab=substitute(paste(log[2],"(Number of individuals)", sep="")),
      cex.lab=1.25, cex.axis=1.25)

  x <- 0:(no.m-1)
  lines(x, fx1, lty=1)
  lines(x, fx2, lty=2)

  legend("topright",c("12 moments"," 6 moments"), lty=c(1,2), bt="n", cex=1.25)
}

###################################################

tcheb.mom.pol <- function(no.m,no.bins,mom,S){

# calculate the discrete Chebyshev polynomials

  N <- no.bins

  tp <- matrix(0,ncol=N, nrow=no.m)
```

```
  x <- 0:(N-1)

  tp[1,] <- 1
  tp[2,x+1] <- (2*x + 1 - N)/N

  for(p in 2:(no.m-1))
    tp[p+1,x+1] <- ((2*p-1)*tp[2,x+1]*tp[p,x+1] - (p-1)*(1-(p-1)^2/N^2)*tp[p-
1,x+1])/p

# calculate the discrete Chebyshev moments, Tp

  if(no.m > N) print("ATTENTION no.m > N")

  Tp <- rep(0,no.m)

  # first calculate ro (saves time)
  ro <- ro.f(no.m, N)

  for(p in 0:(no.m-1)){

    bet <- N^p
    Ap <- 1/bet/ro[p+1]

    # now calculate Ck
```

```
    Ck <- Ck.f(p,N)

    # now calculate SM = sum(sik * moments)

    SM <- rep(0,p+1)
    for(i in 0:p)
      SM[i+1] <- sum(stirling.1st.ord(i) * mom[1:(i+1)])

    # finally Tp

    Tp[p+1] = Ap * sum(Ck * SM)

  }

  fx <- rep(0,N)
  for(i in 1:N)
    fx[i] <- sum(Tp*tp[,i])

  return(fx*S)
}

##################################################

Ck.f <- function(n,N){
```

```
  Ck <- rep(0,n+1)
  k <- 0:n

  Ck <- (-1)^(n-k) * factorial(N-1-k) * factorial(n+k)
/(factorial(k))^2 / factorial(n-k) / factorial(N-1-n)
}

##################################################

ro.f <- function(n,N){

  ro <- rep(0,n)

  for(p in 0:(n-1)){
    i <- 0:p
    ro[p+1] <- N * prod( (1-i^2/N^2) ) / (2*p + 1)
  }

  return(ro)
}

##################################################

stirling.1st.ord <- function(k){
```

```
# Attention it requires the library untb


  if(k==0) S <- 1
  else{
    pm <- rep(0,k)
    for(j in 1:k) pm[j] <- (-1)^(k-j)
    S <- exp(logS1[k,1:k])*pm


    S <- c(0,S)
  }
  return(S)
}

##################################################
```

Testing the procedure using only half the data

Here we use half the data, ie, half of the transects, to test whether our procedure, assuming a power law behavior to the moments, provides a good fit between the extrapolated distribution and the empirically obtained distribution for all the transects of a given island. A similar procedure was adopted in ref. 5; see their Figure 3. However, compared to the data used in ref. 5 on tropical tree species in a contiguous 50ha plot in Barro Colorado Island, Panama, the problem we face with the present dataset is that we have a small number of transects for each island (with the exception of Terceira). Therefore, half that number provides a very small scaling region from which to estimate the slope and intercept of the perceived power law relationship; in fact, for Santa Maria island we do not attempt to test the method because the total number of transects is only four. For Terceira and Pico islands we also show the forecasted distribution based only one one quarter of the transects. Still, the forecasted distributions provided a reasonable estimate of the species relative abundance of the entire data set, Figure S3.

FIGURES

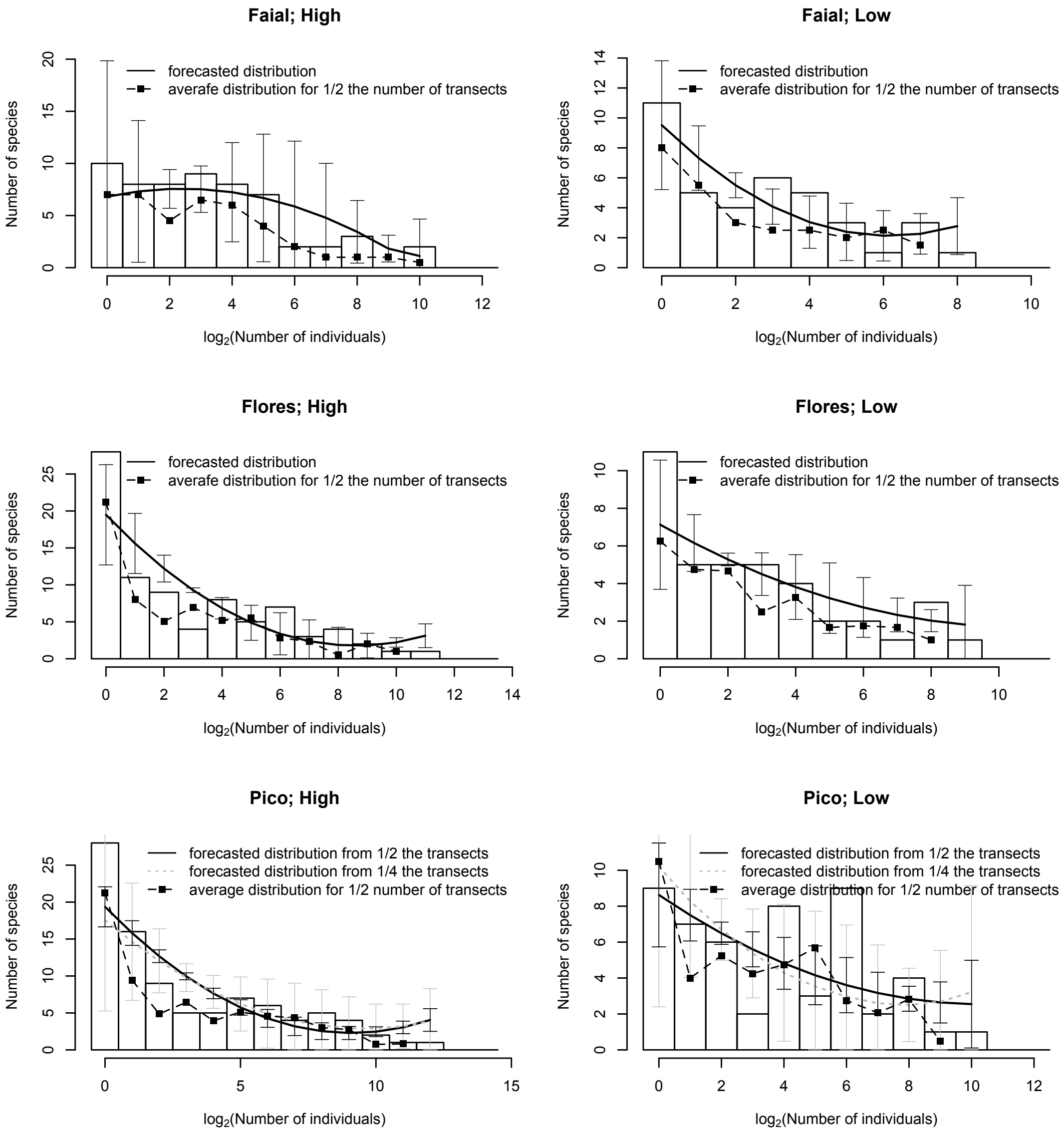

Faial; High
forecasted distribution
averafe distribution for 1/2 the number of transects
Number of species
log2(Number of individuals)
Faial; Low
forecasted distribution
averafe distribution for 1/2 the number of transects
Number of species
log2(Number of individuals)
Flores; High
forecasted distribution
averafe distribution for 1/2 the number of transects
Number of species
log2(Number of individuals)
Flores; Low
forecasted distribution
averafe distribution for 1/2 the number of transects
Number of species
log2(Number of individuals)
Pico; High
forecasted distribution from 1/2 the transects
forecasted distribution from 1/4 the transects
average distribution for 1/2 number of transects
Number of species
log2(Number of individuals)
Pico; Low
forecasted distribution from 1/2 the transects
forecasted distribution from 1/4 the transects
average distribution for 1/2 number of transects
Number of species
log2(Number of individuals)

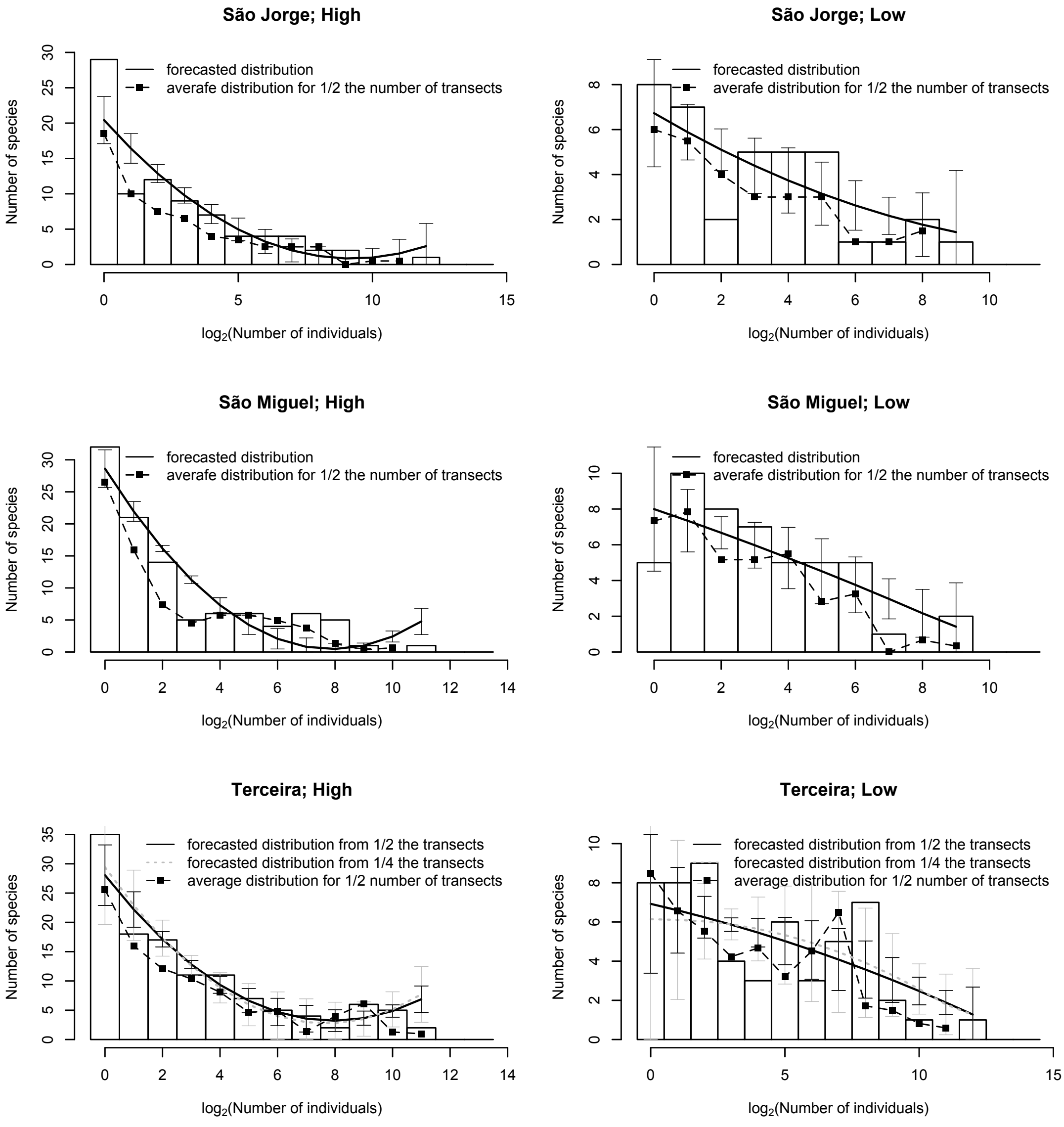

Figure S3. The histograms correspond to the species abundance distribution of the full dataset of each island, for high dispersal ability species (left column) and low dispersal ability species (right column). The black squares and the dashed lines correspond to the average species abundance distribution obtained from all the concentric combinations (see main text) of half the number of transects; because Terceira island has 39 transects we used 20 transects for “half” the data, all the other islands have an even number of transects. The full lines show the forecasted distributions and the arrows correspond to two standard deviations. For Pico and

Terceira islands the dotted (gray) line is the forecasted species relative abundance obtained from one quarter of the total number of transects (4 and 10 transects for Pico and Terceira islands, respectively).

Figures with results for all sampled islands

Here we complement Figs 3 (Fig. S4), 4 (Fig. S5), 5 (Fig. S6 and S7) and 6 (Fig. S8) of the main text by showing the results to all islands where data were collected.

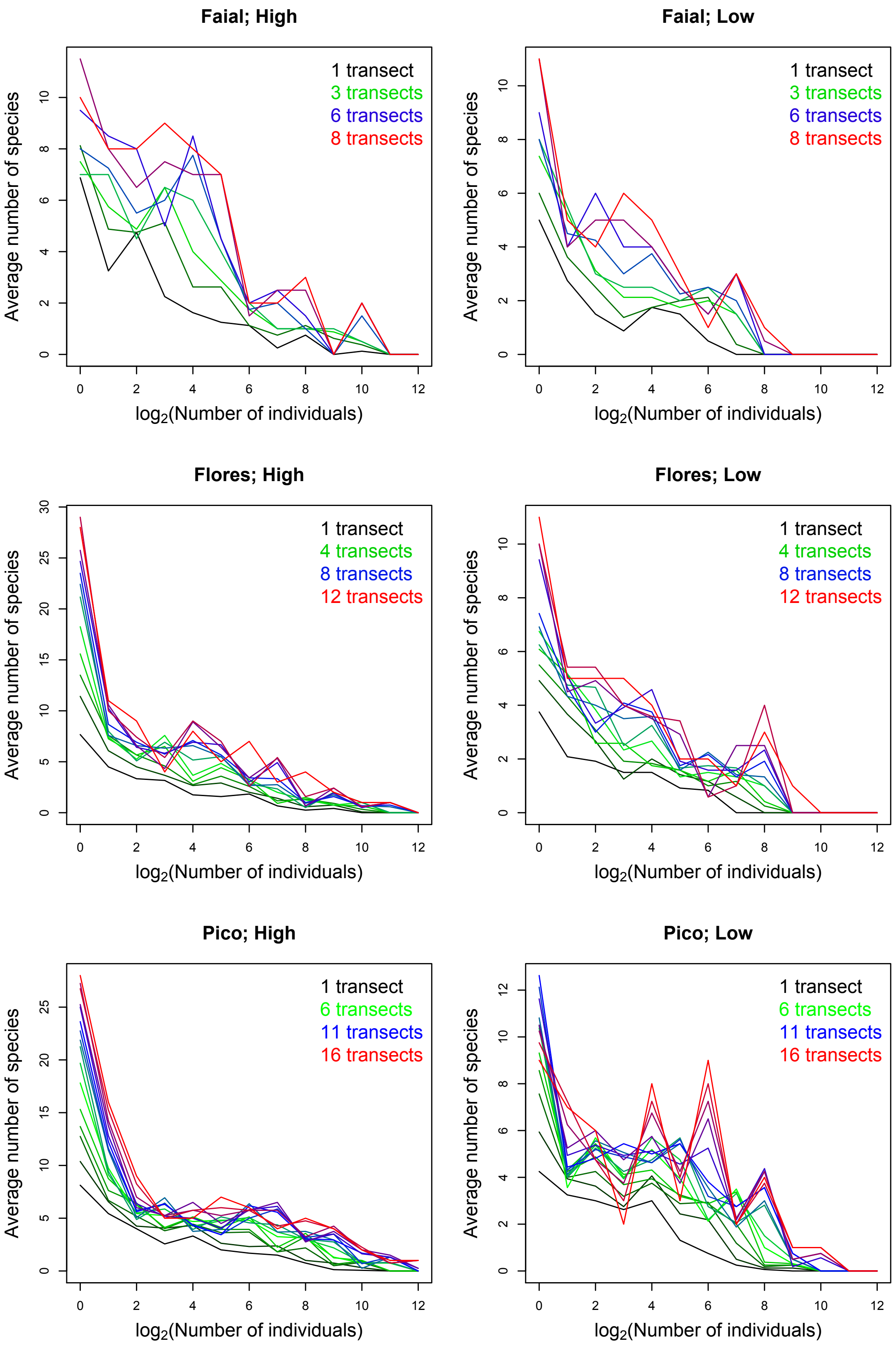
Faial; High
1 transect
3 transects
6 transects
8 transects
Average number of species
log2(Number of individuals)
Faial; Low
1 transect
3 transects
6 transects
8 transects
Average number of species
log2(Number of individuals)
Flores; High
1 transect
4 transects
8 transects
12 transects
Average number of species
log2(Number of individuals)
Flores; Low
1 transect
4 transects
8 transects
12 transects
Average number of species
log2(Number of individuals)
Pico; High
1 transect
6 transects
11 transects
16 transects
Average number of species
log2(Number of individuals)
Pico; Low
1 transect
6 transects
11 transects
16 transects
Average number of species
log2(Number of individuals)

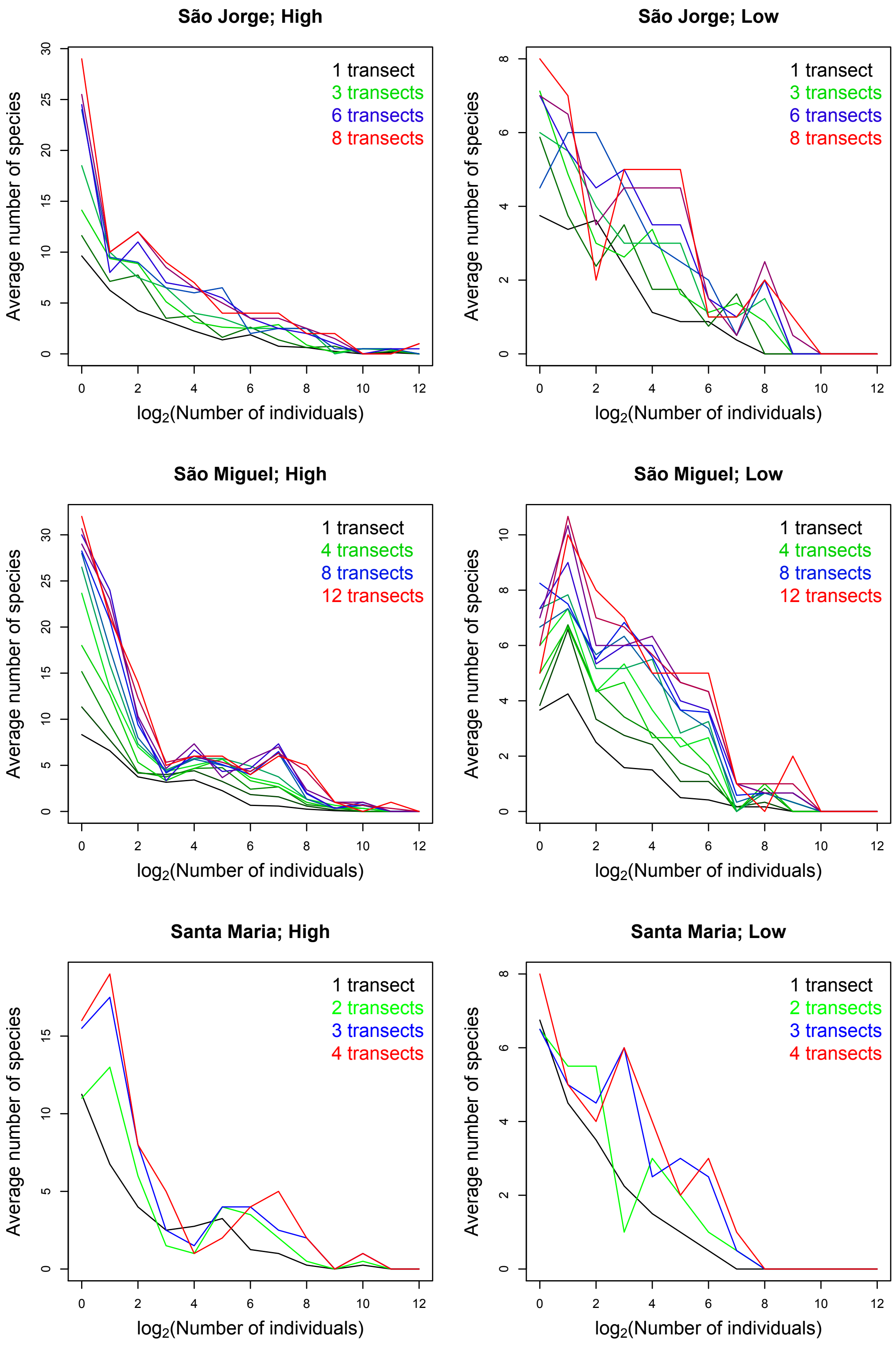
São Jorge; High
Average number of species
log2(Number of individuals)
1 transect
3 transects
6 transects
8 transects
São Jorge; Low
1 transect
3 transects
6 transects
8 transects
São Miguel; High
1 transect
4 transects
8 transects
12 transects
São Miguel; Low
1 transect
4 transects
8 transects
12 transects
Santa Maria; High
1 transect
2 transects
3 transects
4 transects
Santa Maria; Low
1 transect
2 transects
3 transects
4 transects

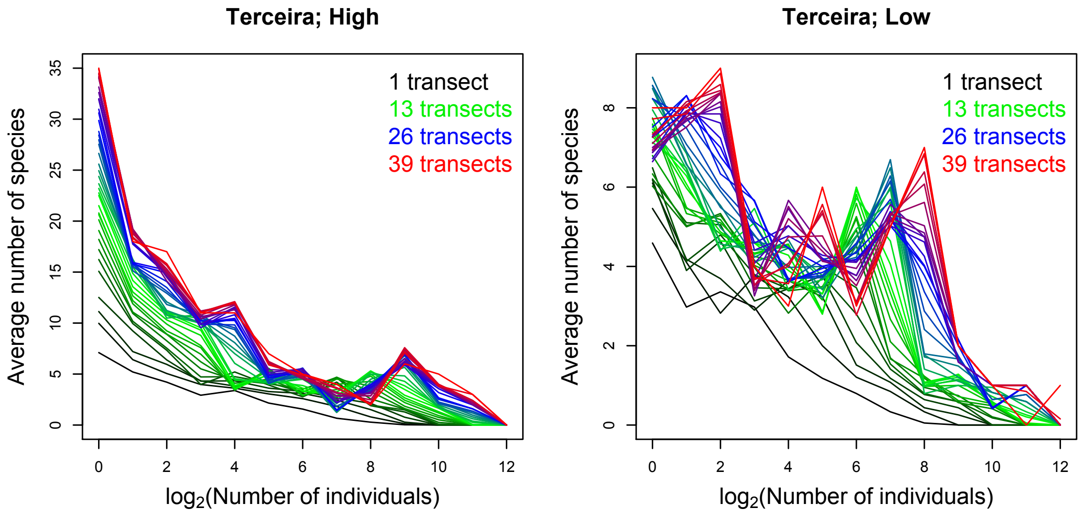

Figure S4. Species abundance distributions for arthropods species of all sampled Azorean islands. Each curve corresponds to the average of all possible SAD curves

obtained by using the concentric procedure as explained in Methods. The *x*-axis corresponds to classes of the logarithm of base 2 of the number of individuals as follows: 1 individual, 2 to 3 individuals, 4 to 7 individuals, *et seq*. In order to better illustrate the evolution of the shapes of the distributions, the curves have a gradient of colours going from black (the smallest number of transects), through green and blue, to red (the largest number of transects).

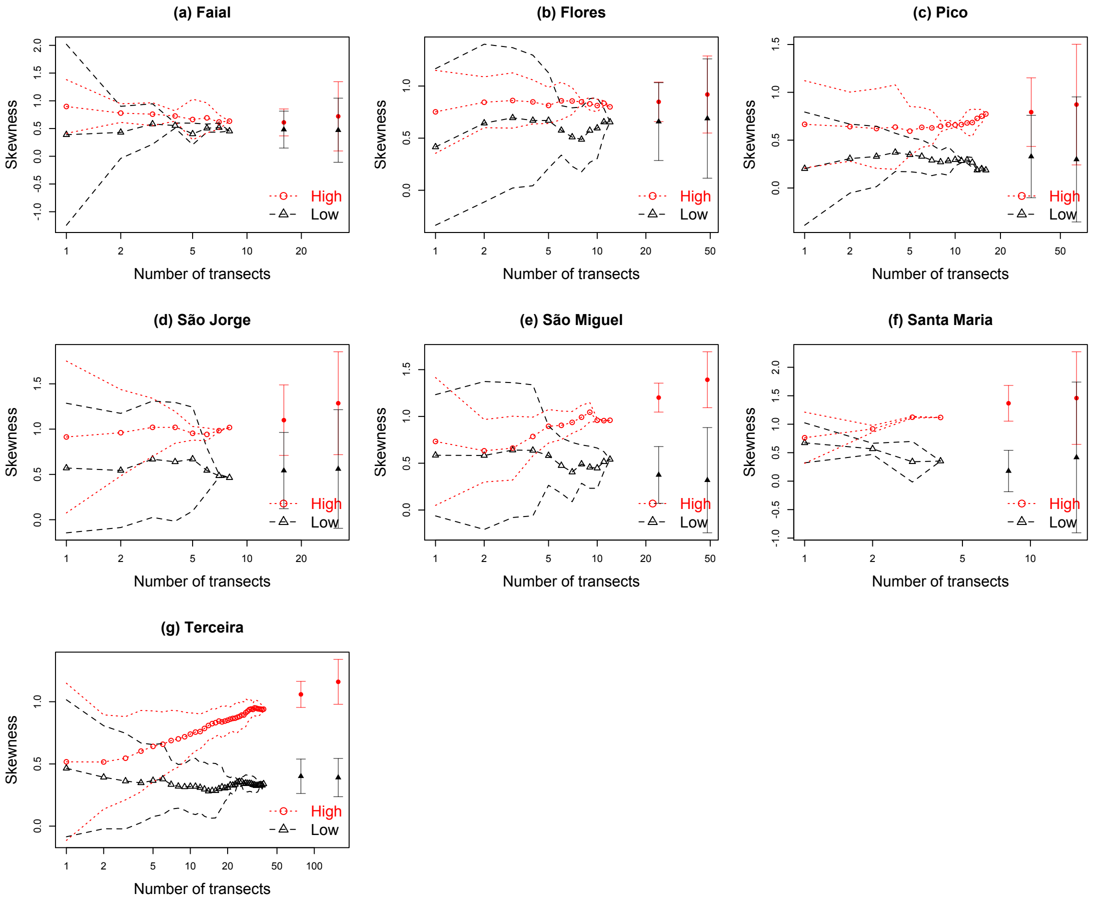

Figure S5. Evolution of the skewness (third standardized moment) of the arthropods SAD of all sampled Azorean islands as a function of the number of transects, in red for high dispersal and in black for low dispersal ability arthropod species. The circles and triangles correspond to the average values of the skewness calculated from the

SAD obtained from all possible addition of transects using the concentric procedure as explained in the main text. The dot and dash lines correspond to 2 standard deviation confidence intervals. The two rightmost bold dots are the skewness estimated from extrapolated SADs and the arrow bars correspond to two standard deviations.

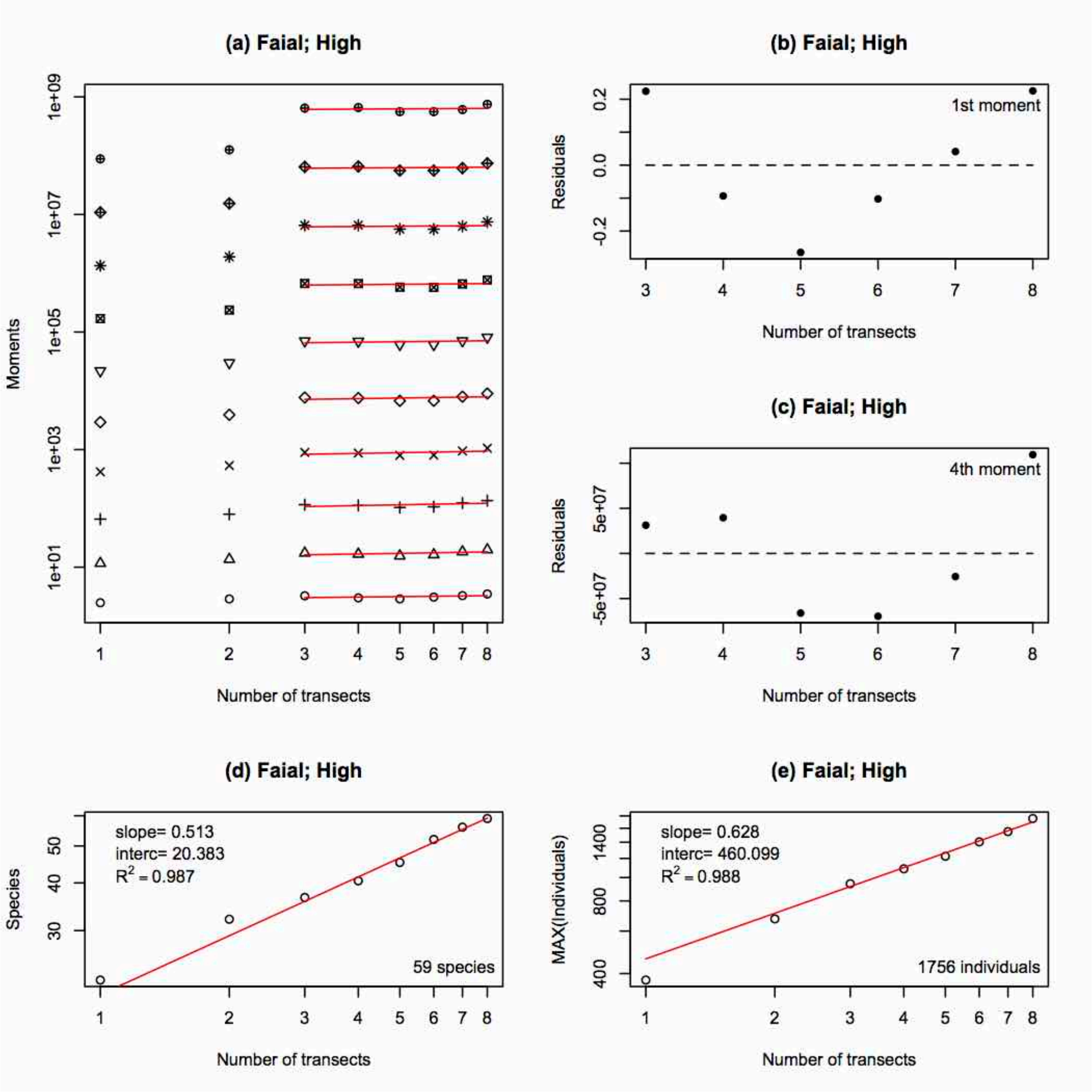

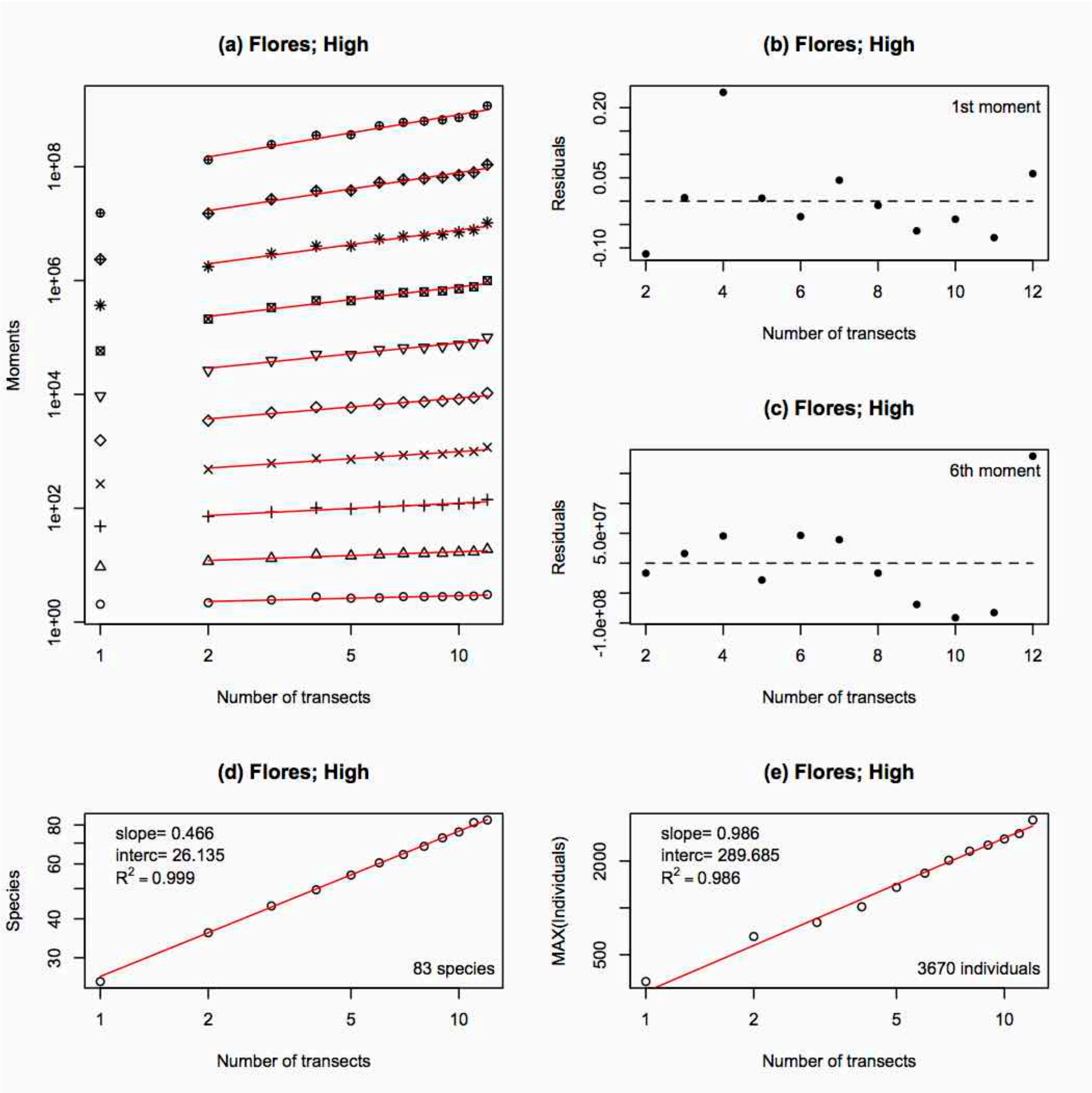
(a) Flores; High
Moments
Number of transects
(b) Flores; High
1st moment
Residuals
Number of transects
(c) Flores; High
6th moment
Residuals
Number of transects
(d) Flores; High
slope= 0.466
interc= 26.135
R2 = 0.999
83 species
Species
Number of transects
(e) Flores; High
slope= 0.986
interc= 289.685
R2 = 0.986
3670 individuals
MAX(Individuals)
Number of transects

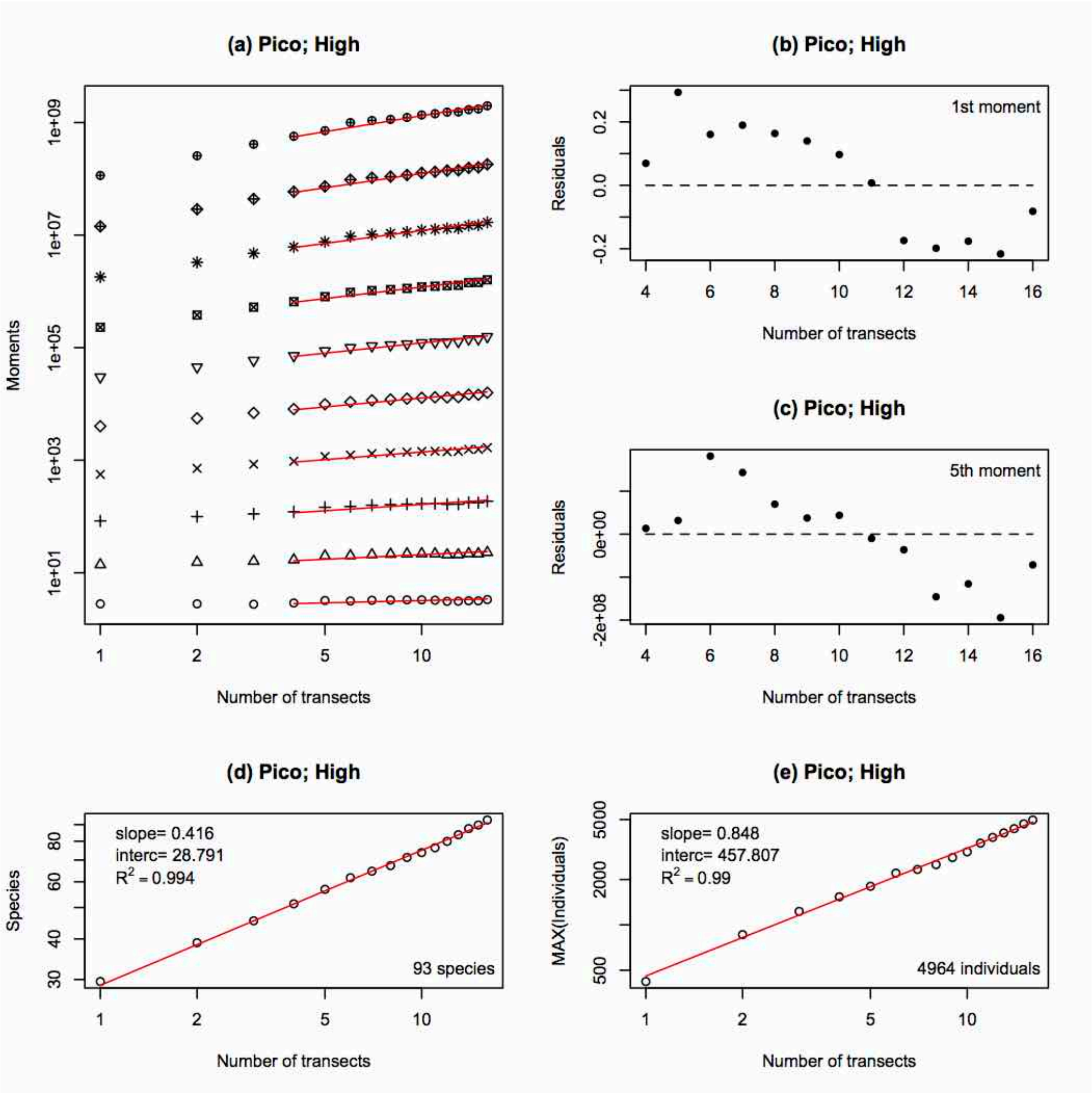
(a) Pico; High
Moments
1e+01
1e+03
1e+05
1e+07
1e+09
1
2
5
10
Number of transects
(b) Pico; High
1st moment
Residuals
-0.2
0.0
0.2
4
6
8
10
12
14
16
Number of transects
(c) Pico; High
5th moment
Residuals
-2e+08
0e+00
4
6
8
10
12
14
16
Number of transects
(d) Pico; High
slope= 0.416
interc= 28.791
R^2 = 0.994
93 species
Species
30
40
60
80
1
2
5
10
Number of transects
(e) Pico; High
slope= 0.848
interc= 457.807
R^2 = 0.99
4964 individuals
MAX(Individuals)
500
2000
5000
1
2
5
10
Number of transects

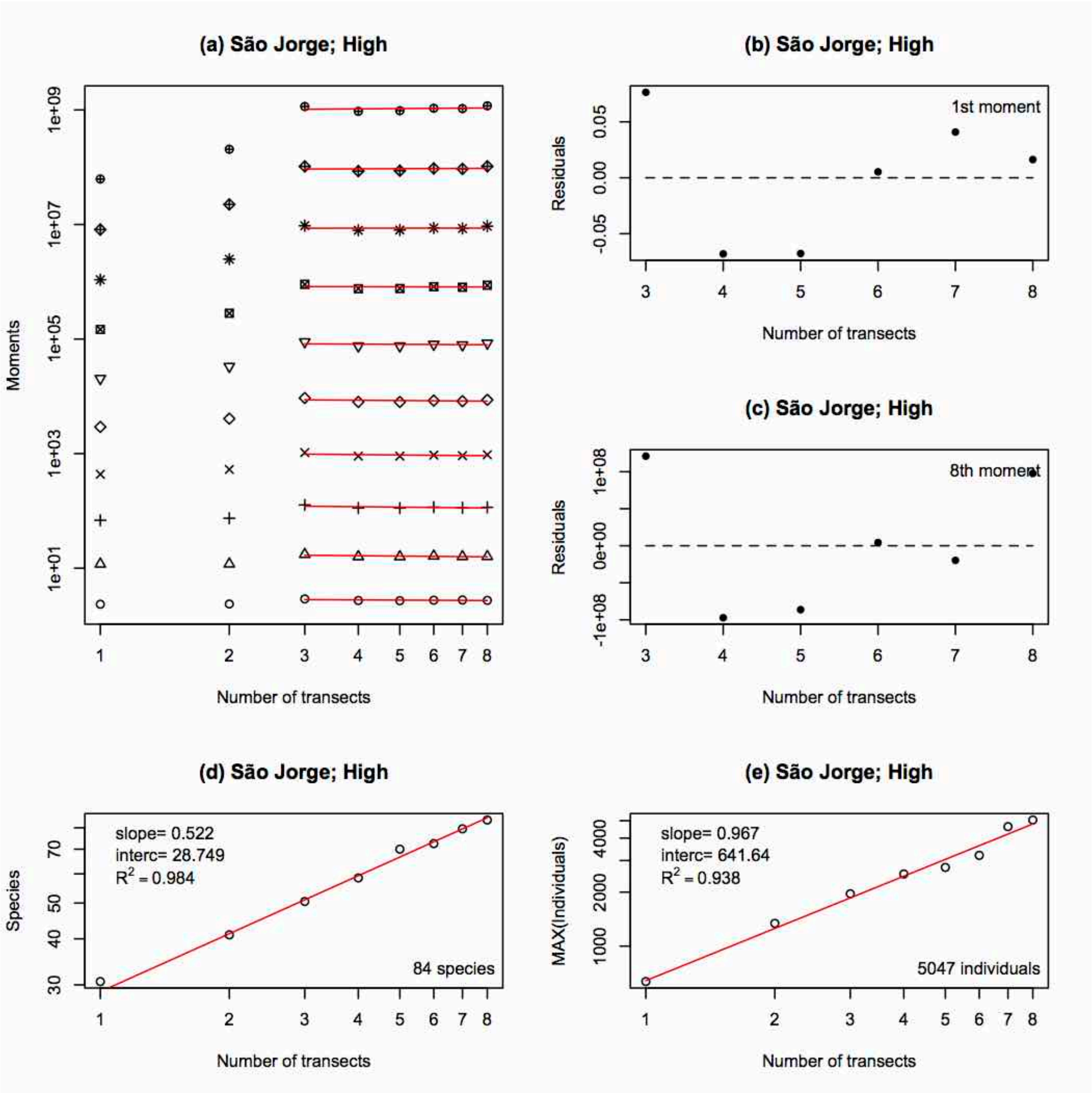
(a) São Jorge; High
Moments
Number of transects
(b) São Jorge; High
Residuals
1st moment
Number of transects
(c) São Jorge; High
Residuals
8th moment
Number of transects
(d) São Jorge; High
slope= 0.522
interc= 28.749
R² = 0.984
84 species
Species
Number of transects
(e) São Jorge; High
slope= 0.967
interc= 641.64
R² = 0.938
5047 individuals
MAX(Individuals)
Number of transects

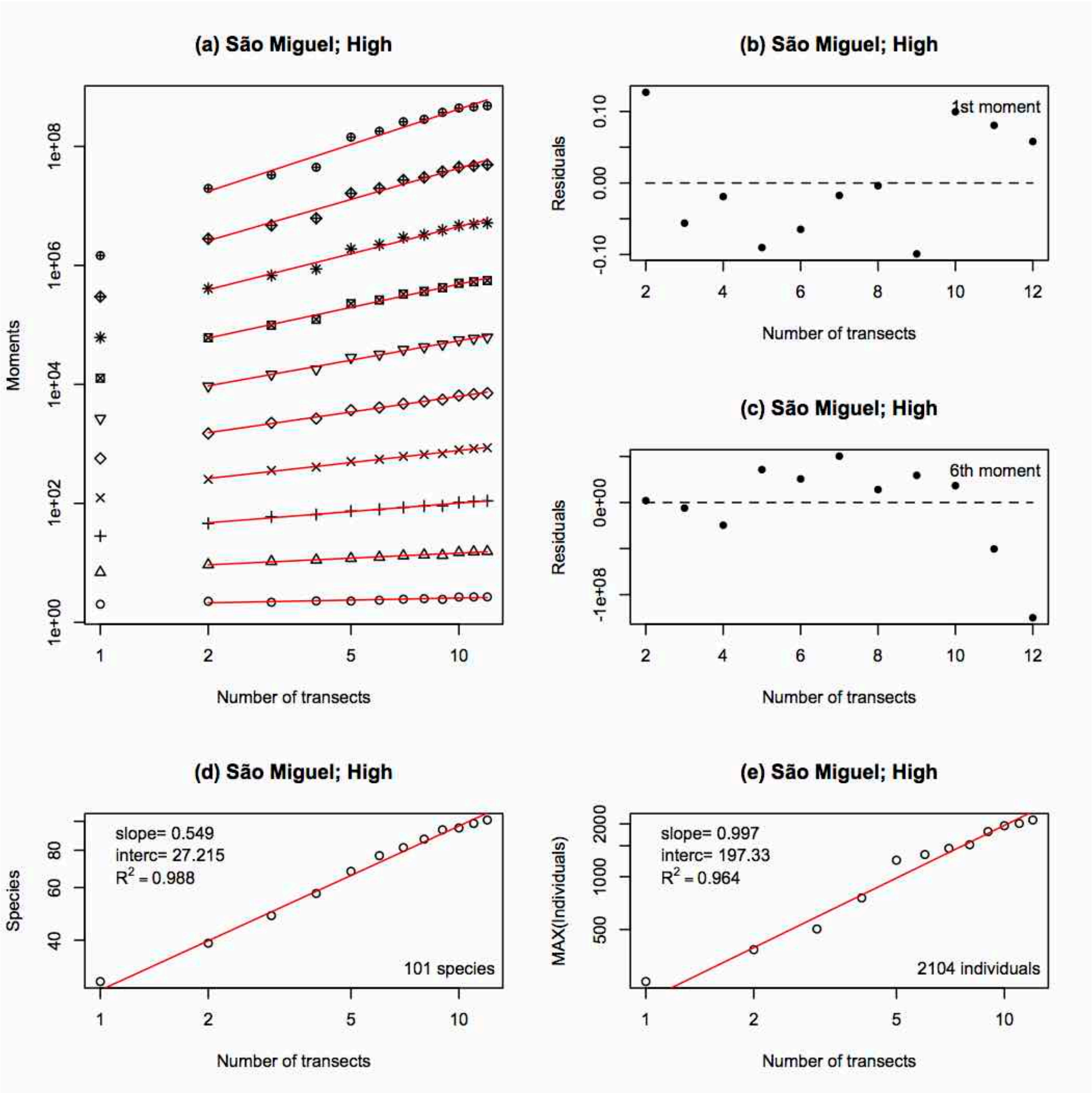
(a) São Miguel; High
Moments
1e+00
1e+02
1e+04
1e+06
1e+08
1
2
5
10
Number of transects
(b) São Miguel; High
Residuals
-0.10
0.00
0.10
1st moment
2
4
6
8
10
12
Number of transects
(c) São Miguel; High
Residuals
-1e+08
0e+00
6th moment
2
4
6
8
10
12
Number of transects
(d) São Miguel; High
Species
40
60
80
slope= 0.549
interc= 27.215
R2 = 0.988
101 species
1
2
5
10
Number of transects
(e) São Miguel; High
MAX(Individuals)
500
1000
2000
slope= 0.997
interc= 197.33
R2 = 0.964
2104 individuals
1
2
5
10
Number of transects

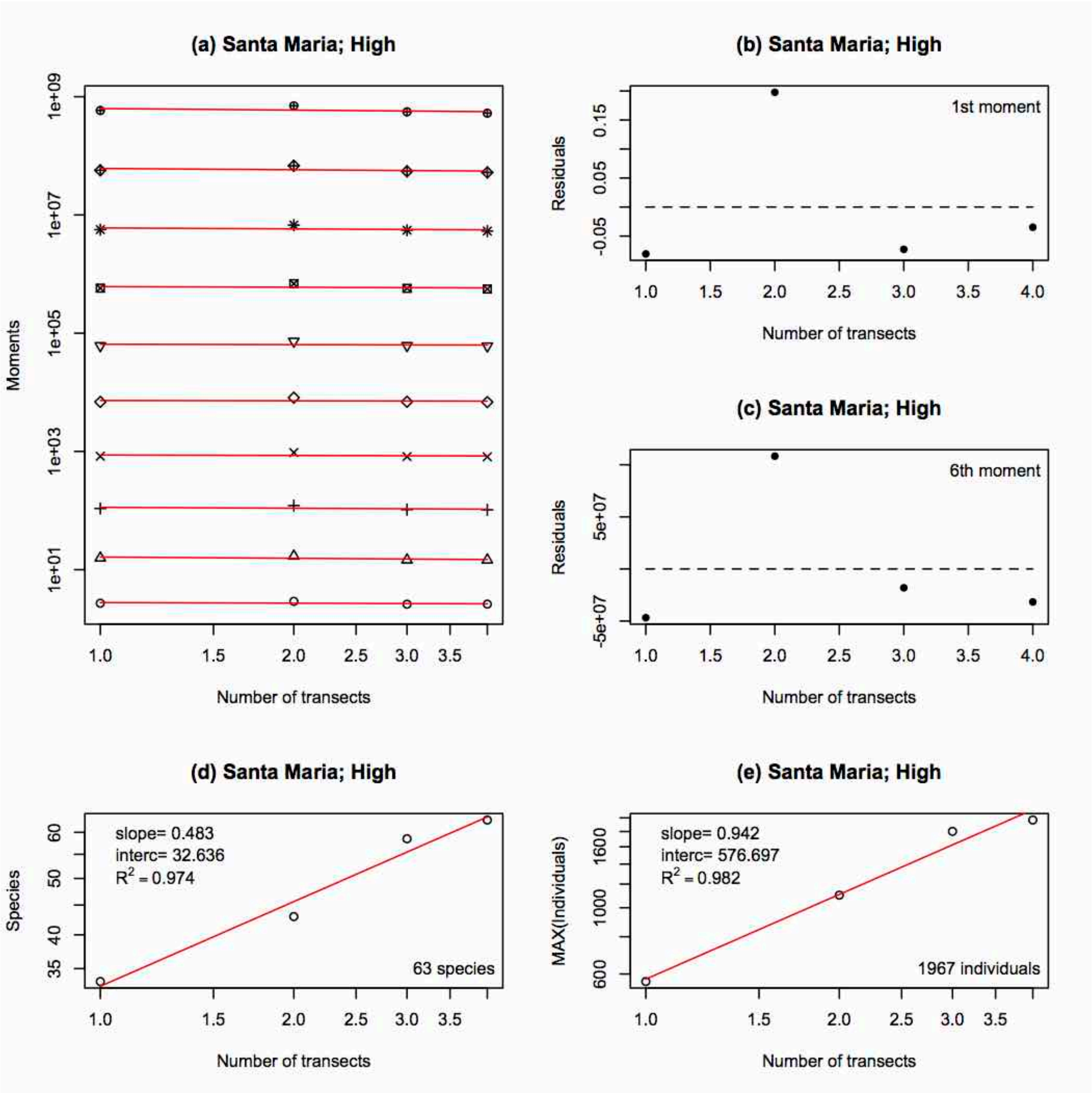

(a) Santa Maria; High
Moments
1e+09
1e+07
1e+05
1e+03
1e+01
1.0
1.5
2.0
2.5
3.0
3.5
Number of transects
(b) Santa Maria; High
Residuals
0.15
0.05
-0.05
1st moment
1.0
1.5
2.0
2.5
3.0
3.5
4.0
Number of transects
(c) Santa Maria; High
Residuals
5e+07
-5e+07
6th moment
1.0
1.5
2.0
2.5
3.0
3.5
4.0
Number of transects
(d) Santa Maria; High
Species
60
50
40
35
slope= 0.483
interc= 32.636
R2 = 0.974
63 species
1.0
1.5
2.0
2.5
3.0
3.5
Number of transects
(e) Santa Maria; High
MAX(Individuals)
1600
1000
600
slope= 0.942
interc= 576.697
R2 = 0.982
1967 individuals
1.0
1.5
2.0
2.5
3.0
3.5
Number of transects

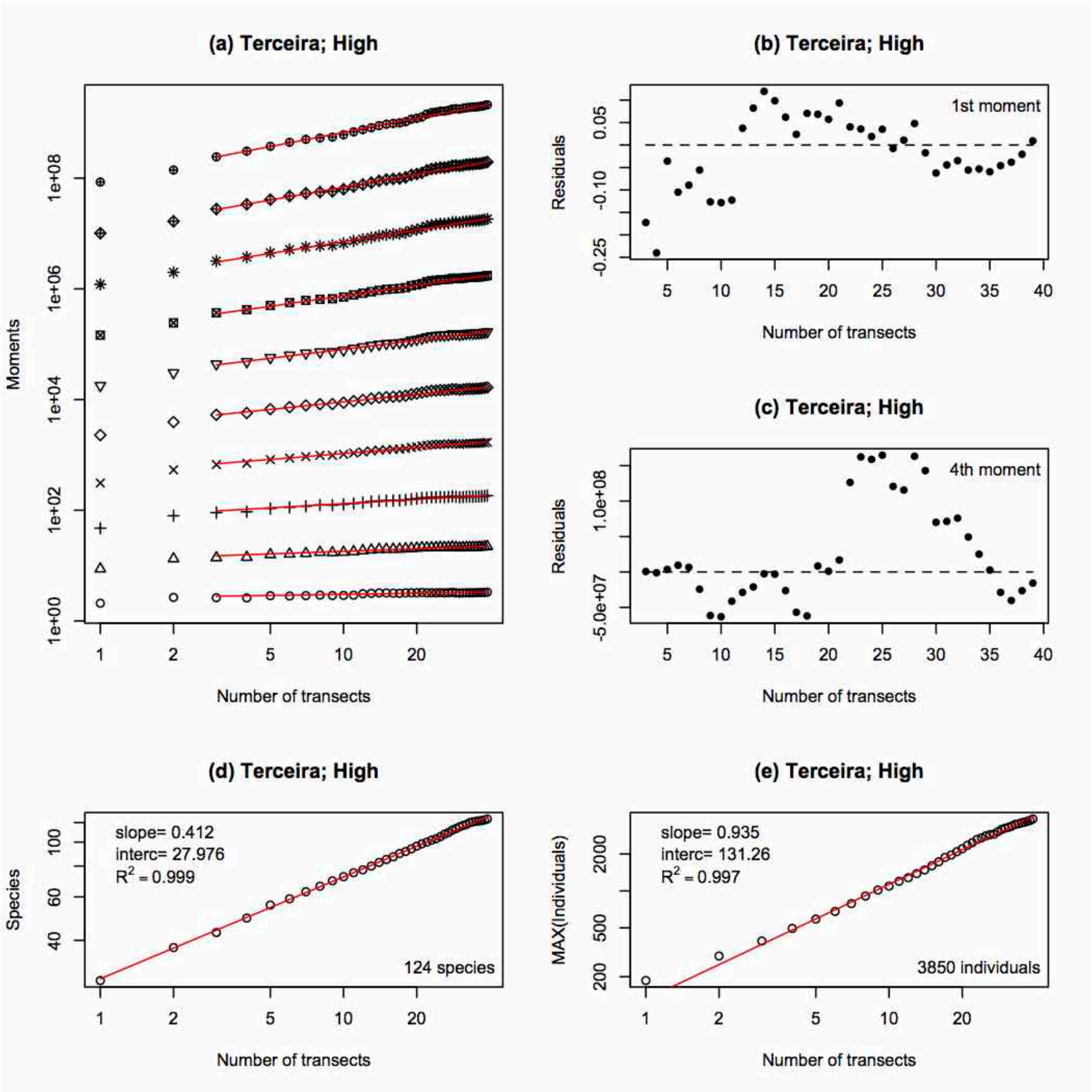

Figure S6. Plots (a) show in double logarithmic scales the first 10 integer moments for high dispersal ability arthropod species of all sampled Azorean islands as a function of a sequence of added transects using the concentric procedure, as explained in the main text. The order of the moments increases when we go from the bottom to the top lines and the moments shown here correspond to a sequence of added transects. Plots (b) and (c) show the residuals from the linear regression of the logarithm of the moments of order 1 and of the highest moment used in the extrapolations (see Figure S7), respectively. Plot (d) is the species area relationship and the red line corresponds to fit obtained with least squares assuming a power law relationship, $S = cA^z$. Plot (e) shows the logarithm of the number of individuals of the most abundant species as a

function of the logarithm of the area, and the red line was obtained by least squares assuming a power law relationship.

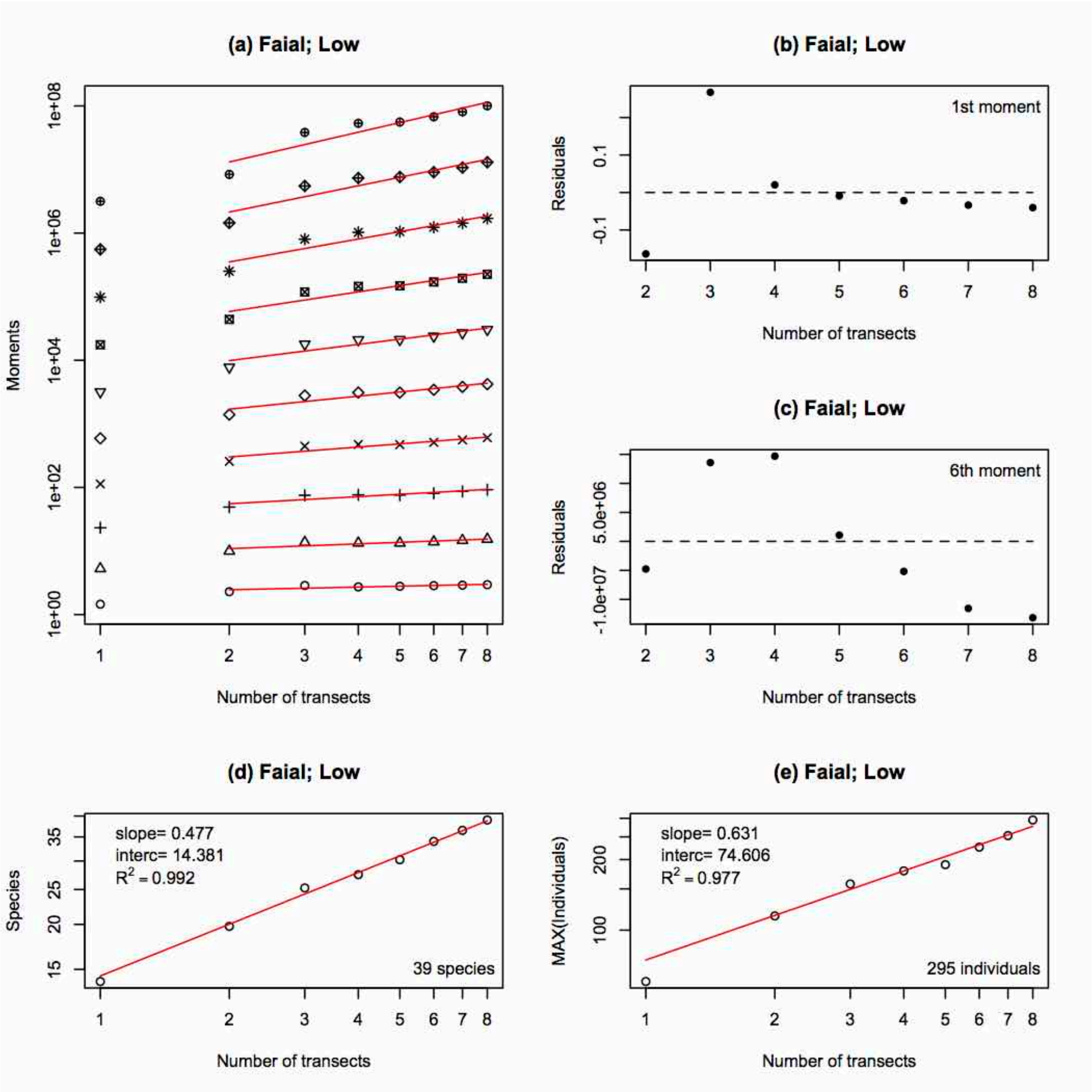

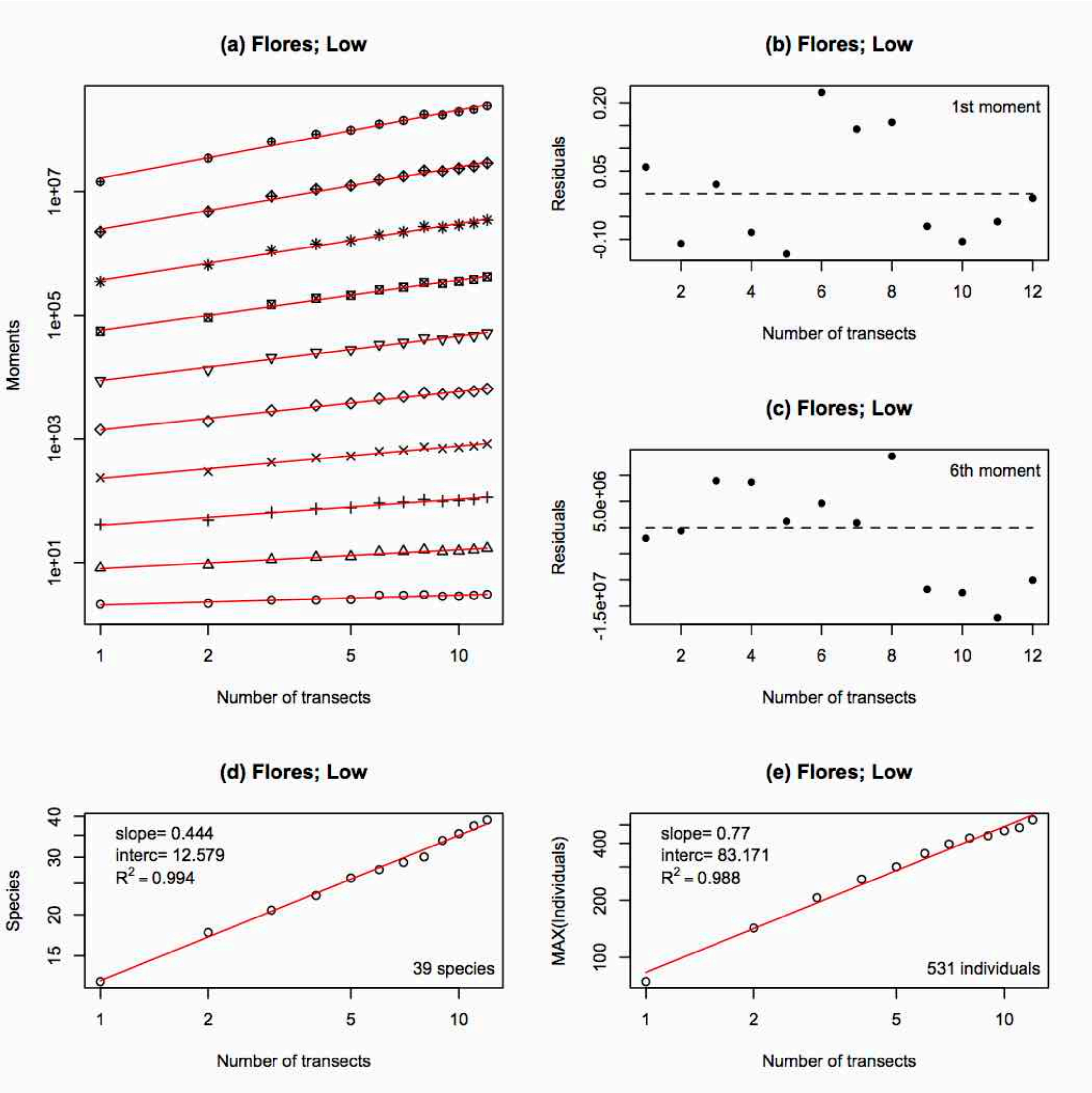
(a) Flores; Low
Moments
1e+01
1e+03
1e+05
1e+07
1
2
5
10
Number of transects
(b) Flores; Low
Residuals
-0.10
0.05
0.20
1st moment
2
4
6
8
10
12
Number of transects
(c) Flores; Low
Residuals
-1.5e+07
5.0e+06
6th moment
2
4
6
8
10
12
Number of transects
(d) Flores; Low
Species
15
20
30
40
slope= 0.444
interc= 12.579
R2 = 0.994
39 species
1
2
5
10
Number of transects
(e) Flores; Low
MAX(Individuals)
100
200
400
slope= 0.77
interc= 83.171
R2 = 0.988
531 individuals
1
2
5
10
Number of transects

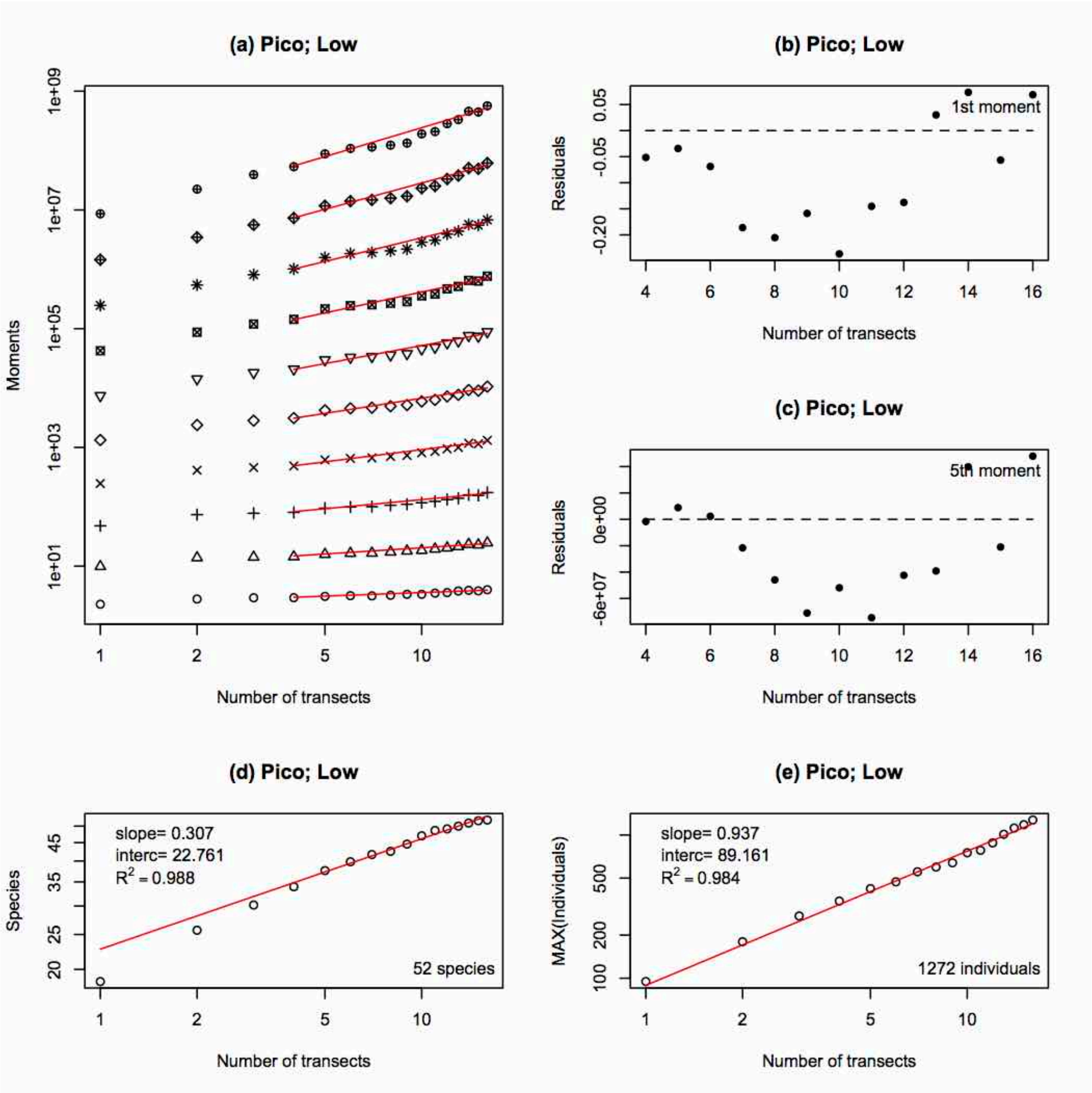
(a) Pico; Low
Moments
Number of transects
(b) Pico; Low
Residuals
1st moment
Number of transects
(c) Pico; Low
Residuals
5th moment
Number of transects
(d) Pico; Low
Species
slope= 0.307
interc= 22.761
$R^2 = 0.988$
52 species
Number of transects
(e) Pico; Low
MAX(Individuals)
slope= 0.937
interc= 89.161
$R^2 = 0.984$
1272 individuals
Number of transects

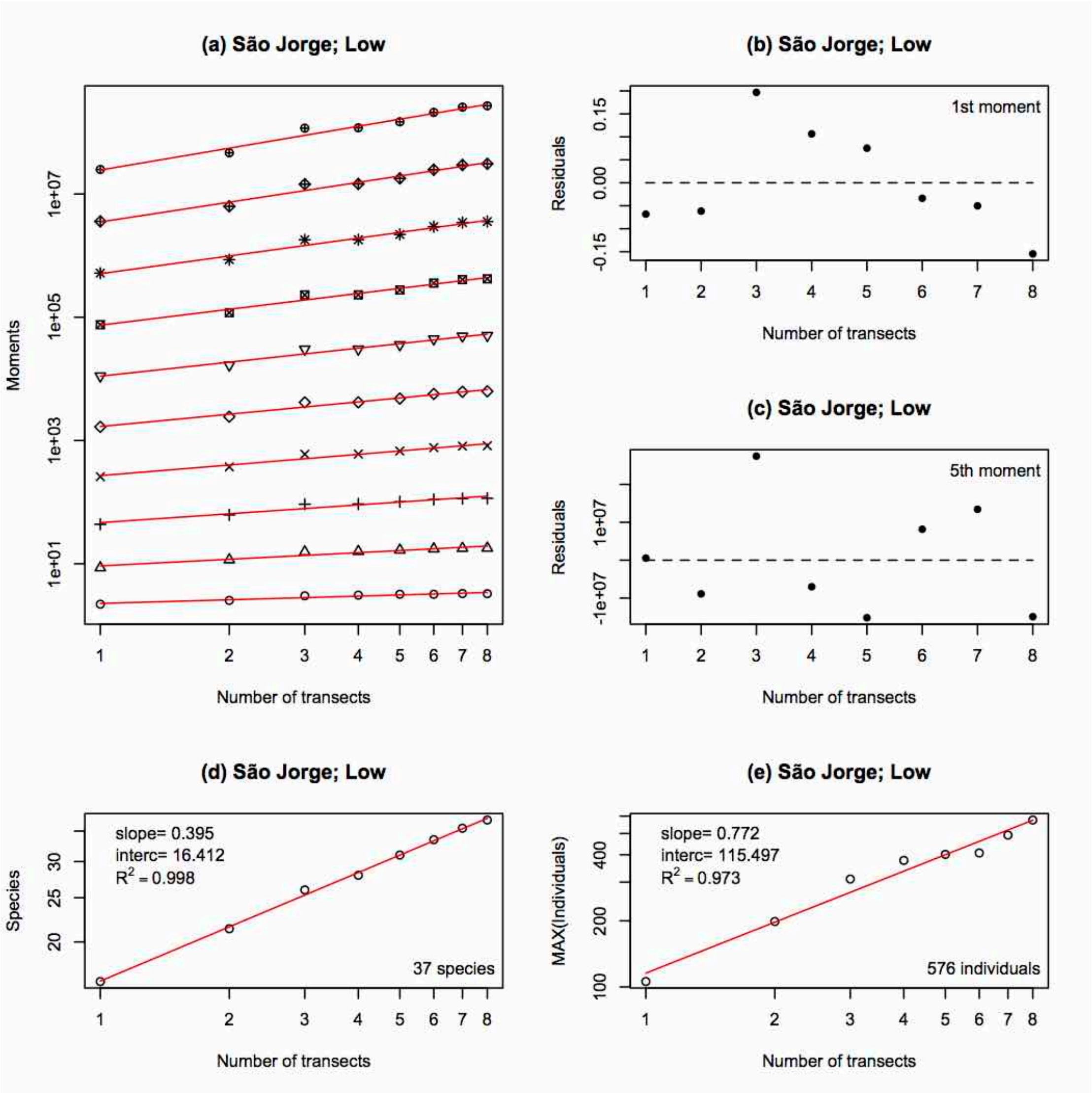
(a) São Jorge; Low
Moments
1e+01
1e+03
1e+05
1e+07
Number of transects
(b) São Jorge; Low
Residuals
1st moment
-0.15
0.00
0.15
Number of transects
(c) São Jorge; Low
Residuals
5th moment
-1e+07
1e+07
Number of transects
(d) São Jorge; Low
Species
slope= 0.395
interc= 16.412
R2 = 0.998
37 species
Number of transects
(e) São Jorge; Low
MAX(Individuals)
slope= 0.772
interc= 115.497
R2 = 0.973
576 individuals
Number of transects

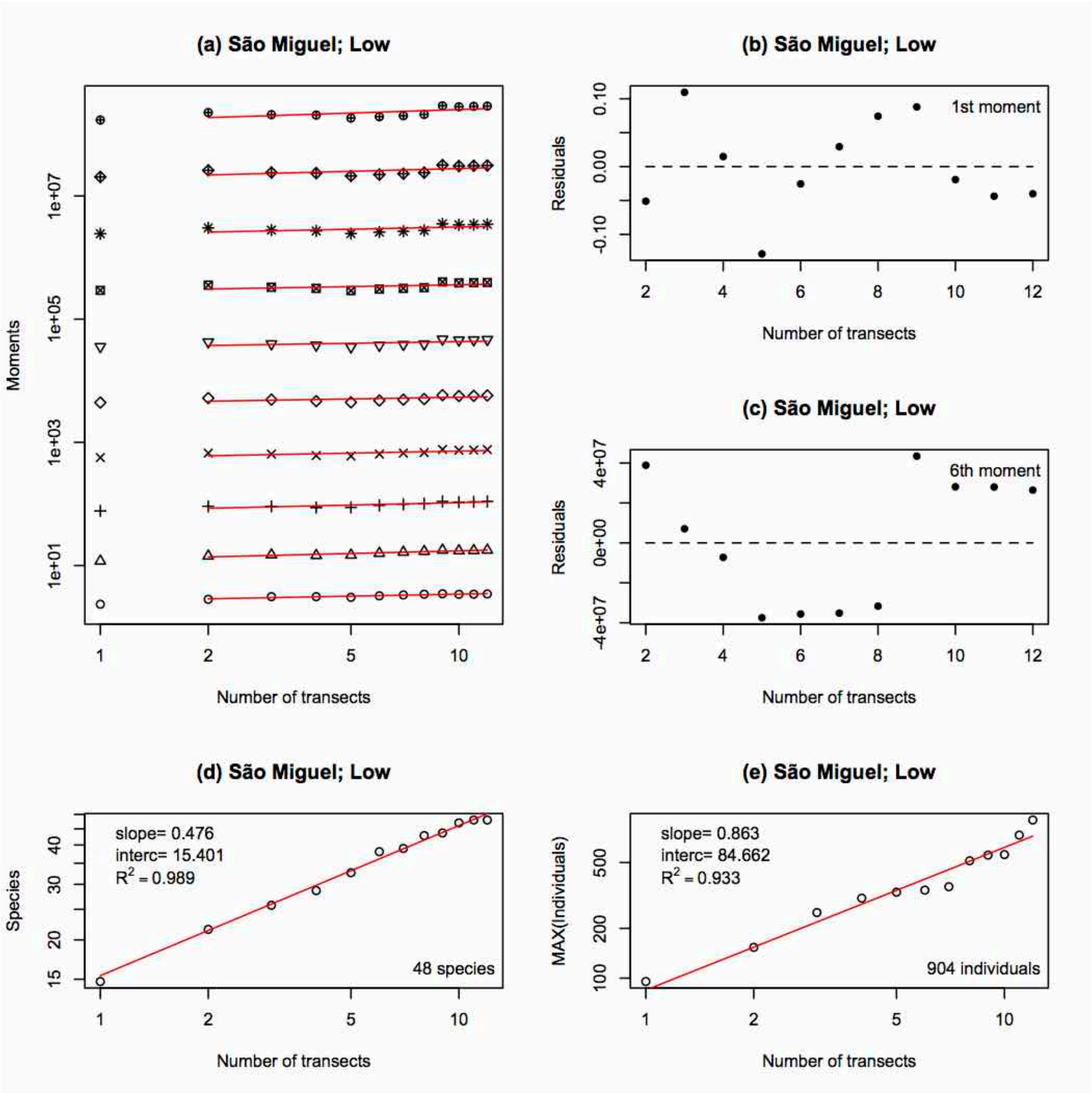

(a) São Miguel; Low
Moments
1e+01
1e+03
1e+05
1e+07
Number of transects
(b) São Miguel; Low
Residuals
1st moment
Number of transects
(c) São Miguel; Low
Residuals
6th moment
Number of transects
(d) São Miguel; Low
Species
slope= 0.476
interc= 15.401
$R^2 = 0.989$
48 species
Number of transects
(e) São Miguel; Low
MAX(Individuals)
slope= 0.863
interc= 84.662
$R^2 = 0.933$
904 individuals
Number of transects

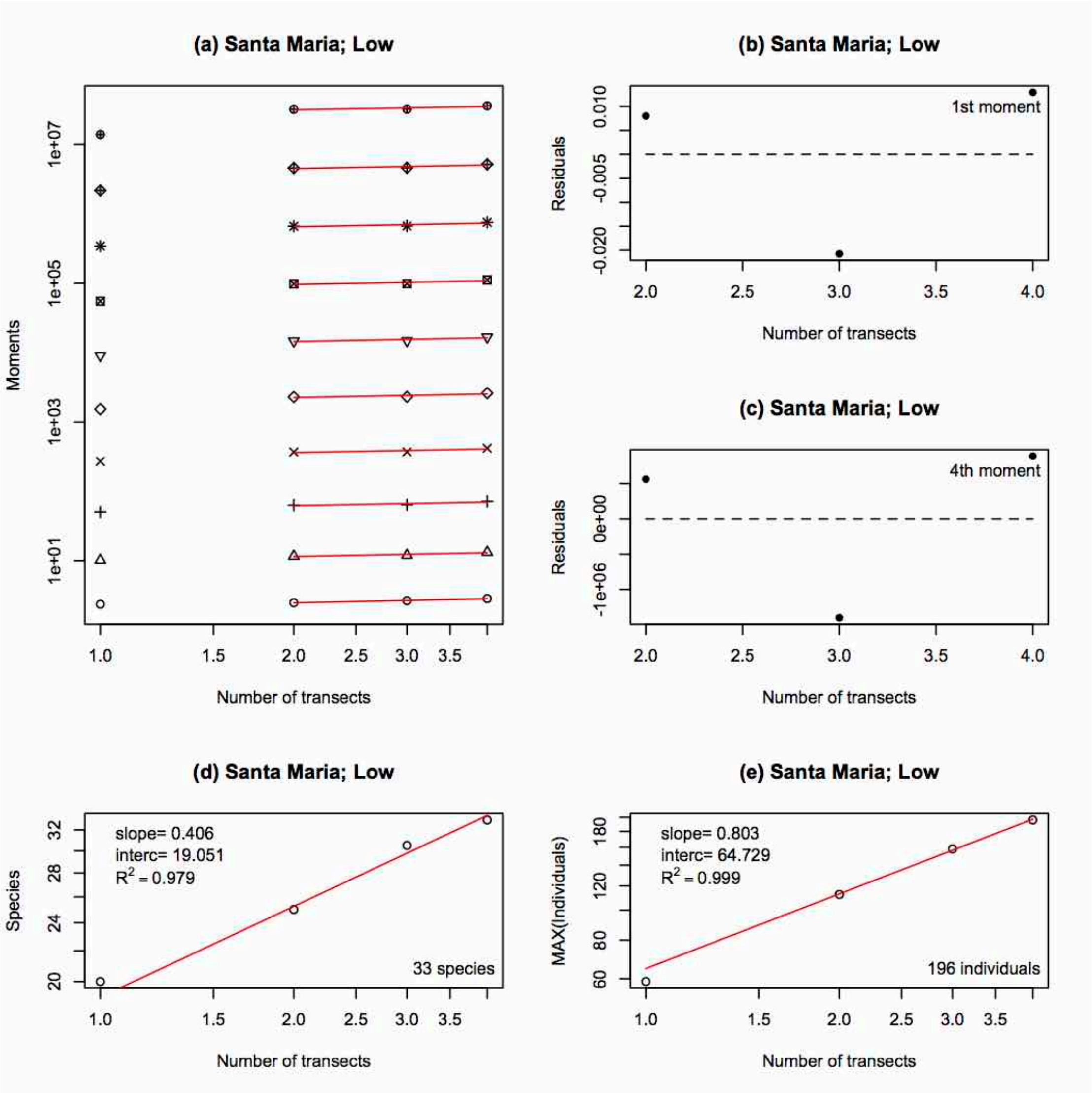
(a) Santa Maria; Low
Moments
1e+01
1e+03
1e+05
1e+07
1.0
1.5
2.0
2.5
3.0
3.5
Number of transects
(b) Santa Maria; Low
Residuals
-0.020
-0.005
0.010
1st moment
2.0
2.5
3.0
3.5
4.0
Number of transects
(c) Santa Maria; Low
Residuals
-1e+06
0e+00
4th moment
2.0
2.5
3.0
3.5
4.0
Number of transects
(d) Santa Maria; Low
Species
20
24
28
32
slope= 0.406
interc= 19.051
R2 = 0.979
33 species
1.0
1.5
2.0
2.5
3.0
3.5
Number of transects
(e) Santa Maria; Low
MAX(Individuals)
60
80
120
180
slope= 0.803
interc= 64.729
R2 = 0.999
196 individuals
1.0
1.5
2.0
2.5
3.0
3.5
Number of transects

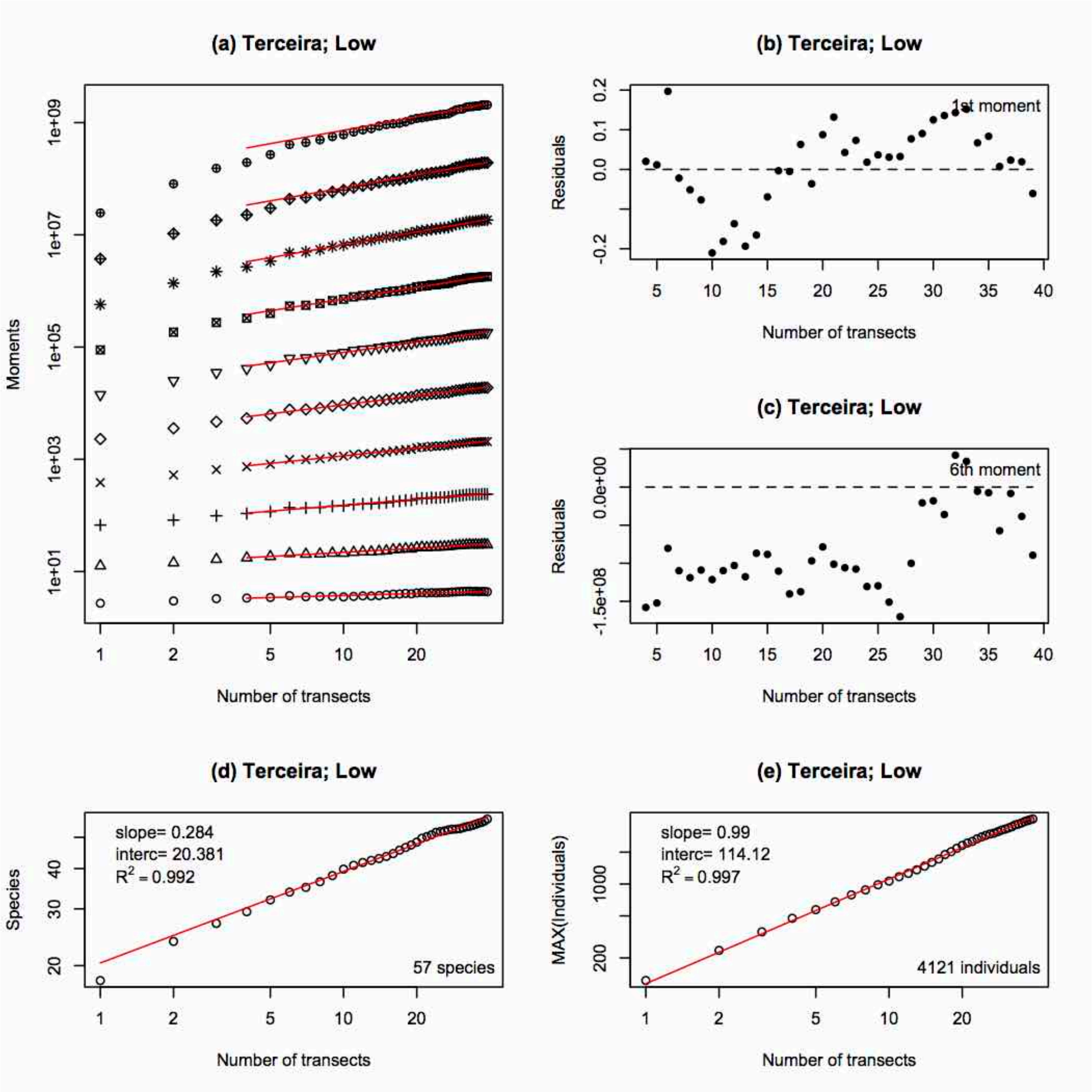

Figure S7. The same as Figure S6 but for low dispersal ability species.

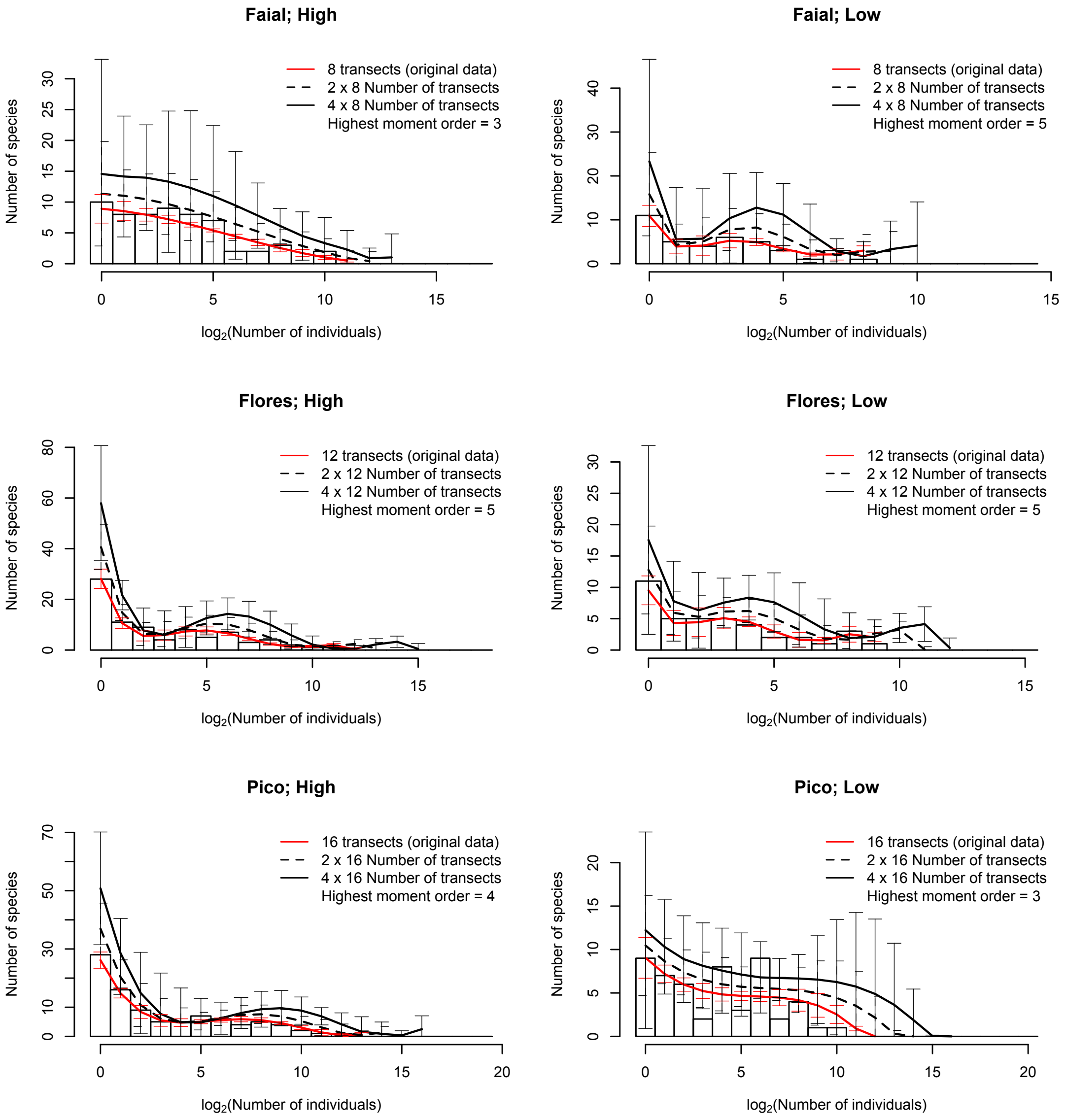
Faial; High
8 transects (original data)
2 x 8 Number of transects
4 x 8 Number of transects
Highest moment order = 3
Number of species
log2(Number of individuals)
Faial; Low
8 transects (original data)
2 x 8 Number of transects
4 x 8 Number of transects
Highest moment order = 5
Number of species
log2(Number of individuals)
Flores; High
12 transects (original data)
2 x 12 Number of transects
4 x 12 Number of transects
Highest moment order = 5
Number of species
log2(Number of individuals)
Flores; Low
12 transects (original data)
2 x 12 Number of transects
4 x 12 Number of transects
Highest moment order = 5
Number of species
log2(Number of individuals)
Pico; High
16 transects (original data)
2 x 16 Number of transects
4 x 16 Number of transects
Highest moment order = 4
Number of species
log2(Number of individuals)
Pico; Low
16 transects (original data)
2 x 16 Number of transects
4 x 16 Number of transects
Highest moment order = 3
Number of species
log2(Number of individuals)

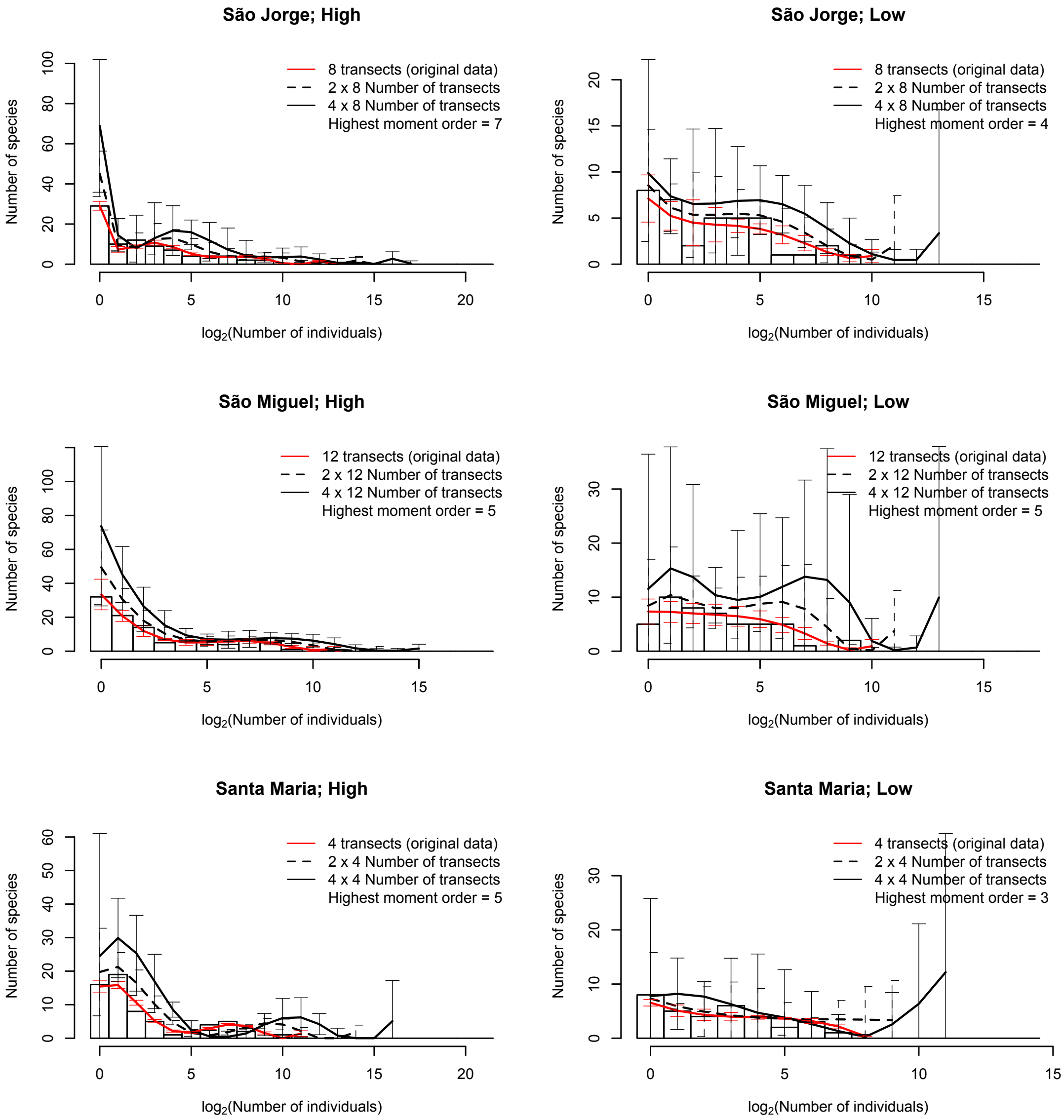
São Jorge; High
8 transects (original data)
2 x 8 Number of transects
4 x 8 Number of transects
Highest moment order = 7
Number of species
log2(Number of individuals)
São Jorge; Low
8 transects (original data)
2 x 8 Number of transects
4 x 8 Number of transects
Highest moment order = 4
Number of species
log2(Number of individuals)
São Miguel; High
12 transects (original data)
2 x 12 Number of transects
4 x 12 Number of transects
Highest moment order = 5
Number of species
log2(Number of individuals)
São Miguel; Low
12 transects (original data)
2 x 12 Number of transects
4 x 12 Number of transects
Highest moment order = 5
Number of species
log2(Number of individuals)
Santa Maria; High
4 transects (original data)
2 x 4 Number of transects
4 x 4 Number of transects
Highest moment order = 5
Number of species
log2(Number of individuals)
Santa Maria; Low
4 transects (original data)
2 x 4 Number of transects
4 x 4 Number of transects
Highest moment order = 3
Number of species
log2(Number of individuals)

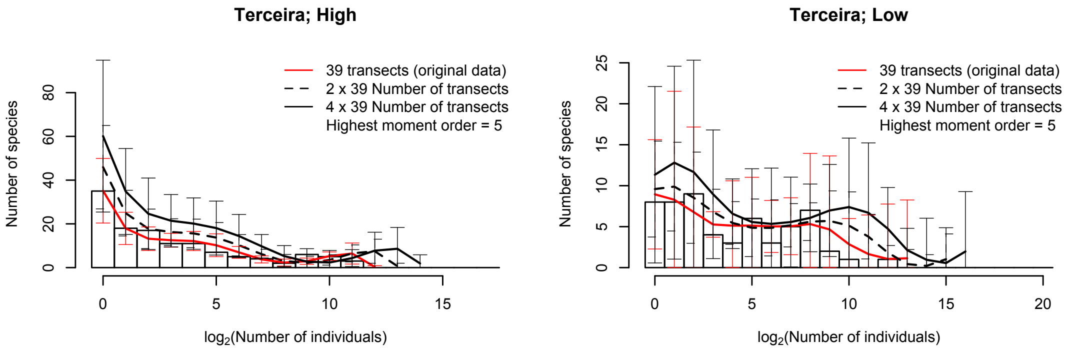

Figure S8. Each of the plots corresponds to the histograms of the species abundance distribution of all transects combined of arthropod species of the samples Azorean islands. The red curve is the distribution estimated from all transects using the scaled Tchebichef moments and polynomials. The black and full dashed lines are the forecasted distributions for two and four times the original number of transects and they correspond to the mean value of the distributions obtained with all possible additions of transect sequences; these distributions were used to obtain 2 standard deviations shown by the error bars. The x-axis corresponds to classes of the logarithm

of the number of individuals as follows: 1 individual, 2 to 3 individuals, 4 to 7 individuals, *et seq*.

Sensitivity analysis of the impact of the choice of dispersal ability on the evolution of the skewness of the SADs

Dispersal ability forms a continuous. However, it is at the moment impossible from a practical point of view to quantify the dispersal ability for the 337 species of arthropods used in this work. Therefore, we described dispersal ability in a binary way: high and low dispersal ability. If we had access to more precise information on the dispersal ability for each species we could repeat the analysis by using only those sets of species at the extremes of the dispersal continuum (after choosing some thresholds), but we believe that in such a case we would have observed very similar trends, just more pronounced. We hope that one day such information will become available, so that these analyses become possible to perform. Here we assess possible sources of biases due to the miss-identification of dispersal ability.

Although we dealt with a large number of species, only 19 (approximately 6% of all species, with 3 identified as "High" and 16 as "Low" dispersal) raised doubts about their dispersal category; these are listed in Table S2. Therefore, we first assessed what would have been the impact of changing the dispersal "type" of these species on our previous results. Because one of our main arguments was that dispersal ability determines the evolution of the shape of the SADs which, as we discussed in the main text, in mainly reflected in the skewness of the distributions, we focus on this attribute to summarize the impact of incorrectly assigning dispersal ability. Our conclusion is that it did not change our main findings, as we can see by comparing Fig. S5 with Fig. S9, which are virtually identical.

Although we are confident about our assessment of the dispersal ability of the large majority of species, in what follows we further conducted a more thorough sensitivity analyses. We consider four levels for the probability of a species dispersal ability being incorrectly identified, $Prob(W)$ = 0.06, 0.12, 0.18, 0.24, that is, ranging from our perceived possible error of 6% up to approximately one quarter of the species. Then, among the species that were incorrectly identified we consider six levels for the probability of a species being incorrectly identified as "High" instead of "Low", $Prob(H|W)$ = 0.0, 0.2, 0.4, 0.6, 0.8, 1.0 or, equivalently, the probability of being incorrectly identified as "Low" instead of "High", $Prob(L|W) = 1- P(H|W)$.

We use the data from Terceira island, the one with the largest number of transects, to illustrate the impact of incorrectly identifying the dispersal ability on the evolution of the SADs as described by the skewness. From Fig. S10 we conclude that only for the largest values of $Prob(W)$ = 0.18 and 0.24, and $Prob(H|W)$ equal to zero would our conclusions have been different, values of $Prob(W)$ and $Prob$(H|W) that we think are unlikely. Therefore, we believe that our results are not significantly impacted by any unlikely miss identification of a species dispersal ability.

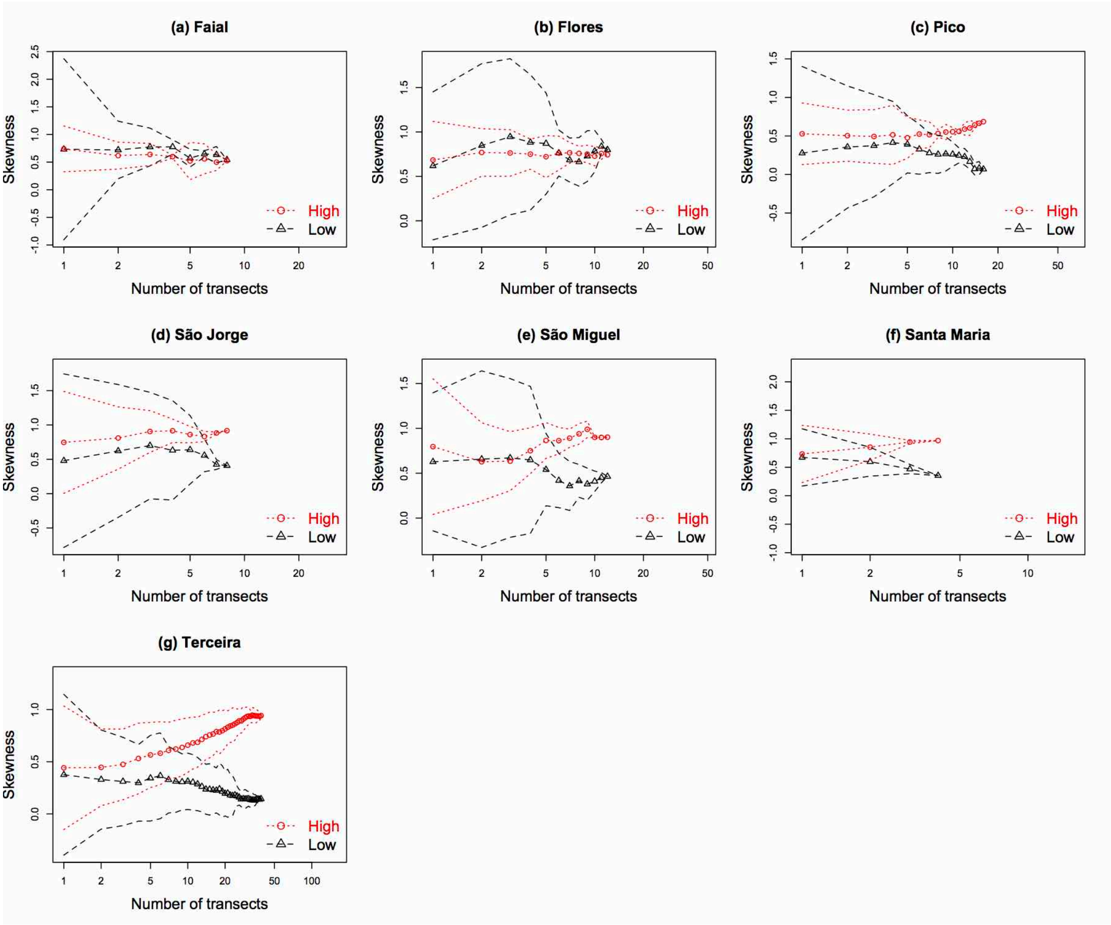

Figure S9. The evolution of the skewness as a function of the number of transects assuming an incorrect identification of the dispersal ability for the species listed in Table S2 and thus changing for these the dispersal trait from "high" to "low", and vice-versa.

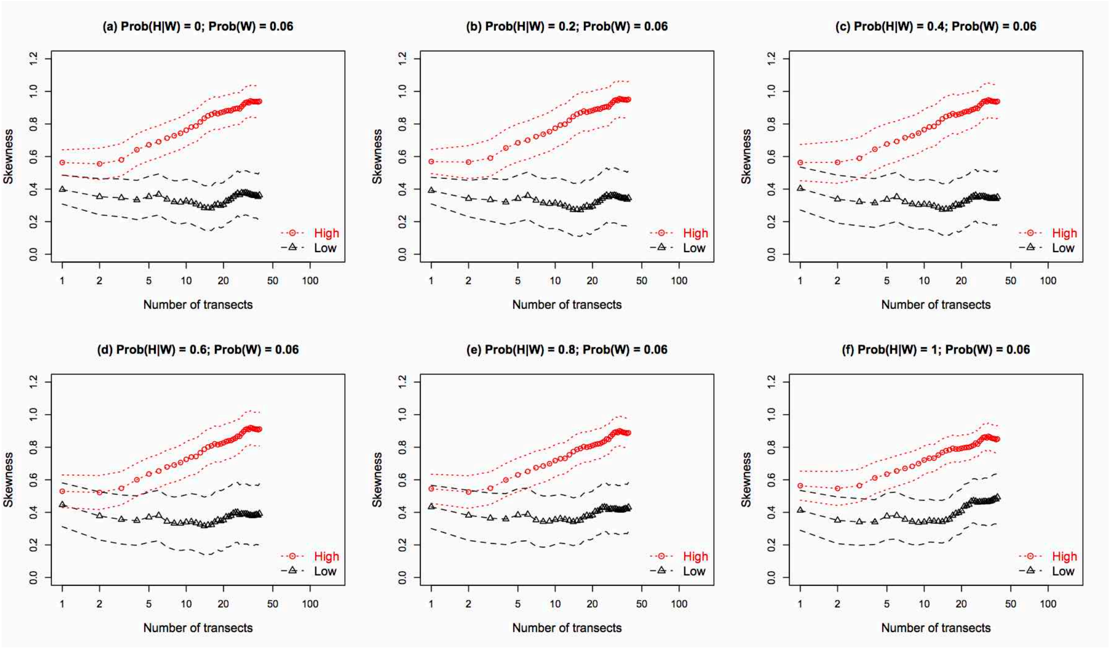

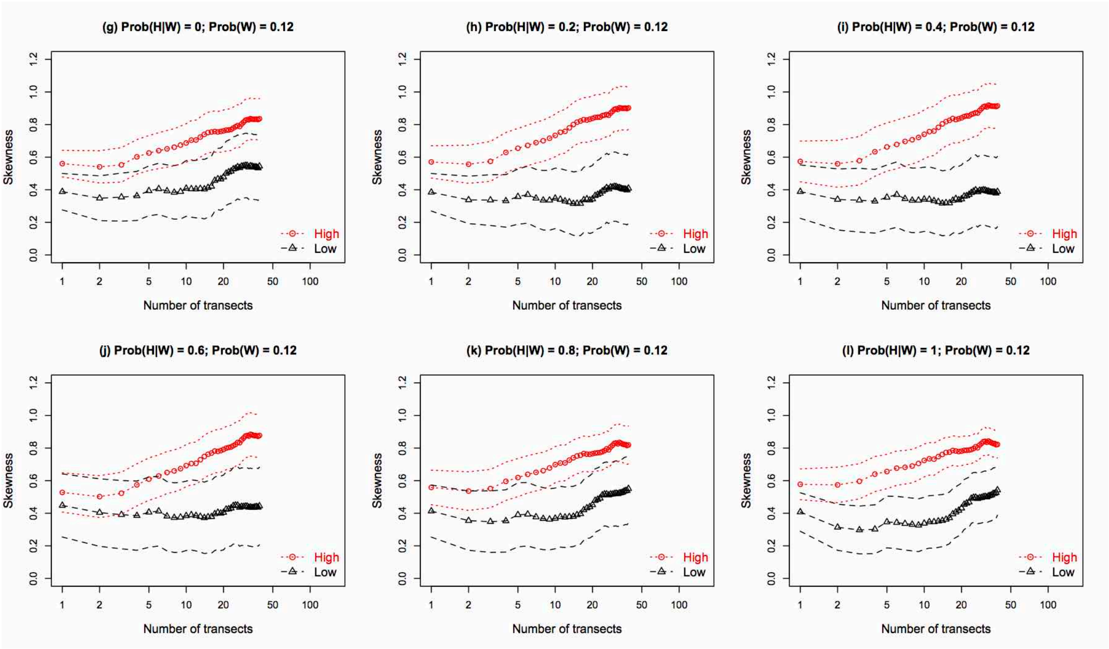
(g) Prob(H|W) = 0; Prob(W) = 0.12
(h) Prob(H|W) = 0.2; Prob(W) = 0.12
(i) Prob(H|W) = 0.4; Prob(W) = 0.12
(j) Prob(H|W) = 0.6; Prob(W) = 0.12
(k) Prob(H|W) = 0.8; Prob(W) = 0.12
(l) Prob(H|W) = 1; Prob(W) = 0.12
Skewness
0.0
0.2
0.4
0.6
0.8
1.0
1.2
1
2
5
10
20
50
100
Number of transects
High
Low

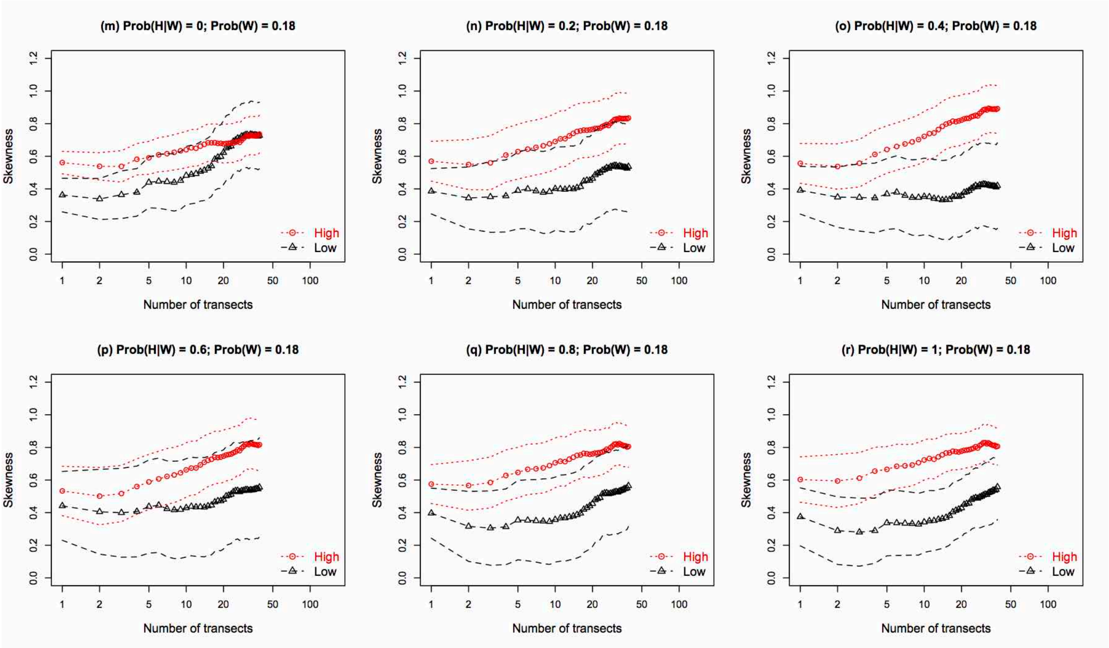
(m) Prob(H|W) = 0; Prob(W) = 0.18
(n) Prob(H|W) = 0.2; Prob(W) = 0.18
(o) Prob(H|W) = 0.4; Prob(W) = 0.18
(p) Prob(H|W) = 0.6; Prob(W) = 0.18
(q) Prob(H|W) = 0.8; Prob(W) = 0.18
(r) Prob(H|W) = 1; Prob(W) = 0.18
Skewness
0.0
0.2
0.4
0.6
0.8
1.0
1.2
1
2
5
10
20
50
100
Number of transects
High
Low

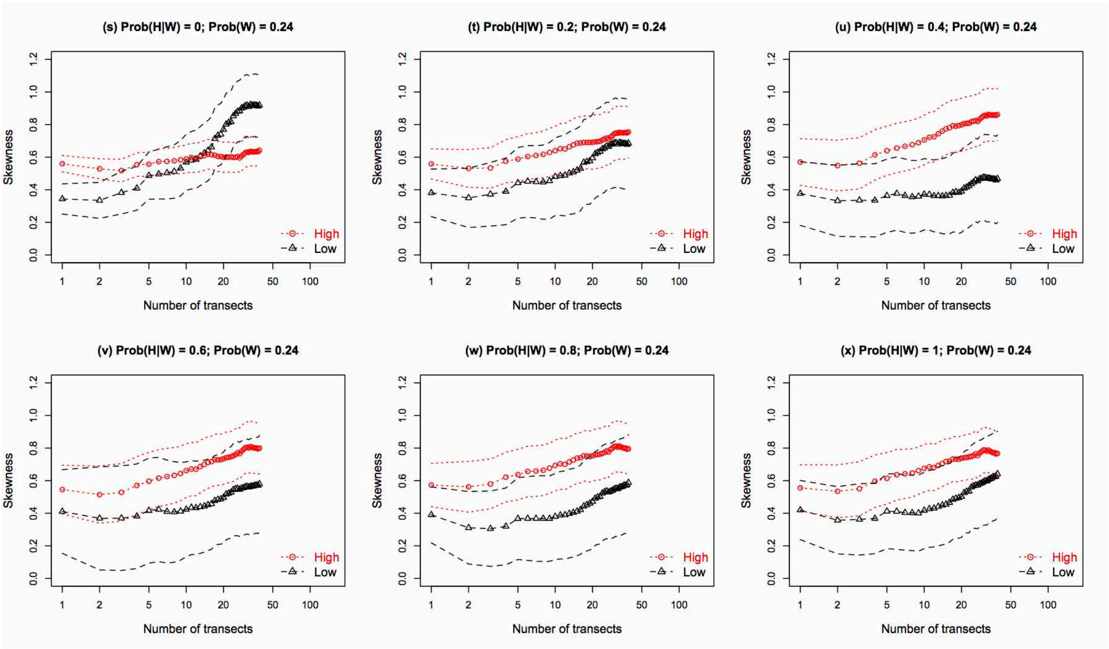

Figure S10. The evolution of the skewness as a function of the number of transects assuming different probabilities of incorrectly identifying a species, $P(W)$, and different probability for an incorrectly identified species being "High" while it should be "Low", $P(H|W)$. Plots a-f are for $P(W) = 0.06$, similar to the value estimated by us, g-l for $P(W) = 0.12$, m-r for $P(W) = 0.18$, and s-x for $P(W) = 0.24$. In each set of six plots $P(H|W) = 0.0, 0.2, 0.4, 0.6, 0.8, 1.0$.

Supplementary Table S2. List of doubtful species concerning their dispersal ability.

**Class**	**Order**	**Family**	**Species**	**Dispersal ability Attributed -> Alternative**
Arachnida	Araneae	Thomisidae	*Xysticus cor* Canestrini	Low -> High
Insecta	Lepidoptera	Geometridae	*Cyclophora pupillaria granti* Prout	Low -> High
Arachnida	Araneae	Linyphiidae	*Canariphantes acoreensis* (Wunderlich)	High -> Low
Insecta	Hemiptera	Coccidae	Gen. sp.	Low -> High
Insecta	Lepidoptera	Geometridae	*Cyclophora azorensis* (Prout)	Low -> High
Arachnida	Araneae	Dictynidae	*Lathys dentichelis* (Simon)	Low -> High
Insecta	Hemiptera	Coccidae	Gen. sp.	Low -> High
Arachnida	Araneae	Theridiidae	*Steatoda grossa* (C.L. Koch)	Low -> High
Insecta	Hemiptera	Coccidae	Gen. sp.	Low -> High
Arachnida	Araneae	Tetragnathidae	*Metellina merianae* (Scopoli)	Low -> High
Insecta	Coleoptera	Elateridae	*Athous pomboi* Platia & Borges	High -> Low
Insecta	Hemiptera	Coccidae	Gen. sp.	High -> Low
Insecta	Coleoptera	Elateridae	*Alestrus dolosus* (Crotch)	Low -> High
Arachnida	Araneae	Thomisidae	*Xysticus nubilus* Simon	High -> Low
Insecta	Hemiptera	Coccidae	Gen. sp.	High -> Low
Arachnida	Araneae	Theridiidae	Gen. sp.	High -> Low
Arachnida	Araneae	Araneidae	Gen. sp.	High -> Low

Insecta	Hemiptera	Coccidae	Gen. sp.	High -> Low
Insecta	Hemiptera	Coccidae	Gen. sp.	High -> Low

Supplementary Table S1. List of arthropod (morpho) species, with indication of their colonization status (E- endemic; N - native non-endemic; I - introduced), trophic ecology (H - Herbivorous; P - Predators) and dispersal ability (Low vs High).

**Class**	**Order**	**Family**	**Species**	**Colonization**	**Trophic**	**Dispersal**
Insecta	Lepidoptera	Tortricidae	Gen. sp.	I	H	Low
Arachnida	Araneae	Linyphiidae	*Tenuiphantes miguelensis Wunderlich*	N	P	High
Arachnida	Araneae	Thomisidae	*Xysticus cor Canestrini*	N	P	Low
Arachnida	Araneae	Linyphiidae	*Porrhomma borgesi Wunderlich*	E	P	Low
Arachnida	Araneae	Theridiidae	*Rugathodes acoreensis Wunderlich*	E	P	High
Arachnida	Opiliones	Phalangiidae	*Leiobunum blackwalli Meade*	N	P	High
Insecta	Hemiptera	Cixiidae	*Cixius azoterceirae Remane & Asche*	E	H	High
Insecta	Hemiptera	Cicadellidae	*Aphrodes hamiltoni Quartau & Borges*	E	H	High
Diplopoda	Julida	Julidae	*Ommatoiulus moreletii (Lucas)*	I	H	Low
Insecta	Lepidoptera	Noctuidae	*Mythimna unipuncta (Haworth)*	N	H	High
Insecta	Lepidoptera	Geometridae	*Cyclophora pupillaria granti Prout*	E	H	Low
Insecta	Hemiptera	Aleyrodidae	Gen. sp.	N?	H	High
Insecta	Coleoptera	Carabidae	*Ocys harpaloides (Audinet-Serville)*	N	P	High
Arachnida	Araneae	Lycosidae	*Pardosa acorensis Simon*	E	P	High
Insecta	Lepidoptera	Yponomeutidae	*Argyresthia atlanticella Rebel*	E	H	High
Arachnida	Araneae	Linyphiidae	*Palliduphantes schmitzi (Kulczynski)*	N	P	High
Arachnida	Araneae	Linyphiidae	*Tenuiphantes tenuis (Blackwall)*	I	P	High
Arachnida	Araneae	Linyphiidae	*Agyneta decora (O.P.-Cambridge)*	I	P	High
Insecta	Coleoptera	Staphylinidae	*Phloeonomus punctipennis Thomson*	N	P	High
Chilopoda	Geophilomorpha	Geophilidae	*Geophilus truncorum Bergsoe & Meinert*	N	P	Low
Chilopoda	Lithobiomorpha	Lithobiidae	*Lithobius pilicornis pilicornis Newport*	N	P	Low
Arachnida	Araneae	Dysderidae	*Dysdera crocata C. L. Koch*	I	P	Low
Insecta	Coleoptera	Curculionidae	*Otiorhynchus rugosostriatus (Goeze)*	I	H	Low
Insecta	Coleoptera	Carabidae	*Pterostichus vernalis (Panzer)*	I	P	High
Arachnida	Opiliones	Phalangiidae	*Homalenotus coriaceus (Simon)*	N	P	Low
Arachnida	Araneae	Linyphiidae	*Erigone atra Blackwall*	I	P	High
Arachnida	Pseudoscorpiones	Chthoniidae	*Chthonius ischnocheles (Hermann)*	I	P	Low
Arachnida	Araneae	Pisauridae	*Pisaura acoreensis Wunderlich*	E	P	Low
Insecta	Coleoptera	Staphylinidae	*Ocypus aethiops (Waltl)*	N	P	High
Insecta	Lepidoptera	Geometridae	*Nycterosea obstipata (Fabricius)*	N	H	Low
Insecta	Hemiptera	Lachnidae	*Cinara juniperi (De Geer)*	N	H	High
Insecta	Coleoptera	Carabidae	*Anisodactylus binotatus (Fabricius)*	I	P	High
Insecta	Coleoptera	Curculionidae	*Drouetius borgesi borgesi Machado*	E	H	Low
Arachnida	Araneae	Linyphiidae	*Canariphantes acoreensis (Wunderlich)*	E	P	High
Insecta	Coleoptera	Carabidae	*Paranchus albipes (Fabricius)*	I	P	High
Insecta	Hemiptera	Margarodidae	Gen. sp.	?	H	Low
Insecta	Dermaptera	Forficulidae	*Forficula auricularia Linnaeus*	I	P	Low
Insecta	Coleoptera	Staphylinidae	*Atheta aeneicollis (Sharp)*	I	P	High
Insecta	Hemiptera	Coccidae	Gen. sp.	I	H	Low

Insecta	Hemiptera	Aphididae	*Rhopalosiphonimus latysiphon (Davidson)*	I	H	High
Insecta	Coleoptera	Staphylinidae	*Sepedophilus lusitanicus (Hammond)*	N	P	High
Insecta	Coleoptera	Curculionidae	*Pseudechinosoma nodosum Hustache*	E	H	Low
Insecta	Coleoptera	Scraptiidae	*Anaspis proteus (Wollaston)*	N	H	High
Insecta	Coleoptera	Staphylinidae	*Quedius curtipennis Bernhauer*	N	P	High
Insecta	Hemiptera	Aphididae	*Aphis sp.*	?	H	High
Insecta	Coleoptera	Staphylinidae	*Proteinus atomarius Erichson*	N	P	High
Insecta	Hemiptera	Lygaeidae	*Plinthisus brevipennis (Latreille)*	N	H	High
Insecta	Coleoptera	Staphylinidae	*Aloconota sulcifrons (Stephens)*	N	P	High
Insecta	Coleoptera	Staphylinidae	*Ocypus olens (Muller)*	N	P	High
Insecta	Coleoptera	Staphylinidae	*Tachyporus nitidulus (Fabricius)*	I	P	High
Insecta	Lepidoptera	Geometridae	*Cyclophora azorensis (Prout)*	E	H	Low
Insecta	Lepidoptera	Tineidae	*Oinophila v-flava (Haworth)*	I	H	High
Insecta	Coleoptera	Laemophloeidae	*Placonotus sp.*	N	P	High
Insecta	Coleoptera	Staphylinidae	*Phloeonomus punctipennis Thomson*	I	P	High
Insecta	Hemiptera	Cydnidae	*Geotomus punctulatus (Costa)*	N	H	High
Insecta	Coleoptera	Curculionidae	*Pseudophloeophagus tenax (Wollaston)*	N	H	High
Arachnida	Pseudoscorpiones	Chthoniidae	*Chthonius tetrachelatus (Preyssler)*	I	P	Low
Insecta	Hemiptera	Aphididae	*Aphis craccivora Koch*	N	H	High
Insecta	Lepidoptera	Crambidae	*Scoparia coecimaculalis Warren*	E	H	High
Arachnida	Araneae	Dictynidae	*Lathys dentichelis (Simon)*	N	P	Low
Insecta	Hemiptera	Lygaeidae	*Scolopostethus decoratus (Hahn)*	N	H	High
Insecta	Hemiptera	Coccidae	Gen. sp.	I	H	Low
Insecta	Coleoptera	Curculionidae	*Coccotrypes carpophagus (Hornung)*	I	H	High
Arachnida	Araneae	Clubionidae	*Clubiona terrestris Westring*	I	P	Low
Insecta	Hemiptera	Flatidae	*Cyphopterum adcendens (Herr.-Schaff.)*	N	H	High
Insecta	Lepidoptera	Noctuidae	*Chrysodeixis chalcites (Esper)*	N	H	Low
Arachnida	Araneae	Theridiidae	*Steatoda grossa (C.L. Koch)*	I	P	Low
Insecta	Coleoptera	Curculionidae	*Caulotrupis parvus Israelson*	E	H	Low
Insecta	Lepidoptera	Gelechiidae	*Brachmia infuscatella Rebel*	E	H	High
Insecta	Hemiptera	Coccidae	Gen. sp.	I	H	Low
Arachnida	Araneae	Tetragnathidae	*Metellina merianae (Scopoli)*	I	P	Low
Arachnida	Araneae	Araneidae	*Gibbaranea occidentalis Wunderlich*	E	P	High
Insecta	Thysanoptera	Phlaeothripidae	*Nesothrips propinquus (Bagnall)*	I	H	High
Insecta	Hemiptera	Miridae	*Pinalitus oromii J. Ribes*	E	H	High
Insecta	Coleoptera	Elateridae	*Athous pomboi Platia & Borges*	E	H	High
Arachnida	Araneae	Mimetidae	*Ero furcata (Villers)*	I	P	Low
Insecta	Coleoptera	Curculionidae	*Calacalles subcarinatus (Israelson)*	E	H	High
Insecta	Coleoptera	Staphylinidae	*Tachyporus chrysomelinus (Linnaeus)*	I	P	High
Insecta	Coleoptera	Cerambycidae	*Crotchiella brachyptera Israelson*	E	H	High
Insecta	Coleoptera	Dryopidae	*Dryops luridus (Erichson)*	N	H	High
Insecta	Coleoptera	Staphylinidae	*Atheta zealandica Cameron, 9*	N	P	High
Insecta	Hemiptera	Aphididae	*Pseudacaudella rubida (Börner)*	N	H	High
Insecta	Hemiptera	Coccidae	Gen. sp.	I	H	Low

Insecta	Hemiptera	Delphacidae	*Muellerianella sp.*	N	H	High
Insecta	Hemiptera	Tingidae	*Acalypta parvula (Fallén)*	N	H	High
Insecta	Coleoptera	Staphylinidae	*Atheta sp.*	E	P	High
Insecta	Hemiptera	Lygaeidae	*Kleidocerys ericae (Horváth)*	N	H	High
Insecta	Hemiptera	Aphididae	*Rhopalosiphum padi (Linnaeus)*	I	H	High
Insecta	Lepidoptera	Geometridae	*Ascotis fortunata azorica Pinker*	E	H	Low
Arachnida	Araneae	Tetragnathidae	*Sancus acoreensis (Wunderlich)*	E	P	High
Arachnida	Araneae	Linyphiidae	*Savigniorrhipis acoreensis Wunderlich*	E	P	High
Insecta	Coleoptera	Staphylinidae	*Gabrius nigritulus (Gravenhorst)*	I	P	High
Insecta	Hemiptera	Triozidae	*Trioza (Lauritrioza) laurisilvae Hodkinson*	N	H	High
Arachnida	Araneae	Salticidae	*Macaroeris cata (Blackwall)*	N	P	Low
Insecta	Neuroptera	Hemerobiidae	*Hemerobius azoricus Tjeder*	E	P	High
Insecta	Lepidoptera	Crambidae	*Scoparia semiamplalis Warren*	E	H	High
Arachnida	Araneae	Theridiidae	*Lasaeola oceanica Simon*	E	P	Low
Insecta	Lepidoptera	Crambidae	*Eudonia luteusalis (Hampson)*	E	H	High
Insecta	Lepidoptera	Geometridae	Gen. sp.	E?	H	Low
Insecta	Hemiptera	Aphididae	*Neomyzus circumflexus (Buckton)*	I	H	High
Insecta	Coleoptera	Carabidae	*Trechus terrabravensis Borges, Serrano & Amorim*	E	P	Low
Arachnida	Araneae	Araneidae	*Mangora acalypha (Walckenaer)*	I	P	Low
Insecta	Hemiptera	Nabidae	*Nabis pseudoferus ibericus Remane*	N	P	High
Insecta	Hemiptera	Aphididae	*Acyrthosiphon pisum Harris*	N	H	High
Arachnida	Araneae	Linyphiidae	*Oedothorax fuscus (Blackwall)*	I	P	High
Arachnida	Araneae	Linyphiidae	*Erigone autumnalis Emerton*	I	P	High
Arachnida	Araneae	Linyphiidae	*Pelecopsis parallela (Wider)*	I	P	High
Insecta	Coleoptera	Curculionidae	*Xyleborinus alni Nijima*	I	H	High
Insecta	Coleoptera	Coccinellidae	Gen. sp.	I	P	High
Arachnida	Araneae	Theridiidae	*Theridion musivivum Schmidt*	N	P	Low
Chilopoda	Scolopendromorpha	Cryptopidae	*Cryptops hortensis Leach*	N	P	Low
Insecta	Coleoptera	Elateridae	*Alestrus dolosus (Crotch)*	E	H	High
Insecta	Coleoptera	Staphylinidae	*Aleochara bipustulata (Linnaeus)*	I	P	High
Insecta	Hemiptera	Saldidae	*Saldula palustris (Douglas)*	N	P	High
Arachnida	Araneae	Linyphiidae	*Mermessus bryantae (Ivie & Barrows)*	I	P	High
Arachnida	Araneae	Thomisidae	*Xysticus nubilus Simon*	I	P	Low
Insecta	Lepidoptera	Crambidae	Gen. sp.	N?	H	Low
Insecta	Coleoptera	Staphylinidae	*Phloeostiba azorica (Fauvel)*	E	P	High
Insecta	Hemiptera	Delphacidae	*Megamelodes quadrimaculatus (Signoret)*	N	H	High
Insecta	Hemiptera	Cixiidae	*Cixius insularis Lindberg*	E	H	High
Insecta	Coleoptera	Staphylinidae	*Rugilus orbiculatus orbiculatus (Paykull)*	N	P	High
Insecta	Coleoptera	Carabidae	*Calathus lundbladi Colas*	E	P	Low
Insecta	Coleoptera	Staphylinidae	*Anotylus nitidifrons (Wollaston)*	I	P	High
Insecta	Lepidoptera	Coleophoridae	*Opogona sp.*	I	H	High
Insecta	Lepidoptera	Noctuidae	*Agrotis sp.*	N	H	Low
Insecta	Hemiptera	Delphacidae	*Muellerianella sp.*	N	H	High

Insecta	Hemiptera	Delphacidae	Gen. sp.	N	H	High
Insecta	Thysanoptera	Thripidae	*Heliothrips haemorrhoidalis (Bouché)*	I	H	High
Insecta	Thysanoptera	Thripidae	*Hercinothrips bicinctus (Bagnall)*	I	H	High
Arachnida	Araneae	Oonopidae	*Orchestina furcillata Wunderlich*	E	P	Low
Diplopoda	Julida	Julidae	*Cylindroiulus propinquus (Porat)*	I	H	High
Insecta	Thysanoptera	Phlaeothripidae	*Apterygothrips n.sp.*	E	H	High
Insecta	Coleoptera	Dytiscidae	*Agabus godmani Crotch*	E	P	High
Insecta	Coleoptera	Dryopidae	*Dryops algiricus Lucas*	N	H	Low
Insecta	Lepidoptera	Nymphalidae	*Hipparchia miguelensis (Le Cerf)*	E	H	High
Insecta	Hemiptera	Aphididae	*Uroleucon erigeronense (Thomas)*	I	H	High
Insecta	Hemiptera	Aphididae	*Aulacorthum solani (Kaltenbach)*	N	H	High
Arachnida	Araneae	Linyphiidae	*Agyneta rugosa Wunderlich*	E	P	High
Insecta	Hemiptera	Cixiidae	*Cixius azopifajo azopifajo Remane & Asche*	E	H	High
Arachnida	Pseudoscorpiones	Neobisiidae	*Neobisium maroccanum Beier*	I	P	Low
Insecta	Coleoptera	Carabidae	*Pseudanchomenes aptinoides Tarnier*	E	P	Low
Chilopoda	Lithobiomorpha	Lithobiidae	*Lithobius sp.*	N	P	Low
Insecta	Hemiptera	Drepanosiphidae	*Anoecia corni (Fabricius)*	I	H	High
Insecta	Hemiptera	Aleyrodidae	Gen. sp.	N?	H	High
Insecta	Coleoptera	Carabidae	*Cedrorum azoricus caveirensis Borges & Serrano*	E	P	Low
Insecta	Coleoptera	Carabidae	*Pterostichus aterrimus aterrimus (Herbst)*	N	P	High
Insecta	Hemiptera	Coccidae	Gen. sp.	I	H	Low
Insecta	Coleoptera	Dryophthoridae	*Sitophilus oryzae (Linnaeus)*	I	H	High
Insecta	Lepidoptera	Noctuidae	*Phlogophora interrupta (Warren)*	E	H	Low
Arachnida	Araneae	Linyphiidae	*Acorigone acoreensis (Wunderlich)*	E	P	High
Arachnida	Araneae	Linyphiidae	*Agyneta depigmentata Wunderlich*	E	P	High
Insecta	Lepidoptera	Noctuidae	*Xestia c-nigrum (Linnaeus)*	N	H	High
Insecta	Hemiptera	Cixiidae	*Cixius azofloresi Remane & Asche*	E	H	High
Insecta	Lepidoptera	Crambidae	Gen. sp.	?	H	High
Insecta	Hemiptera	Aphididae	*Rhopalosiphum rufiabdominalis (Sasaki)*	I	H	High
Arachnida	Araneae	Zodariidae	*Zodarion atlanticum Pekár & Cardoso*	I	P	Low
Insecta	Hemiptera	Cicadellidae	*Opsius stactogallus Fieber*	N	H	High
Insecta	Hemiptera	Aphididae	*Rhopalosiphum oxyacanthae (Schrank)*	I	H	High
Insecta	Lepidoptera	Noctuidae	*Agrotis ipsilon (Hufnagel)*	N	H	High
Insecta	Lepidoptera	Nymphalidae	*Hipparchia azorina occidentalis (Sousa)*	E	H	High
Insecta	Coleoptera	Staphylinidae	*Quedius simplicifrons (Fairmaire)*	N	P	High
Insecta	Coleoptera	Carabidae	*Cedrorum azoricus azoricus Borges & Serrano*	E	P	Low
Arachnida	Araneae	Linyphiidae	*Eperigone sp.*	I	P	High
Insecta	Dermaptera	Anisolabididae	*Euborellia annulipes (Lucas)*	I	P	Low
Insecta	Coleoptera	Curculionidae	*Orthochaetes insignis (Aubé)*	N	H	High
Insecta	Thysanoptera	Thripidae	*Aptinothrips rufus Haliday*	I	H	High
Insecta	Hemiptera	Aphididae	*Covariella aegopodii (Scopoli)*	I	H	High
Insecta	Lepidoptera	Crambidae	Gen. sp.	?	H	High
Insecta	Lepidoptera	Crambidae	*Scoparia sp.*	E?	H	High

Insecta	Lepidoptera	Tortricidae	Gen. sp.	I	H	Low
Insecta	Lepidoptera	?	Gen. sp.	?	H	Low
Insecta	Coleoptera	Staphylinidae	*Carpelimus corticinus (Gravenhorst)*	N	P	High
Arachnida	Araneae	Linyphiidae	*Lessertia dentichelis (Simon)*	I	P	High
Insecta	Lepidoptera	Noctuidae	*Mesapamea storai (Rebel)*	E	H	High
Insecta	Coleoptera	Staphylinidae	*Stenus guttula guttula Müller*	N	P	High
Insecta	Coleoptera	Staphylinidae	*Phloeostiba azorica (Fauvel)*	E	P	High
Insecta	Hemiptera	Aphididae	*Myzus cerasi (Fabricius)*	I	H	High
Insecta	Lepidoptera	Noctuidae	Gen. sp.	?	H	High
Insecta	Coleoptera	Staphylinidae	*Phloeostiba azorica (Fauvel)*	E	P	High
Insecta	Hemiptera	Cicadellidae	Gen. sp.	?	H	High
Arachnida	Araneae	Theridiidae	Gen. sp.	?	P	Low
Insecta	Lepidoptera	Tortricidae	Gen. sp.	I	H	Low
Insecta	Hemiptera	Aleyrodidae	Gen. sp.	?	H	High
Insecta	Lepidoptera	Geometridae	*Xanthorhoe inaequata (Warren)*	E	H	Low
Insecta	Hemiptera	Cixiidae	*Cixius azoricus azoricus Lindberg*	E	H	High
Insecta	Lepidoptera	Tortricidae	Gen. sp.	I	H	Low
Arachnida	Araneae	Linyphiidae	*Walckenaeria grandis (Wunderlich)*	E	P	High
Insecta	Lepidoptera	Crambidae	*Scoparia sp.*	E	H	High
Insecta	Lepidoptera	Noctuidae	Gen. sp.	N?	H	Low
Insecta	Hemiptera	Aleyrodidae	Gen. sp.	?	H	High
Arachnida	Araneae	Clubionidae	*Cheiracanthium floresense Wunderlich*	E	P	Low
Insecta	Trichoptera	Limnephilidae	*Limnephilus atlanticus Nybom*	E	P	High
Insecta	Coleoptera	Staphylinidae	*Atheta dryochares Israelson*	E	P	High
Insecta	Lepidoptera	Tortricidae	*Rhopobota naevana (Hübner)*	I	H	High
Arachnida	Araneae	Linyphiidae	*Minicia floresensis Wunderlich*	E	P	High
Arachnida	Araneae	Linyphiidae	Gen. sp.	E	P	High
Arachnida	Araneae	Linyphiidae	Gen. sp.	?	P	High
Arachnida	Araneae	Theridiidae	Gen. sp.	?	P	Low
Insecta	Trichoptera	?	Gen. sp.	?	P	Low
Arachnida	Araneae	Linyphiidae	*Erigone sp.*	?	P	High
Insecta	Coleoptera	Carabidae	*Acupalpus dubius Schilsky*	N	P	High
Arachnida	Araneae	Linyphiidae	*Mermessus trilobatus (Emerton)*	I	P	High
Insecta	Hemiptera	Cicadellidae	*Eupteryx azorica Ribaut*	E	H	High
Insecta	Lepidoptera	?	Gen. sp.	?	H	High
Insecta	Thysanoptera	Thripidae	*Ceratothrips ericae (Haliday)*	N	H	High
Arachnida	Araneae	Araneidae	Gen. sp.	I?	P	Low
Insecta	Coleoptera	Staphylinidae	*Habrocerus capillaricornis (Gravenhorst)*	N	P	High
Insecta	Hemiptera	Miridae	*Monalocoris filicis (Linnaeus)*	N	H	High
Insecta	Hemiptera	Aleyrodidae	Gen. sp.	?	H	High
Insecta	Hemiptera	Margarodidae	Gen. sp.	?	H	Low
Arachnida	Araneae	Theridiidae	*Cryptachaea blattea (Urquhart)*	I	P	Low
Insecta	Ephemeroptera	Baetidae	*Cloeon dipterum (Linnaeus)*	N	H	High
Insecta	Hemiptera	Lygaeidae	*Nysius atlantidum Horváth*	E	H	High

Insecta	Hemiptera	Microphysidae	*Loricula coleoptrata (Fallén)*	N	P	High
Insecta	Hemiptera	Aleyrodidae	Gen. sp.	E?	H	High
Arachnida	Araneae	Clubionidae	*Clubiona decora Blackwall*	N	P	Low
Insecta	Lepidoptera	Tortricidae	Gen. sp.	I	H	Low
Insecta	Hemiptera	Anthocoridae	*Brachysteles parvicornis (A. Costa)*	N	P	High
Arachnida	Araneae	Salticidae	*Neon acoreensis Wunderlich*	E	P	Low
Insecta	Hemiptera	Microphysidae	*Loricula elegantula (Bärensprung)*	N	P	High
Insecta	Hemiptera	Aphididae	*Toxoptera aurantii (Boyer de Fonscolombe)*	I	H	High
Insecta	Hemiptera	Miridae	*Heterotoma planicornis (Pallas)*	N	P	High
Insecta	Hemiptera	Reduviidae	*Empicoris rubromaculatus (Blackburn)*	I	P	High
Arachnida	Araneae	Dictynidae	*Emblyna acoreensis Wunderlich*	E	P	Low
Insecta	Coleoptera	Staphylinidae	*Scopaeus sp.*	N	P	High
Arachnida	Araneae	Linyphiidae	*Agyneta sp.*	?	P	High
Insecta	Hemiptera	Psyllidae	*Strophingia harteni Hodkinson*	E	H	High
Arachnida	Araneae	Theridiidae	*Rhomphaea nasica (Simon)*	I	P	Low
Insecta	Coleoptera	Curculionidae	*Phloeosinus gillerforsi Bright*	E	H	High
Insecta	Coleoptera	Nitidulidae	*Meligethes aeneus (Fabricius)*	I	H	High
Insecta	Hemiptera	Coccidae	Gen. sp.	I	H	Low
Insecta	Hemiptera	Miridae	*Campyloneura virgula (Herrich-Schaeffer)*	N	P	High
Arachnida	Araneae	Theridiidae	*Neottiura bimaculata (Linnaeus)*	I	P	Low
Arachnida	Araneae	Salticidae	*Macaroeris sp.*	I?	P	High
Insecta	Lepidoptera	Noctuidae	Gen. sp.	N?	H	Low
Insecta	Coleoptera	Dytiscidae	*Agabus bipustulatus (Linnaeus)*	N	P	High
Insecta	Hemiptera	Cixiidae	*Cixius azopifajo azojo Remane & Asche*	E	H	High
Arachnida	Araneae	Linyphiidae	*Acorigone zebraneus Wunderlich*	E	P	High
Insecta	Hemiptera	Delphacidae	*Muellerianella sp.*	N	H	High
Insecta	Lepidoptera	Noctuidae	Gen. sp.	N?	H	Low
Insecta	Lepidoptera	?	Gen. sp.	N?	H	Low
Insecta	Hemiptera	Miridae	*Polymerus cognatus (Fieber)*	N	H	High
Insecta	Lepidoptera	?	Gen. sp.	?	H	Low
Insecta	Hemiptera	Margarodidae	Gen. sp.	?	H	Low
Insecta	Lepidoptera	Tortricidae	Gen. sp.	N?	H	Low
Insecta	Orthoptera	Conocephalidae	*Conocephalus chavesi (Bolivar)*	E	H	High
Insecta	Hemiptera	Cixiidae	*Cixius azopifajo azofa Remane & Asche*	E	H	High
Insecta	Hemiptera	Aphididae	*Amphorophora rubi (Kaltenbach) sensu latiore*	N	H	High
Arachnida	Araneae	Dictynidae	*Nigma puella (Simon)*	I	P	Low
Insecta	Lepidoptera	?	Gen. sp.	N?	H	Low
Insecta	Hemiptera	Aleyrodidae	Gen. sp.	E?	H	High
Insecta	Lepidoptera	?	Gen. sp.	E?	H	Low
Insecta	Hemiptera	Cercopidae	*Philaenus spumarius (L.)*	I	H	High
Insecta	Hemiptera	Cixiidae	*Cixius azoricus azoropicoi Remane & Ashe*	E	H	High
Insecta	Hemiptera	Aleyrodidae	Gen. sp.	E?	H	High
Insecta	Lepidoptera	?	Gen. sp.	?	H	High
Insecta	Thysanoptera	Aeolothripidae	*Aeolothrips gloriosus Bagnall*	N	P	High

Insecta	Lepidoptera	Noctuidae	Gen. sp.	N?	H	Low
Insecta	Thysanoptera	Thripidae	*Thrips atratus Haliday*	N	H	High
Insecta	Hemiptera	Anthocoridae	*Orius laevigatus laevigatus (Fieber)*	N	P	High
Arachnida	Araneae	Oecobiidae	*Oecobius navus Blackwall*	I	P	Low
Insecta	Coleoptera	Staphylinidae	*Amischa analis (Gravenhorst)*	I	P	High
Insecta	Lepidoptera	?	Gen. sp.	I?	H	Low
Insecta	Hemiptera	Psyllidae	*Acizzia uncatoides (Ferris & Klyver)*	I	H	High
Insecta	Hemiptera	Psyllidae	*Cacopsylla pulchella (Low)*	I	H	High
Arachnida	Araneae	Araneidae	*Araneus sp.*	I	P	Low
Insecta	Coleoptera	Coccinellidae	*Lindorus lophanthae (Blaisdell)*	I	P	High
Insecta	Coleoptera	Staphylinidae	*Amischa analis (Gravenhorst)*	I	P	High
Insecta	Lepidoptera	Blastobasidae	*Blastobasis sp.*	N	H	High
Insecta	Thysanoptera	Thripidae	*Isoneurothrips australis Bagnall*	I	H	High
Insecta	Coleoptera	Staphylinidae	*Astenus lyonessius (Joy)*	N	P	High
Insecta	Coleoptera	Curculionidae	*Gymnetron pascuorum (Gyllenhal)*	I	H	High
Insecta	Hemiptera	Lygaeidae	*Microplax plagiata (Fieber)*	N	H	High
Insecta	Hemiptera	Aleyrodidae	Gen. sp.	E?	H	High
Insecta	Coleoptera	Chrysomelidae	Gen. sp.	I?	H	High
Insecta	Lepidoptera	?	Gen. sp.	E?	H	Low
Arachnida	Araneae	Linyphiidae	*Araeoncus n. sp.*	E	P	High
Insecta	Coleoptera	Carabidae	*Stenolophus teutonus (Schrank)*	N	P	High
Insecta	Hemiptera	?	Gen. sp.	E?	H	High
Insecta	Coleoptera	Dytiscidae	*Hydroporus guernei Régimbart*	E	P	High
Arachnida	Araneae	Clubionidae	*Clubiona genevensis L. Koch*	I	P	Low
Insecta	Lepidoptera	Crambidae	*Scoparia sp.*	E	H	High
Insecta	Coleoptera	Curculionidae	Gen. sp.	I	H	High
Arachnida	Araneae	Linyphiidae	*Microlinyphia johnsoni (Blackwall)*	N	P	High
Insecta	Lepidoptera	?	Gen. sp.	N?	H	Low
Insecta	Coleoptera	Laemophloeidae	Gen. sp.	I	P	High
Insecta	Coleoptera	Staphylinidae	*Atheta sp.*	E	P	High
Insecta	Thysanoptera	Phlaeothripidae	*Eurythrips tristis Hood*	I	H	High
Insecta	Hemiptera	Coccidae	Gen. sp.	I	H	Low
Insecta	Coleoptera	Nitidulidae	*Meligethes sp.*	I	H	High
Insecta	Lepidoptera	Blastobasidae	*Neomariania sp.*	I	H	High
Insecta	Hemiptera	Aleyrodidae	Gen. sp.	E?	H	High
Insecta	Lepidoptera	Gracillariidae	*Caloptilia schinella (Walsingham)*	I	H	High
Insecta	Hemiptera	Aleyrodidae	Gen. sp.	E?	H	High
Arachnida	Araneae	Linyphiidae	*Neriene clathrata (Sundevall)*	I	P	High
Insecta	Hemiptera	Pentatomidae	*Nezara viridula (Linnaeus)*	I	H	High
Insecta	Lepidoptera	Noctuidae	Gen. sp.	N?	H	Low
Arachnida	Araneae	Theridiidae	Gen. sp.	E?	P	Low
Insecta	Hemiptera	Aleyrodidae	Gen. sp.	E?	H	High
Insecta	Lepidoptera	?	Gen. sp.	N?	H	High
Insecta	Hemiptera	Aleyrodidae	Gen. sp.	E?	H	High
Insecta	Coleoptera	Carabidae	*Acupalpus flavicollis (Sturm)*	N	P	High

Insecta	Hemiptera	Lygaeidae	*Beosus maritimus (Scopoli)*	N	H	High
Insecta	Lepidoptera	Noctuidae	Gen. sp.	I	H	Low
Insecta	Lepidoptera	Tineidae	*Opogona sacchari (Bojer)*	I	H	High
Insecta	Thysanoptera	Phlaeothripidae	*Hoplandrothrips consobrinus (Knechtel)*	I	H	High
Insecta	Thysanoptera	Thripidae	*Thrips flavus Schrank*	N	H	High
Insecta	Hemiptera	Psyllidae	Gen. sp.	I	H	Low
Insecta	Lepidoptera	?	Gen. sp.	N	H	Low
Insecta	Coleoptera	Staphylinidae	*Xantholinus longiventris Heer*	I	P	High
Insecta	Thysanoptera	Aeolothripidae	*Aeolothrips collaris Priesner*	N	P	High
Insecta	Coleoptera	Nitidulidae	*Meligethes sp.*	I	H	High
Insecta	Coleoptera	Staphylinidae	*Atheta nigra (Kraatz)*	I	P	High
Insecta	Hemiptera	Lygaeidae	*Gastrodes grossipes grossipes (De Geer)*	I	H	High
Insecta	Thysanoptera	Thripidae	*Frankliniella sp.*	N	H	High
Insecta	Hemiptera	Lygaeidae	*Heterogaster urticae (Fabricius)*	N	H	High
Insecta	Coleoptera	Coccinellidae	*Coccinella undecimpunctata undecimpunctata Linnaeus*	I	P	High
Insecta	Lepidoptera	?	Gen. sp.	N?	H	Low
Insecta	Lepidoptera	?	Gen. sp.	N?	H	Low
Insecta	Coleoptera	Staphylinidae	*Atheta palustris (Kiesenwetter)*	I	P	High
Insecta	Thysanoptera	Phlaeothripidae	*Apterygothrips canarius (Priesner)*	I	H	High
Insecta	Hemiptera	Delphacidae	Gen. sp.	N	H	High
Insecta	Coleoptera	Staphylinidae	*Carpelimus troglodytes troglodytes (Erichson)*	I	P	High
Insecta	Lepidoptera	Blastobasidae	*Blastobasis sp.*	N	H	High
Insecta	Hemiptera	Lygaeidae	*Plinthisus minutissimus Fieber*	N	H	High
Insecta	Lepidoptera	?	Gen. sp.	N?	H	Low
Insecta	Hemiptera	Cixiidae	*Cixius azomariae Remane & Asche*	E	H	High
Insecta	Hemiptera	Cicadellidae	*Anoscopus albifrons (Linnaeus)*	N	H	High
Arachnida	Araneae	Clubionidae	*Cheiracanthium erraticum (Walckenaer)*	I	P	Low
Arachnida	Araneae	Clubionidae	*Cheiracanthium jorgeense Wunderlich*	E	P	Low
Insecta	Lepidoptera	Gracillariidae	*Micrurapteryx bistrigella (Rebel)*	E	H	High
Insecta	Hemiptera	Miridae	*Closterotomus norwegicus (Gmelin)*	N	H	High
Insecta	Hemiptera	Aphididae	Gen. sp.	I	H	High
Insecta	Thysanoptera	Phlaeothripidae	Gen. sp.	?	H	High
Insecta	Coleoptera	Curculionidae	Gen. sp.	?	H	High
Insecta	Coleoptera	Curculionidae	*Drouetius borgesi sanctmichaelis Machado*	E	H	Low
Insecta	Coleoptera	Curculionidae	*Drouetius borgesi centralis Machado*	E	H	Low
